# Effects of West Nile Virus on behavioral and cognitive performance, circadian body temperatures, cortical Aβ and p-tau pathology, and immune measures in human apoE and human amyloid precursor protein NL-G-F/human apoE mice

**DOI:** 10.3389/fimmu.2026.1899626

**Published:** 2026-10-02

**Authors:** Emily Bunnell, Elizabeth Saltonstall, Ella Shanava, Abigail O'Niel, Alexandra Pederson, Shriya Marla, Mariana Lopes Soares Llamas, Isha Garg, Mitali Chaudhari, Saanvi Vodapally, Henry F Harrison, Christopher J. Parkins, Alec J. Hirsch, Jacob Raber

**Affiliations:** 1 Department of Behavioral Neuroscience, Oregon Health & Science University, Portland, OR, United States; 2 Vaccine and Gene Therapy Center, ONPRC, Oregon Health & Science University, Beaverton, OR, United States; 3 Department of Neurology, Oregon Health & Science University, Portland, OR, United States; 4 Department of Radiation Medicine, Oregon Health & Science University, Portland, OR, United States; 5 Division of Neuroscience, ONPRC, Oregon Health & Science University, Portland, OR, United States

**Keywords:** amyloid pathology, amyloid precursor protein, apolipoprotein E, behavioral testing, body temperature, cognitive testing, immune measures, tau pathology

## Abstract

**Introduction:**

There are three human apolipoprotein E (E) isoforms: E2, E3, and E4. Compared with E3, E4 is an AD risk factor. West Nile Virus (WNV) can cause neurological disease, and it results in some neuropathology and neuroinflammation seen in Alzheimer’s disease (AD). Previously, we reported on the effects of exposure to the Kunjin strain (KUNV) of WNV in middle-aged knock-in (KI) mice expressing human amyloid precursor protein (APP) containing the dominant NL-G-F mutations with human apoE targeted replacement (TR) mice on behavioral, immune, and pathological measures, and circadian body temperatures. NL-G-F/E4 mice were more susceptible to KUNV-induced cognitive injury and prolonged viral load in the cortex; viral load was detected in the cortex of more mice expressing amyloid precursor protein with the dominant NL-G-F AD mutations and expressing also E4 than those expressing also E3. As NL-G-F and NL-G-F/apoE mice show cognitive injury and neuropathology at 6 months of age and age is a risk factor to develop AD and modulates the immune response in AD, we assessed in this study whether genotype differences in response to KUNV exposure are seen in younger mice. To determine whether the presence of NL-G-F is required to detect genotype differences in response to KUNV and/or modulates the response to KUNV, E3 and E4 mice were included as well. As compared with E3, E2 reduces the risk to develop AD and the vast majority of AD would likely not occur in the absence of E3 and E4 NL-G-F/E2 mice were included in this study as well.

**Methods:**

We crossed knock-in (KI) mice expressing human amyloid precursor protein (APP) containing the dominant NL-G-F mutations with human apoE targeted replacement (TR) mice and used middle-aged NL-G-F/E3 and NL-G-F/E4 mice to assess the role of WNV (subtype Kunjin virus) (KUNV) exposure on behavioral alteration performance, circadian body temperatures, viral loads, neuropathology, and transcript levels of three immune measures in cortex important in the detrimental effects of KUNV on brain function.

**Results:**

There were apoE isoform-dependent responses to KUNV exposure on various measures already seen at 6 months of age and are mostly more profound in the absence than in the presence of NL-G-F. One week following exposure, cortical insoluble p-tau levels were lower in KUNV- than PBS-treated apoE, but not NL-G-F/apoE mice. Soluble p-tau levels in cortex were higher in KUNV- than PBS-treated mice, and this effect was more pronounced in NL-G-F/apoE than apoE mice. The insoluble/soluble p-tau ratio was lower in KUNV- than PBS-treated mice, and this effect was more pronounced in apoE than NL-G-F/apoE.

**Discussion:**

These results are consistent with results in cultured cells supporting that Aβ and p-tau might have antiviral properties.

## 1 Introduction

Amyloid pathology, consisting of the human amyloid peptides Aβ40 and Aβ42 generated from amyloid precursor protein (APP), and hyperphosphorylated tau (p-tau) are the hallmarks of Alzheimer’s disease (AD) (1–3). Exposure to neurotropic viruses like Herpes Simplex Virus 1 (HSV-1) (4) has been hypothesized to contribute to the onset and progression of AD pathology and other neurodegenerative conditions like amyotrophic lateral sclerosis (ALS), multiple sclerosis (MS), Parkinson’s disease (PD), and vascular dementia (VAS) (5, 6). More recently, Aβ (7) and p-tau (8) have been shown to be increased in neuronal cultures exposed to HSV-1 (9) and to have antiviral properties. Following HSV-1 infection, there is a dose-dependent increase in the insoluble-to-soluble phosphorylated tau (p-tau) ratio and total tau levels without an increase in cytotoxicity or cell death, a decrease in intracellular soluble p-tau, an increase of the p-tau to total tau ratio in the cell media, and a decrease of this ratio in the cell lysates. Aβ pathology and hyperphosphorylated tau might limit viral replication and spread in the brain. These effects are not limited to HSV-1, as effects have also been reported for HCMV (10) and human herpes viruses 6A and 6B (7). Antibacterial effects of Aβ and tau have also been reported in response to *Staphylococcus aureus* and *Escherichia coli* (11) and *Salmonella* Typhimurium (12), or limited to AD (13). While tau hyperphosphorylation, microtubule destabilization and aggregation, and the spread of hyperphosphorylated tau might limit viral replication and spread in the brain, this immune and injury response will likely be at the expense of behavioral and cognitive performance. In addition, these data suggest that caution might be needed to pursue strategies to reduce or even eliminate hyperphosphorylated tau (14–16) and amyloid pathology (17–19) as it might make the brain more susceptible to viral infection or viral recrudescence.

There are three major human apolipoprotein E (E) isoforms (20), E2, E3, and E4. Compared with E3, E2 is protective whereas E4 is harmful with regard to AD risk (21–23). The bidirectional relationship between Aβ and p-tau pathology and viral exposure and spread in the brain is potentially modulated by apolipoprotein E in an isoform-dependent fashion. For example, the reduced ability of E4 compared with E3 to limit viral replication might contribute to the increased AD risk of E4 carriers; typically, more viral replication is seen in E4 than E3 carriers; in a mouse model of HSV-1, the cerebral load of latent HSV-1 genomic copies, which is associated with the reactivation risk (24), is 10-fold higher in E4 than E3 mice (25). E4 is also associated with enhanced entry of human immunodeficiency virus 1 (HIV-1) and cell entry and HIV-1 disease progression (26). Apolipoprotein E is an HIV-1-inducible inhibitor of viral production and infectivity in macrophages (27) and involved in the pathogenesis and susceptibility to other infectious diseases, including HSV-1, hepatitis C virus, hepatitis E virus, varicella zoster virus, Epstein–Barr virus, malaria, *Listeria monocytogenes* (LM), and *Klebsiella pneumoniae* (27).

West Nile Virus (WNV) can cause severe and long-lasting neurological disease (28) (29) (30) (31) and after initial infection in the hippocampus result in neuropathology and neuroinflammation seen in AD (28). In C57BL/6J wild-type (WT) mice after exposure to an attenuated WNV strain, there are hippocampus-dependent spatial learning impairments (32). The impact of WNV on AD-relevant behavioral and cognitive performance, neuropathology, and neuroinflammation might be modulated by AD-susceptibility genes. There are apoE isoform differences in binding to very-low-density lipoproteins (VLDL), with E4 binding better than E3 (33), and the binding of WNV capsid protein to VLDL is hypothesized to be important for uptake and transport of virus (34). We crossed knock-in (KI) mice expressing human amyloid precursor protein (APP) containing the dominant NL-G-F mutations (35, 36) with human apoE targeted replacement (TR) mice (37, 38) and used middle-aged NL-G-F/E3 and NL-G-F/E4 mice (39) to assess the role of prior exposure to the Kunjin strain (KUNV) of WNV exposure on hAPP/Aβ-induced behavioral alterations, cognitive injury, circadian body temperatures, and neuropathology and whether these effects are apoE isoform-dependent and associated with enhanced neuroinflammation in the hippocampus or cortex (40). Consistent with the studies described above, NL-G-F/E4 mice were more susceptible to KUNV-induced cognitive injury and prolonged viral load in the cortex; viral load was detected in the cortex of more mice expressing amyloid precursor protein with the dominant NL-G-F AD mutations and expressing also E4 than those expressing also E3. As NL-G-F (35, 36, 41, 42) and NL-G-F/apoE (39) mice show cognitive injury and neuropathology at 6 months of age and age is a risk factor to develop AD and modulates the immune response in AD (43), we assessed whether genotype differences in response to KUNV exposure are also seen in younger mice. We tested the central hypothesis that the presence of NL-G-F is required to detect genotype differences in response to KUNV and modulates the response to KUNV exposure and therefore included E3 and E4 mice without NL-G-F as well. As compared with E3, E2 reduces the risk to develop AD (23) and the vast majority of AD would likely not occur in the absence of E3 and E4 (44), we also included NL-G-F/E2 mice in this study.

## 2 Materials and methods

### 2.1 Mice and implantation of temperature sensors

Targeted replacement (TR) E2 (38), E3 (37), and E4 (38) mice crossed with NL-G-F mice (36) and backcrossed to only contain human APP and human apoE (39, 45) and regular E3 and E4 mice were used for the current study.

#### 2.1.1 Analysis 1

Analysis 1 contained young mice, *n* = 128 mice (5.94 ± 0.18 months); *n* = 17 E3 mice (4.45 ± 0.38 months) (*n* = 8 PBS-treated mice 3.21 ± 0.00 months) (*n* = 5 male mice and *n* = 3 female mice) and n = 9 KUNV-treated mice 5.54 ± 0.49 months) (*n* = 5 male mice and *n* = 4 female mice); *n* = 32 E4 mice 4.89 ± 0.22 months) (*n* = 15 PBS-treated mice 5.52 ± 0.39 months) (*n* = 8 male mice and *n* = 7 female mice) and *n* = 17 KUNV-treated mice 4.33 ± 0.16 months) (*n* = 10 male mice and *n* = 7 female mice); *n* = 22 NL-G-F/E2 mice 5.82 ± 0.37 months) (*n* = 11 PBS-treated mice 4.91 ± 0.29 months) (*n* = 5 male mice and *n* = 6 female mice) and *n* = 11 KUNV-treated mice 6.74 ± 0.57 months) (*n* = 5 male mice and *n* = 6 female mice); *n* = 21 NL-G-F/E3 mice 6.77 ± 0.55 months) (*n* = 8 PBS-treated mice 5.42 ± 0.92 months) (*n* = 4 male mice and *n* = 4 female mice) and *n* = 13 KUNV-treated mice 7.60 ± 0.60 months) (*n* = 5 male mice and *n* = 8 female mice); and *n* = 36 NL-G-F/E4 mice 7.16 ± 0.31 months) (*n* = 19 PBS-treated mice 7.49 ± 0.35 months) (*n* = 12 male mice and *n* = 7 female mice) and *n* = 17 KUNV-treated mice 6.79 ± 0.52 months) (*n* = 8 male mice and *n* = 9 female mice).

Analysis 2 included the young mice described above, older NL-G-F/E3 and NL-G-F/E4 mice reported earlier, and other young and older mice with additional genotypes (NL-G-F/E2, E3, and E4), *n* = 207 mice 8.86 ± 0.30 months); *n* = 27 E3 mice 7.51 ± 0.82 months) (*n* = 12 PBS-treated mice 6.51 ± 1.41 months) (*n* = 5 male mice and *n* = 7 female mice) and *n* = 15 KUNV-treated mice 8.32 ± 0.96 months) (*n* = 8 male mice and *n* = 7 female mice); *n* = 46 E4 mice 7.86 ± 0.69 months) (*n* = 24 PBS-treated mice 8.93 ± 0.96 months) (*n* = 15 male mice and *n* = 9 female mice) and *n* = 22 KUNV-treated mice 6.70 ± 0.96 months) (*n* = 14 male mice and *n* = 8 female mice); *n* = 33 NL-G-F/E2 mice 8.26 ± 0.66 months) (*n* = 16 PBS-treated mice 7.43 ± 0.99 months) (*n* = 9 male mice and *n* = 7 female mice) and *n* = 17 KUNV-treated mice 9.05 ± 0.87 months) (*n* = 9 male mice and *n* = 8 female mice); *n* = 36 NL-G-F/E3 mice 8.92 ± 0.60 months) (*n* = 15 PBS-treated mice 8.98 ± 1.25 months) (*n* = 4 male mice and *n* = 11 female mice) and *n* = 21 KUNV-treated mice 8.87 ± 0.53 months) (*n* = 10 male mice and *n* = 11 female mice); *n* = 65 NL-G-F/E4 mice 10.41 ± 0.51 months) (*n* = 33 PBS-treated mice 10.32 ± 0.65 months) (*n* = 12 male mice and *n* = 21 female mice) and *n* = 32 KUNV-treated mice 10.50 ± 0.79 months) (*n* = 12 male mice and *n* = 20 female mice).

While all mice received a sensor implant, due to the battery life of the sensors, not all sensors were able to collect data. Because of this, only a subset of mice was implanted with temperature sensors. Analysis 1 contained younger mice with sensors, *n* = 63 mice (7.01 ± 0.27 months); *n* = 16 E4 mice 4.67 ± 0.40 months) (*n* = 5 PBS-treated mice 6.36 ± 0.93 months) (*n* = 2 male mice and *n* = 3 female mice) and *n* = 11 KUNV-treated mice 3.90 ± 0.00 months) (*n* = 7 male mice and *n* = 4 female mice); *n* = 11 NL-G-F/E2 mice 6.24 ± 0.66 months) (*n* = 4 PBS-treated mice 3.90 ± 0.00 months) (*n* = 3 male mice and *n* = 1 female mouse) and *n* = 7 KUNV-treated mice 7.58 ± 0.56 months) (*n* = 1 male mouse and *n* = 6 female mice); *n* = 16 NL-G-F/E3 mice 8.26 ± 0.31 months) (*n* = 5 PBS-treated mice 7.94 ± 0.31 months) (*n* = 2 male mice and *n* = 3 female mice) and *n* = 11 KUNV-treated mice 8.41 ± 0.43 months) (*n* = 2 male mice and *n* = 9 female mice); *n* = 20 NL-G-F/E4 mice 8.29 ± 0.26 months) (*n* = 8 PBS-treated mice 9.08 ± 0.13 months) (*n* = 6 male mice and *n* = 2 female mice) and *n* = 12 KUNV-treated mice 7.77 ± 0.35 months) (*n* = 5 male mice and *n* = 7 female mice).

Analysis 2 included the following mice with temperature sensors, *n* = 143 mice 10.61 ± 0.33 months); *n* = 14 E3 mice 10.86 ± 0.88 months) (*n* = 3 PBS-treated mice 13.42 ± 0.64 months) (*n* = 3 female mice) and *n* = 11 KUNV-treated mice 10.16 ± 1.02 months) (*n* = 7 male mice and *n* = 4 female mice); *n* = 31 E4 mice 9.64 ± 0.97 months) (*n* = 13 PBS-treated mice 11.56 ± 1.26 months) (*n* = 8 male mice and *n* = 5 female mice) and *n* = 18 KUNV-treated mice 8.26 ± 1.33 months) (*n* = 11 male mice and *n* = 7 female mice); *n* = 22 NL-G-F/E2 mice 9.70 ± 0.83 months) (*n* = 9 PBS-treated mice 8.94 ± 1.61 months) (*n* = 7 male mice and *n* = 2 female mice) and *n* = 13 KUNV-treated mice 10.22 ± 0.89 months) (*n* = 5 male mice and *n* = 8 female mice); *n* = 27 NL-G-F/E3 mice 9.91 ± 0.54 months) (*n* = 10 PBS-treated mice 11.01 ± 1.25 months) (*n* = 2 male mice and *n* = 8 female mice) and *n* = 17 KUNV-treated mice 9.26 ± 0.42 months) (*n* = 5 male mice and *n* = 12 female mice); and *n* = 49 NL-G-F/E4 mice 11.93 ± 0.50 months) (*n* = 22 PBS-treated mice 12.32 ± 0.63 months) (*n* = 6 male mice and *n* = 16 female mice) and *n* = 27 KUNV-treated mice 11.62 ± 0.74 months) (*n* = 9 male mice and *n* = 18 female mice).

TS100 millimeter-scale (7.5 × 7.5 × 4.2 mm) CubiSens™ wireless sensors (CubeWorks, Ann Arbor, MI) were implanted in the abdomen for accurate, real-time temperature measurement, as described (40). These sensors allow measuring body temperature in group-housed mice. The sensors were implanted subcutaneously, and body temperatures were acquired and analyzed prior and during the behavioral testing, as described (40). Due to technical issues, most data were not saved for week 3 after KUNV or PBS infection and therefore the temperature data for this week were excluded from the analysis.

All the mice were group-housed, and mice in the same cage received the same treatment. Animals were maintained on a 1,200-h light/dark schedule (lights on at 06:00), with laboratory chow (PicoLab Rodent diet 20, # 5053; PMI Nutrition International, St. Louis, MO, USA) and water provided *ad libitum*. Behavioral testing occurred during the light cycle. All procedures complied with the National Institutes of Health Guide for the Care and Use of Laboratory Animals and with IACUC approval at Oregon Health & Sciences University. Experimenters were blinded to the genotype and treatment of the mice until all the data were analyzed.

#### 2.1.2 Study 2

To determine cortical insoluble and soluble p-tau levels 6 days following inoculation with KUNV(1,000 pfu, *i.p.*) or PBS, 93 mice (3.10 ± 0.11 month-old) were analyzed: E2 (*n* = 6 PBS-treated mice (3 mice/sex) and 7 KUNV-treated mice (3 males and 4 females), E3 (3 PBS-treated mice (2 males and 1 female), E3/E4 (*n* = 6 PBS-treated mice (3 mice/sex) and 7 KUNV-treated mice (3 males and 4 females), E4 (*n* = 6 PBS-treated mice (3 mice/sex), NL-G-F/E2 (*n* = 5 PBS-treated mice (2 males and 3 females) and 6 KUNV-treated mice (2 males and 4 females), NL-G-F/E3 (*n* = 5 PBS-treated mice (3 males and 2 females) and 6 KUNV-treated mice (3 mice/sex), NL-G-F/E3/E4 (*n* = 6 PBS-treated mice (3 mice/sex) and 6 KUNV-treated mice (3 mice/sex), and NL-G-F/E4 (*n* = 5 PBS-treated mice (2 males and 3 females) and 7 KUNV-treated mice (4 males and 3 females).

### 2.2 Treatments

All viral infections were with KUNV (obtained from BEI Resources), as reported previously (40). Mice were infected intraperitoneally (*i.p.*) with 1,000 focus-forming units (ffu) KUNV or PBS as indicated. The mice underwent behavioral testing, starting 30 days postinfection (dpi), as illustrated in **Figure 1** and in (40).

**Figure 1:**
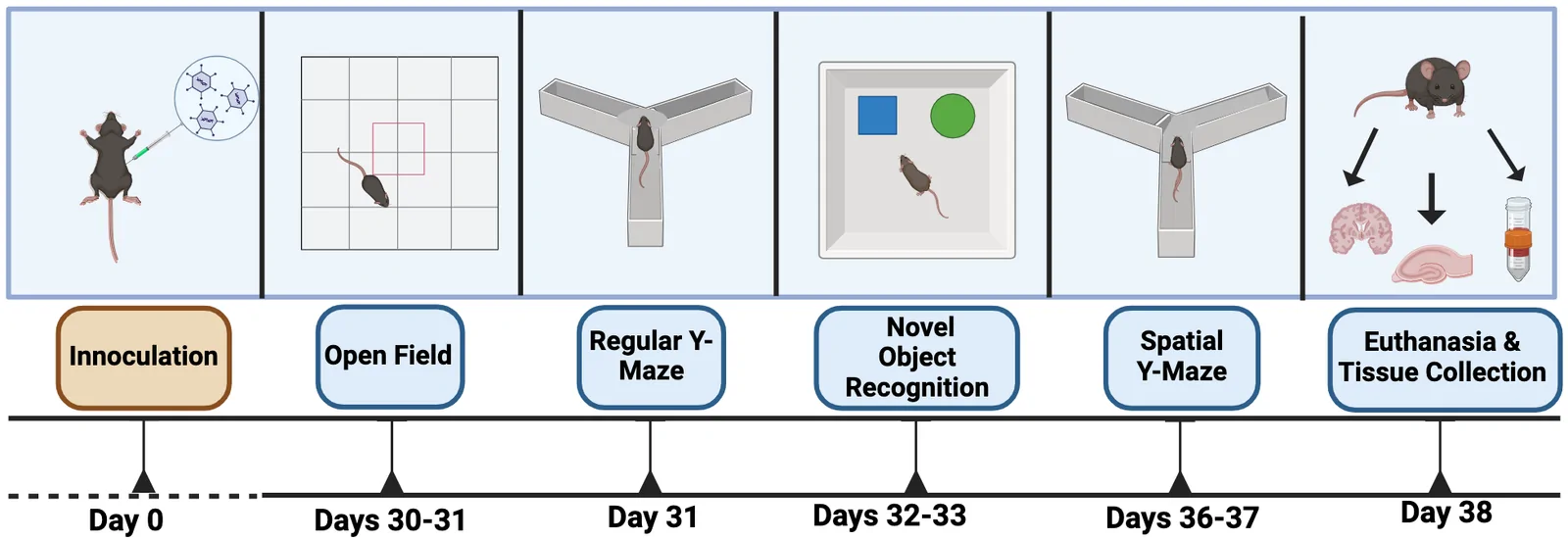
Schedule of behavioral testing. In the mornings of days 30 and 31, mice were tested for measures of activity, measures of anxiety, and spatial habituation in the open field. In the afternoon of day 31, mice were tested for spontaneous alternation in the regular Y maze. On days 32 and 33, the mice were tested for object recognition. On days 36 and 37, the mice were tested in the spatial Y maze. On day 38, the mice were euthanized by cervical dislocation, and blood was collected in EDTA-containing tubes and the hippocampus and cortex from each hemibrain were dissected.

### 2.3 Behavioral testing

Mice were behaviorally tested as illustrated in **Figure 1**.

#### 2.3.1 Open field and novel object recognition

The mice were put in an open field enclosure (16 × 16 inches, Kinder Scientific, Poway, CA) for 5 min on two subsequent days and on the two following days in the open field containing two objects for 10 min. On day 3, the two objects were identical, and on day 4, one object was replaced with a novel object. Between trial, the arenas and objects were cleaned with 0.5% acetic acid. Interaction within a 2-cm proximity with the object was coded as object exploration by hand scoring videos acquired with Noldus EthoVision software (version XT17, Wageningen, The Netherlands). A discrimination index, defined as the time spent exploring the familiar object, was subtracted from the time exploring the novel object, and the resulting number divided by the total time spent exploring both objects was analyzed. Other outcomes analyzed were distance moved and time spent in the center in the open field in the absence and presence of objects.

#### 2.3.2 Regular Y maze

Activity levels and hippocampus-dependent spontaneous alternations were assessed in the regular Y maze test (Harvard Apparatus, Panlab, Holliston, MA, United States) in a 5-min trial. The maze was cleaned with 0.5% acetic acid between trials. Performance was recorded using Noldus EthoVision software (version XT17) and hand-scored to analyze the number of arm entries (an activity measure) and the percent spontaneous alternation (a cognitive measure).

#### 2.3.3 Spatial Y maze

The spatial Y-maze test was conducted in the same enclosure as what was used for the regular Y maze test, but this task was conducted over 2 consecutive days. On day 1, one arm was blocked off. Mice were allowed to explore the maze for 5 min. On day 2, mice were allowed to explore the maze with all arms accessible for 5 min. Performance was recorded using EthoVision software (version XT17). Digital videos of day 2 were analyzed to determine the percent number of entries in the novel arm out of the entries in all three arms and the time spent in the novel arm (the arm that was blocked off during day 1). The criterion for an arm entry was when all four limbs were within the arm.

### 2.4 Cytokine mRNA expression

Following euthanasia by cervical dislocation, brains were dissected. RNA was isolated from cortical and hippocampal tissue of one hemisphere, extracted using TRIzol reagent (Invitrogen) according to the manufacturer’s protocol, and analyzed by qRT-PCR for expression of the inflammatory mediators. RNA was. Relative expression of the cytokines TNF-α, IFN-γ, and the T cell receptor α chain (as a marker of T cell infiltration into the brain) was determined by qRT-PCR using gene-specific primer-probe sets (Thermo Fisher) and normalized to β-actin mRNA expression using the ΔΔCt method (46). Viral genomes were quantified using WNV_KUNV_-specific primer probes. the primer/probe sequences for KUNV detection and quantitation are:

Forward primer 5′-AGTGGAGAAGTGGAGCGATGTT-3′

Reverse primer 5′-CAGGCTGCCACACCAAATG-3′

Probe FAM-CATACTCTGGCAAACGA-MGB

### 2.5 Aβ analyses

The cortex of the other hemisphere was processed for analyses of soluble and insoluble Aβ in the cortex using MyBioSource ELISA kits (catalog numbers MBS760432 and MBS268504, respectively; San Diego, MA, United States), according to the recommended guidelines in the production information sheets and as described (40). For the analyses of insoluble Aβ levels, the tissue samples were diluted 1: 4,000. Standard curves were generated with the same buffer dilution as the samples. The ELISAs were read at 450 nm using a SpectraMax iD5 Multi-Mode Microplate Reader (Molecular Devices, VWR 76175-474, San Jose, CA, United States). The standard curves were generated, and the levels in the samples were determined using GraphPad Prism software (San Diego, CA, United States). Total protein amounts in the samples were determined using a BCA protein assay kit (Pierce, Thermo Scientific, catalog #23225, Waltham, MA, United States), and the samples were read at 562 nm using an iD5 Reader (Molecular Devices, San Jose, CA).

### 2.6 p-tau analyses

The cortex of the other hemisphere was also analyzed for insoluble and soluble murine phosphorylated tau levels in the cortex using the Invitrogen mouse p-tau (pS199) ELISA (catalog number KMB7041); Thermo Fisher, Waltham, MA, United States). The insoluble and soluble fractions were generated as described for the Aβ analyses above. Standard curves were generated with the same buffer dilution as the samples. The ELISAs were read at 450 nm using a SpectraMax iD5 Multi-Mode Microplate Reader (Molecular Devices, VWR 76175-474, San Jose, CA, United States). The standard curves were generated and the levels in the samples determined using GraphPad Prism software (San Diego, CA, United States). Total protein amounts in the samples were determined using a BCA protein assay kit (Pierce, Thermo Scientific, catalog #23225, Waltham, MA, United States), and the samples were read at 450 nm using an iD5 Reader (Molecular Devices, San Jose, CA).

### 2.7 Statistical analyses

All behavioral data are reported as mean ± standard error of the mean and were analyzed using SPSS v.22 (IBM, Armonk, NY, USA) or GraphPad v.10.6.1 (La Jolla, CA, USA) software. For analyses 1 and 2, genotype and treatment were included as factors in analysis of variance (ANOVAs) and sex was used as a covariate. For analysis 2, age was used as a covariate as well. Genotypes and ages, and in cases that there were statistical interactions in sexes, were analyzed separately as well. Repeated-measures ANOVAs were used when appropriate. Two-tailed paired t tests were used to assess spatial habituation learning in each group in the open field. For the circadian body temperature data, the light and dark periods were analyzed as separate analyses, with the mean body temperature in the light or dark period of each day as the repeated measure. For analysis 1, we first analyzed all NL-G-F/apoE mice together. In addition, we compared circadian body temperatures in NL-G-F/apoE4 and E4 mice. For analyses 1 and 2, we first analyzed all NL-G-F/apoE mice together and analyzed the E3 and E4 mice together. In addition, we compared circadian body temperatures in NL-G-F/apoE4 and E4 mice and NL-G-F/E3 and E3 mice. Based on treatment × genotype interactions, we also analyzed each genotype separately. For study 2, NL-G-F genotype, apoE genotype treatment, and sex were used as factors. In case we suspected potential outliers for a given figure panel, we ran a Robust regression and outlier test (ROUT). In case outliers were identified, they were removed and noted in the results section.

Statistical significance was considered as *p* < 0.05. When sphericity was violated (Mauchly’s test), Greenhouse–Geisser corrections were used. Mice were tested in separate cohorts, each containing mice of all experimental groups. All researchers were blinded to genotype and treatment; the code was only broken after the data were analyzed.

For clarity, the effects of genotype, treatment, sex, and interactions for activity levels and time spent in the center of the open field are summarized in **Table 1**. For all other data, only effects of genotype and treatment are indicated in main text and figures. For details of the results of the statistical analyses related to effects of day, sex, and age, see the supplementary document.

**Table 1:** Activity levels and time spent in the center of the open field.

Open field activity levels	Genotype	Treatment	Sex	Interaction
NL-G-F/apoE mice; analysis 1			Females: Effect of day: (F(1,14) = 50.708, p < 0.001)	Day × sex interaction: (F(1,72) = 13.709, p < 0.001)
ApoE mice: analysis 1		(F(1,44) = 5.036, p = 0.030): KUNV < PBS	Effect of sex: (F(1,44) = 28.618, p < 0.001): females > males	Day × sex interaction (F(1,44) = 6.922, p = 0.012) Day × genotype interaction: (F(1,44) = 5.261, p = 0.027)
NL-G-F/apoE mice; analysis 2	(F(1,127) = 5.893, p = 0.004): NL-G-F/E3 > NL-G-F/E2; NL-G-F/E3 > NL-G-F/E4		(F(1,127) = 4.789, p = 0.030): females > males	day × sex interaction (F(1,127) = 16.756, p < 0.001)
NL-G-F/apoE males		In NL-G-F/E3 mice: KUNV > PBS In NL-G-F/E4 mice: KUNV < PBS		
NL-G-F/apoE females	(F(2,75) = 7.274, p = 0.001): NL-G-F/E3 > NL-G-F/E2; NL-G-F/E3 > NL-G-F/E4			
NL-G-F/E2 males		In NL-G-F/E2 males: (F(1,12) = 4.992, p = 0.045)		In males: day × treatment interaction (F(1,12) = 5.746, p = 0.034): a more profound effect of treatment on day 1 than day 2
Open field time in center				
NL-G-F/apoE mice: analysis 1	(F(2,73) = 5.418, p = 0.006): NL-G-F/E2 < NL-G-F/E3; NL-G-F/E2 < NL-G-F/E4			day × treatment interaction (F(1,73) = 4.092, p = 0.047): a more pronounced effect of treatment on day 1 than day 2; NL-G-F/E2 and NL-G-F/E4: KUNV < PBS
Open field time in center day 1 only	(F(2,73) = 3.602, p = 0.032): NL-G-F/E2 < NL-G-F/E4			
ApoE mice: analysis 1	(F(1,45) = 4.392, p = 0.042): E3 > E4			
NL-G-F/apoE mice: analysis 2	(F(2,126) = 13.769, p < 0.001): NL-G-F/E2 < NL-G-F/E3; NL-G-F/E2 < NL-G-F/E4			Day × treatment interaction (F(1,126) = 4.267, p = 0.041): a more pronounced Effect of treatment on day 1 than day 2; NL-G-F/E2 and NL-G-F/E4: KUNV < PBS
NL-G-F/E2		(F(1,31) = 14.331, p < 0.001): KUNV < PBS		
Open field time in center day 1 only		NL-G-F/E2: (F(1,31) = 6.473, p = 0.016): KUNV < PBS NL-G-F/E4 mice: (F(1,62) = 4.239, p = 0.044): KUNV < PBS		

Three principal components factor analyses (PCAs) were performed for analysis 2 to determine the relationships between performances on the various behavioral tasks, treatment, genotype, cortical soluble and insoluble Aβ40, Aβ42, and p-tau levels, and cortical immune measures, and mean body temperatures during the dark and light periods during weeks 1, 2, and 4 of individual mice. The first PCA included only NL-G-F/apoE mice. The second PCA included only apoE mice without NL-G-F. The third PCA involved all mice. Measures entered into the model were genotype, diet, distance moved in the open field on day 1 (activity measure), time spent in the center of the open field on day 1 (anxiety measure), difference in activity levels in the open field between days 1 and 2 divided by activity levels on day 1 (spatial habituation: cognitive measure), arm entries in the Y maze (activity measure), spontaneous alternation in the Y maze (cognitive measures), time spent (s) in the novel arm on the spatial Y maze, cortical levels of soluble and insoluble p-tau, Aβ40, and Aβ42 (only for the PCA with only NL-G-F/apoE mice), and cortical transcript levels of IFN-γ, TCR-α, and TNF-α levels. The PCAs were performed using SPSS, and factors with eigenvalue >1.0 were considered significant. The varimax rotated matrix was used to interpret the factor loadings.

## 3 Results

### 3.2 Open field and novel object recognition

We first assessed whether KUNV and genotype affect behavioral performance in the open field in the absence and presence of objects. The effects of genotype, treatment, sex, and interactions for activity levels and time spent in the center of the open field are summarized in **Table 1**.

#### 3.2.1 Activity levels in the open field

##### 3.2.1.1 Analysis 1

There were no effects of genotype or treatment in NL-G-F/apoE mice (**Figure 2**). When activity levels of E3 and E4 mice were analyzed in the open field, there was an effect of treatment (*F*(1,44) = 5.036, *p* = 0.030) (**Figures 3A, B**), with lower activity levels in KUNV- than PBS-treated mice.

**Figure 2:**
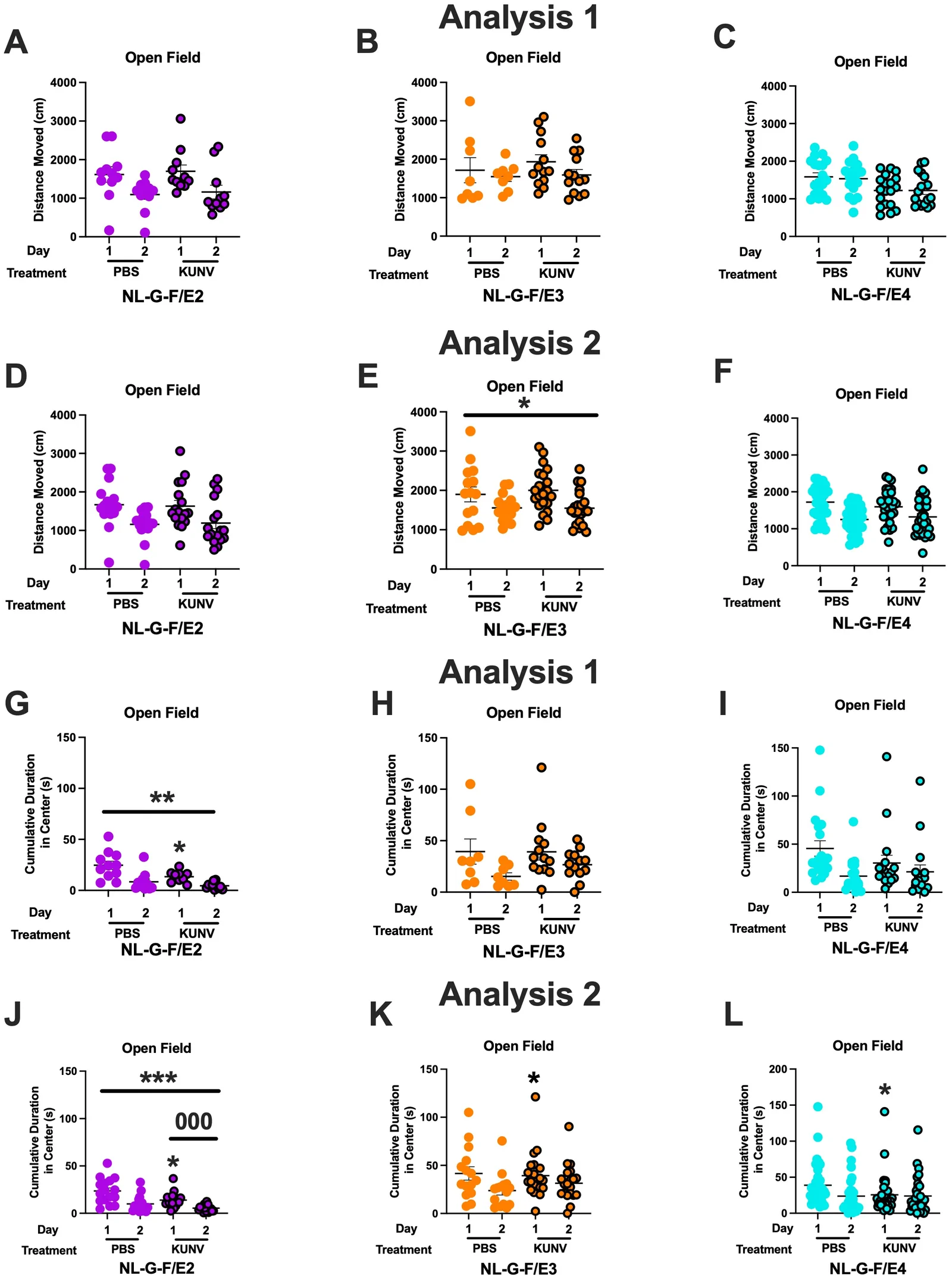
Activity levels **(A–F)** and measures of anxiety **(G–L)** in KUNV- and PBS-treated NL-G-F/E2 **(A, D, G, J)**, NL-G-F/E3 **(B, E, H, K)**, and NL-G-F/E4 **(C, F, I, L)** mice in the open field. **(D–F)**. In NL-G-F/apoE mice and analysis 2, there was an effect of genotype (*F*(1,127) = 5.893, ^0^*p* = 0.004); NL-G-F/E3 mice were more active than NL-F-F/E2 **(D)** (*p* = 0.004, Tukey’s) (mean: 1,413) and NL-G-F/E4 mice **(F)** (*p* = 0.006, Tukey’s) (mean: 1,473). **(G)** In NL-G-F/E2 mice and analysis 1, KUNV-treated mice spend less time in the center of the open field on day 2 than day 1. *t* = 2.193, *p = 0.0488. This was not seen in PBS-treated mice. **(G–I)**. There was an effect of genotype (*F*(2,73) = 3.602, *p* = 0.032); NL-G-F/E2 mice spent less time in the center of the open field than NL-G-F/E4 **(I)** mice. ^oo^*p* = 0.040, (Tukey’s) versus NL-G-F/E4 and there was a trend toward spending less time in the center than NL-G-F/E3 [**(K)**, *p* = 0.061, Tukey’s]. **(J–L)**. In NL-G-F/E2 mice **(J)**, NL-G-F/E3 **(K)**, and NL-G-F/E4 **(L)** mice and analysis 2 and on the first day of testing, there was an effect of treatment (*F*(1,31) = 6.473, *p = 0.016), with KUNV-treated mice spending less time in the center than PBS-treated mice. When only NL-G-F/E2 mice were analyzed, there also was an effect of treatment (*F*(1,31) = 14.331, ^000^*p* < 0.001), with KUNV-treated mice spending less time in the center than PBS-treated mice In addition, there was an effect of genotype (*F*(2,126) = 13.769, ^***^p < 0.001), with NL-G-F/E2 mice **(J)** spending less time in the center of the open field than NL-G-F/E3 **(K)** and NL-G-F/E4 **(L)** mice.

**Figure 3:**
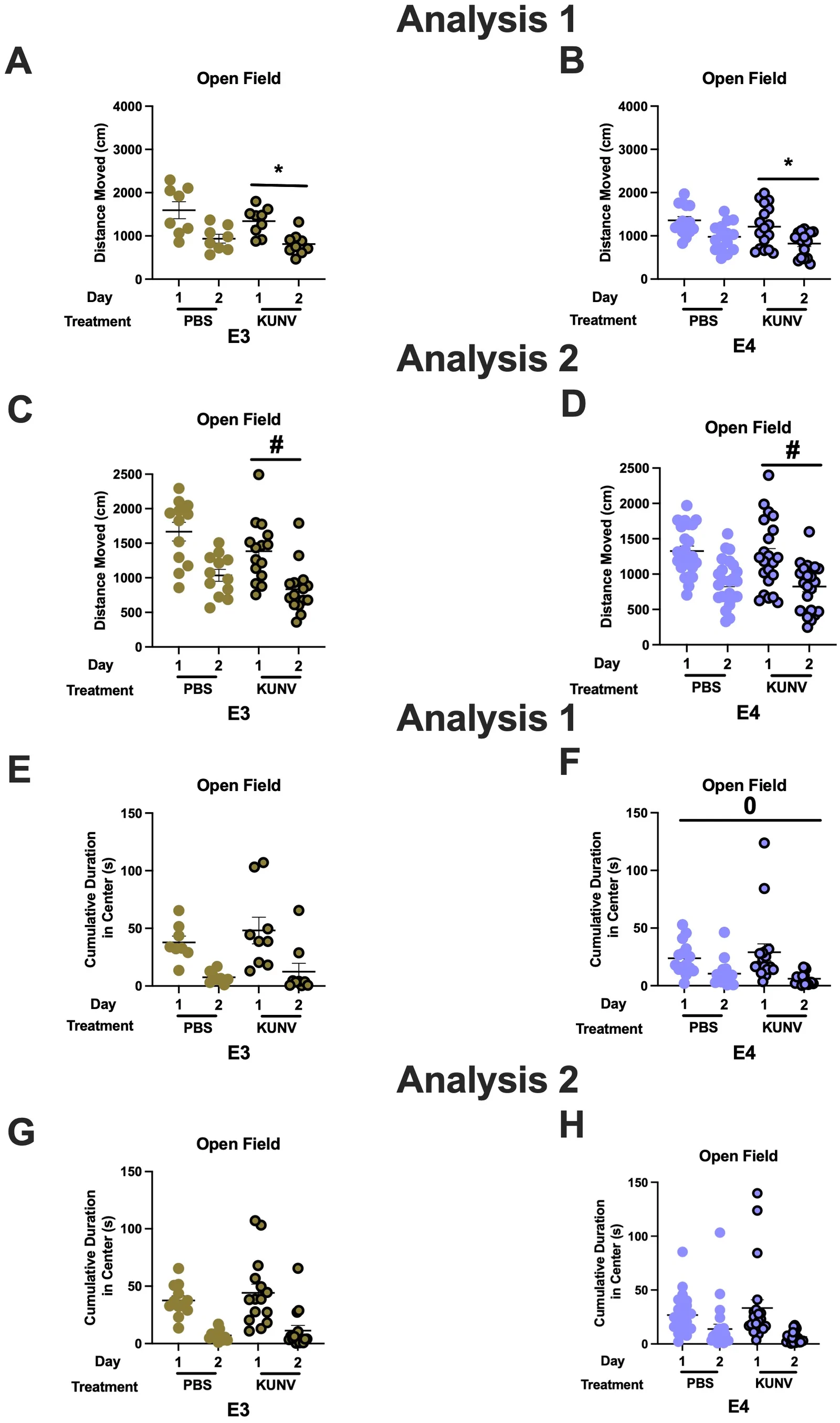
Activity levels and measures of anxiety in KUNV- and PBS-treated E3 **(A, C, E, G)** and E4 **(B, D, F, H)** mice in the open field. **(A, B)** In E3 and E4 mice and analysis 1, there was an effect of treatment (*F*(1,44) = 5.036, ^*^p = 0.030), with lower activity levels in KUNV- than PBS-treated mice. **(C, D)** In E3 and E4 mice and analysis 2, there was a trend toward an effect of treatment (*F*(1,67) = 3.132, ^#^*p* = 0.072), with a trend toward lower activity levels in KUNV- than PBS-treated mice. **(E, F)** In E3 and E4 mice and analysis 1, there was an effect of genotype (*F*(1,45) = 4.392, ^0^*p* = 0.042), with E3 mice spending more time in the center than E4 mice. **(G, H)**

##### 3.2.1.2 Analysis 2

When the activity levels of NL-G-F/E2, NL-G-F/E3, and NL-G-F/E4 mice in the open field were analyzed, there was an effect of genotype (*F*(2,127) = 5.893, *p* = 0.004), with NL-G-F/E3 mice (**Figure 2E**) (mean: 1,755) being more active than NL-G-F/E2 (**Figure 2D**) (*p* = 0.004, Tukey’s) (mean: 1,413) and NL-G-F/E4 mice (**Figure 2F**) (*p* = 0.006, Tukey’s) (mean: 1,473).

In NL-G-F/E3 males, there was an effect of treatment (*F*(1,12) = 4.992, *p* = 0.0453), with higher activity levels in KUNV- than PBS-treated mice, and a day × treatment interaction (*F*(1,12) = 5.746, *p* = 0.0337) (**Figure 4B**) (**Figure 4C**).

**Figure 4:**
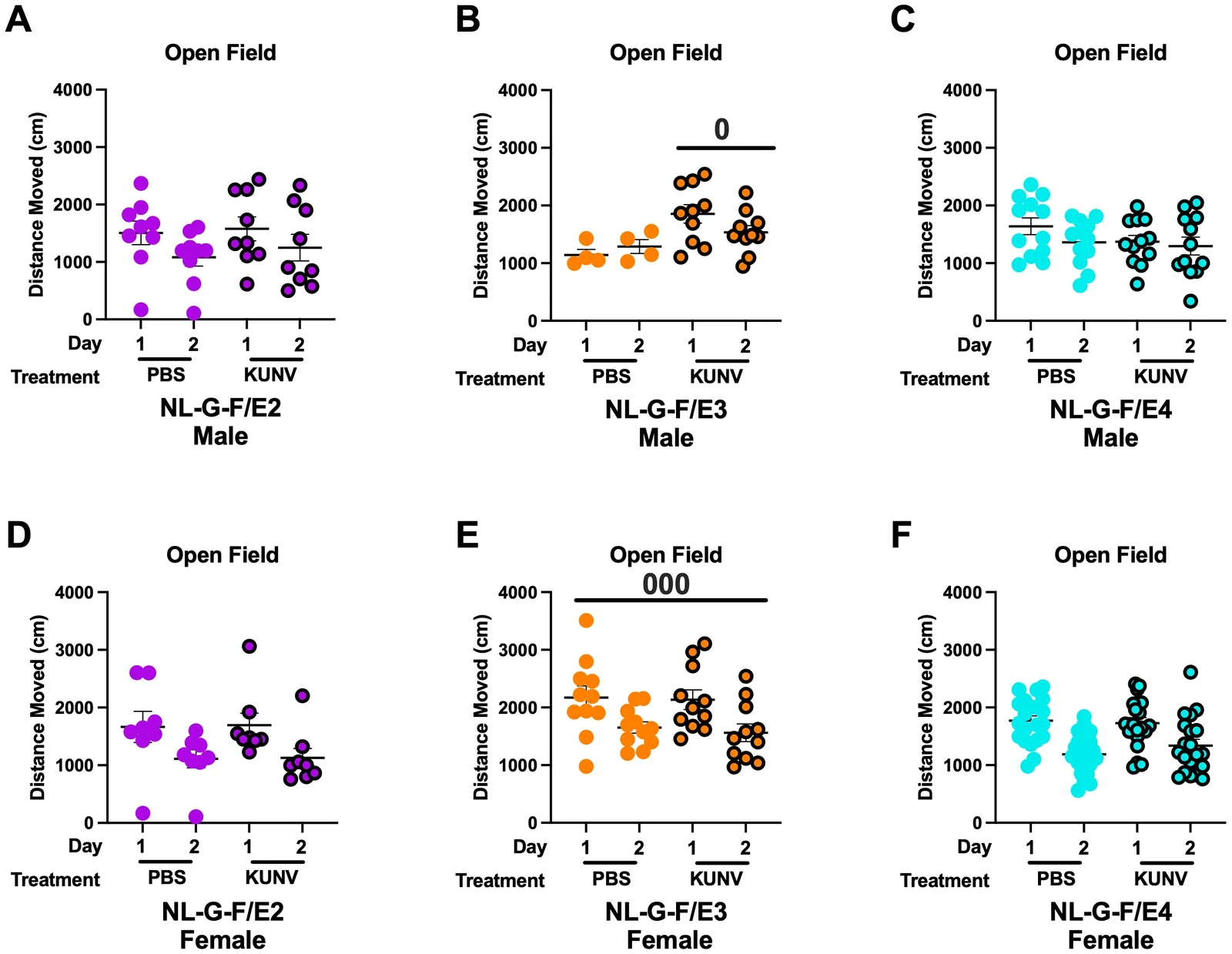
Activity levels in KUNV- and PBS-treated male **(A–C)** and female **(D–F)** NL-G-F/E2 **(A, D)**, NL-G-F/E3 **(B, E)**, and NL-G-F/E4 **(C, F)** mice in analysis 2 in the open field. **(B)** In NL-G-F/E3 male mice and analysis 2, there was an effect of treatment (*F*(1,12) = 4.992, ^0^*p* = 0.0453), with higher activity levels in KUNV- than PBS-treated mice. **(D–F)**. In NL-G-F/apoE female mice, there was an effect of genotype (*F*(2,75) = 7.274, ^000^*p* = 0.001), with NL-G-F/E3 mice being more active than NL-F-F/E2 (*p* = 0.011, Tukey’s) and NL-G-F/E4 mice (*p* = 0.002, Tukey’s).

In females (**Figures 4D–F**), there was an effect of genotype (*F*(2,75) = 7.274, *p* = 0.001), with NL-G-F/E3 mice (mean: 1,881) being more active than NL-G-F/E2 (*p* = 0.011, Tukey’s) (mean: 1,484) and NL-G-F/E4 mice (*p* = 0.002, Tukey’s) (mean: 1,506).

When the activity levels of NL-G-F/E3 and NL-G-F/E4 mice in the open field were analyzed, there was an effect of genotype (*F*(1,95) = 11.432, *p* = 0.001), a day × genotype × treatment interaction (*F*(1,95) = 4.012, *p* = 0.048).

In males, there was an effect of treatment (*F*(1,12) = 4.992, *p* = 0.045), and a day × treatment interaction (*F*(1,12) = 5.746, *p* = 0.034), with a more profound effect of treatment on day 1 than day 2; higher activity levels in KUNV- than PBS-treated NL-G-F/E3 mice, but lower activity levels in KUNV- than PBS-treated NL-G-F/E4 mice, with comparable activity levels in KUNV- and PBS-treated NL-G-F/E2 mice.

When activity levels of E3 and E4 mice in the open field were analyzed, there was a trend towards an effect of treatment (*F*(1,67) = 3.132, *p* = 0.072) (**Figures 2C, D**).

In E3 mice in females, there was a trend towards a day × treatment interaction (*F*(1,11) = 4.239, *p* = 0.064).

#### 3.2.2 Time spent in the center of the open field

##### 3.2.2.1 Analysis 1

In NL-G-F/E2, NL-G-F/E3, and NL-G-F/E4 mice (**Figures 2G, H**), there was an effect of genotype (*F*(2,73) = 5.418, *p* = 0.006), with NL-G-F/E2 mice (mean: 12.79 s) (**Figure 2G**) spending less time in the center of the open field than NL-G-F/E3 (mean: 30.80 s) (*p* = 0.011, Tukey’s) (**Figure 2H**) and NL-G-F/E4 mice (mean: 28.79 s) (*p* = 0.011, Tukey’s) (**Figure 2E**), and a day × treatment interaction (*F*(1,73) = 4.092, *p* = 0.047), with a more pronounced effect of treatment on day 1 than day 2; less time spent in the center of the open field in KUNV- than PBS-treated NL-G-F/E2 and NL-G-F/E4 mice, with comparable time spent in the center of KUNV- and PBS-treated NL-G-F/E3 mice.

When time spent in the center of the open field on day 1 only was analyzed, there was an effect of genotype (*F*(2,73) = 3.602, *p* = 0.032), with NL-G-F/E2 mice (mean: 19.06 s) (**Figure 2G**) spending less time in the center of the open field than NL-G-F/E4 mice (mean: 38.43 s) (*p* = 0.040, Tukey’s) (**Figure 2I**) and trending to spent less time in the center of the open field than NL-G-F/E3 mice (*p* = 0.061, Tukey’s) (**Figure 2H**).

In E3 and E4 mice, there was an effect of genotype (*F*(1,45) = 4.392, *p* = 0.042), with E3 mice spending more time in the center than E4 mice (**Figures 3E, F**), and a trend towards a day × genotype interaction (*F*(1,45) = 3.374, *p* = 0.073).

##### 3.2.2.2 Analysis 2

When time spent in the center of the open field in NL-G-F/E2, NL-G-F/E3, and NL-G-F/E4 mice was analyzed, there was an effect of genotype (*F*(2,126) = 13.769, *p* < 0.001), with NL-G-F/E2 mice (**Figures 2J**, mean: 13.08) spending less time in the center of the open field than NL-G-F/E3 (**Figure 2K**, mean 34.29) and NL-G-F/E4 (**Figures 2L**, mean: 28.09) mice, a day × age interaction (*F*(1,126) = 10.365, *p* = 0.002), and a day × treatment interaction (*F*(1,126) = 4.267, *p* = 0.041), with a more pronounced effect of treatment on day 1 than day 2; less time spent in the center of the open field in KUNV- than PBS-treated NL-G-F/E2 and NL-G-F/E4 mice, with comparable time spent in the center of KUNV- and PBS-treated NL-G-F/E3 mice.

When times spent in the center of the open field was analyzed, in NL-G-F/E2 mice, there was an effect of treatment (*F*(1,31) = 14.331, *p* < 0.001), with KUNV-treated mice spending less time in the center than PBS-treated mice (**Figure 2J**). In NL-G-F/E4 mice, there was a trend towards a day × treatment interaction (*F*(1,31) = 2.901, *p* = 0.094) (**Figure 2L**).

When time spent in the center of the open field on the first day of testing of NL-G-F/E2, NL-G-F/E3, and NL-G-F/E4 mice was analyzed, there was an effect of genotype (*F*(2,127) = 8.330, *p* < 0.001), with NL-G-F/E2 mice (mean: 18.58) (**Figure 2J**) spending less time in the center of the open field than NL-G-F/E3 (mean 40.28) (**Figure 2K**) and NL-G-F/E4 (mean: 32.36 s) (**Figure 2L**) mice, an effect of treatment (*F*(1,127) = 4.335, *p* = 0.039), with KUNV-treated mice spending less time in the center than PBS-treated mice, and an effect of sex (*F*(1,127) = 4.533, *p* = 0.035).

When time spent in the center of the open field on the first day of testing of NL-G-F/E3 and NL-G-F/E4 mice was analyzed (**Figures 2K, L**), there was a trend towards an effect of treatment (*F*(1,96) = 2.781, *p* = 0.099).

In NL-G-F/E2 mice, there was an effect of treatment (*F*(1,31) = 6.473, *p* = 0.016), with KUNV-treated mice spending less time in the center than PBS-treated mice. In NL-G-F/E3 mice, there were no significant effects. In NL-G-F/E4 mice, there was an effect of treatment (*F*(1,62) = 4.239, *p* = 0.044) and an effect of sex (*F*(1,62) = 6.569, *p* = 0.013), with KUNV-treated mice spending less time in the center than PBS-treated mice.

When time spent in the center of the open field of E3 and E4 mice was analyzed (**Figures 3G, H**), there was a trend towards a day × genotype interaction (*F*(1,67) = 3.844, *p* = 0.054).

When time spent in the center of the open field on the first day of testing of E3 and E4 mice was analyzed, there was a trend towards a day × genotype interaction (*F*(1,67) = 3.844, *p* = 0.054) (**Figures 3G, H**).

In NL-G-F/E3 males, there was an effect of treatment (*F*(1,12) = 4.992, *p* = 0.0453), with higher activity levels in KUNV- than PBS-treated mice, and a day × treatment interaction (*F*(1,12) = 5.746, *p* = 0.0337) (**Figure 4B**).

In females (**Figures 4D–F**), there was an effect of genotype (*F*(2,75) = 7.274, *p* = 0.001), with NL-G-F/E3 mice (mean: 1,881 cm) being more active than NL-F-F/E2 (*p* = 0.011, Tukey’s) (mean: 1,484 cm) and NL-G-F/E4 mice (*p* = 0.002, Tukey’s) (mean: 1,506 cm).

#### 3.2.3 Activity levels in the open field containing objects

##### 3.2.3.1 Analysis 1

When activity in the open field in the presence of the two objects of E3 and E4 mice was analyzed, there was a trend toward an effect of treatment (*F*(1,15) = 3.829, *p* = 0.069), with a trend to higher activity levels in PBS- than KUNV-treated mice (**Figures 5A, B**).

**Figure 5:**
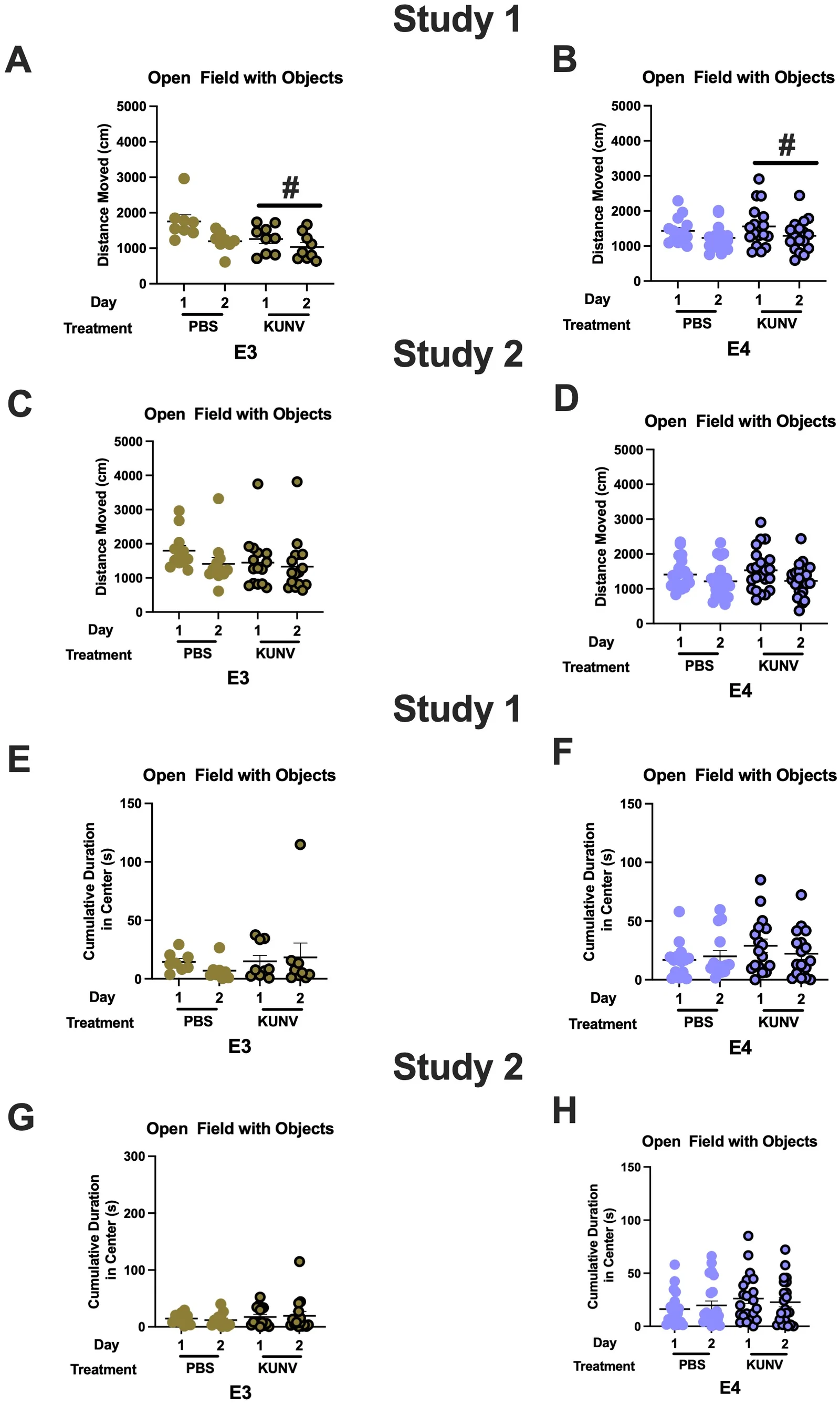
Activity levels **(A–D)** and time spent in the center **(E–H)** of the open field containing objects of KUNV- and PBS-treated E3 **(A, C, E, G)** and E4 **(B, D, F, H)** mice. **(A, B)** In E3 and E4 mice and in analysis 1, there was a trend towards an effect of treatment (*F*(1,15) = 3.829, ^#^*p* = 0.069), with a trend to higher activity levels in PBS- than KUNV-treated mice.

##### 3.2.3.2 Analysis 2

When activity in the open field in the presence of the two objects of NL-G-F/E2, NL-G-F/E3, and NL-G-F/E4 mice was analyzed, there was an effect of genotype (*F*(2,125) = 6.631, *p* < 0.002), with NL-G-F/E3 mice (mean: 2,617) (**Supplementary Figure S2E**) moving more than NL-G-F/E2 (mean: 1,963) (*p* = 0.002, Tukey’s) (**Supplementary Figure S2D**) and NL-G-F/E4 (2,178) (*p* = 0.02, Tukey’s) (**Supplementary Figure S2F**) mice, a day × genotype interaction (*F*(2,125) = 4.739, *p* = 0.010), and a trend toward a day × genotype × treatment interaction (*F*(2,125) = 2.415, *p* = 0.094).

In NL-G-F/E2 mice, there was a day × treatment interaction (*F*(1,31) = 4.768, *p* = 0.037), with lower activity levels in PBS-treated mice on day 2 than day 1 but comparable activity levels in KUNV-treated mice (**Supplementary Figure S2D**).

When activity in the open field in the presence of the two objects in E3 and E4 mice was analyzed, there was a day × genotype × treatment interaction (*F*(1,68) = 4.433, *p* = 0.039), with higher activity levels in on day 1 than day 2 in PBS- than KUNV-treated E3 mice; in contrast, in E4 mice the activity levels on both days showed in similar patterns in PBS- and KUNV-treated mice (**Figures 5C, D**).

#### 3.2.4 Time spent in the center of the open field containing objects

##### 3.2.4.1 Analysis 1

In NL-G-F/E2, NL-G-F/E3, and NL-G-F/E4 mice, there was an effect of genotype (*F*(2,72) = 5.171, *p* = 0.008), with NL-G-F/E2 mice (mean: 14.93) (**Supplementary Figure S2G**) spending less time in the center containing objects than NL-G-F/E3 (mean: 40.48 s) (*p* = 0.029, Tukey’s) (**Supplementary Figure S2H**) and NL-G-F/E4 mice) (mean: 42.0.1 s) (*p* = 0.007, Tukey’s) (**Supplementary Figure S2I**) and a trend toward a day × genotype × treatment interaction (*F*(2,72) = 2.958, *p* = 0.058).

In NL-G-F/E4 mice, there was a day × treatment interaction (*F*(1,33) = 4.969, *p* = 0.033) (**Supplementary Figure S2I**), with lower activity levels on day 2 than day 1 in KUNV-treated mice but comparable activity levels on both days in PBS-treated mice.

In E3 and E4 mice, there was a trend toward a day × genotype × treatment interaction (*F*(1,43) = 3.420, *p* = 0.071) (**Figures 5E, F**), with a trend toward PBS-treated E3 and KUNV-treated E4 mice spending less time in the center of the open field containing objects on day 2 than day 1, whereas KUNV-treated E3 mice and PBS-treated E4 mice spent comparable times in the center of the open field containing objects on both days.

##### 3.2.4.2 Analysis 2

When time spent in the center of the open field in the presence of the two objects of NL-G-F/E2, NL-G-F/E3, and NL-G-F/E4 mice was analyzed (**Supplementary Figures S2J-S2L**), there was an effect of genotype (*F*(2,125) = 6.868, *p* = 0.001), with NL-G-F/E2 mice (mean: 27.84 s) (**Supplementary Figure S2J**) spending less time in the center of the open field containing objects than NL-G-F/E4 mice (68.44 s) (*p* < 0.001, Tukey’s) (**Supplementary Figure S2L**), an effect of age (*F*(1,125) = 33.350, *p* < 0.001), and a trend towards a day × genotype interaction (*F*(2,125) = 2.886, *p* = 0.060).

In NL-G-F/apoE female mice, there was an effect of treatment (*F*(1,12) = 8.028, *p* = 0.015), with KUNV-treated mice (mean day 1: 8.6 s; mean day 2: 7.8 s) spending less time in the center than PBS-treated mice (mean day 1: 24.3 s; mean day 2: 13.5 s). For E3 and E4 mice, there was no genotype or treatment effect for time spent in the center of the open field in the presence of objects (**Figures 5G, H**).

#### 3.2.5 Time spent exploring objects on the object recognition test day

##### 3.2.5.1 Analysis 1

When time spent exploring the objects on the object recognition test day in NL-G-F/E2, NL-G-F/E3, and NL-G-F/E4 mice was analyzed (**Supplementary Figures S3A–C**), there was an effect of genotype (*F*(2,73) = 9.496, *p* < 0.001), with NL-G-F/E2 mice (mean: 6.3 s) spending less time exploring objects than NL-G-F/E3 (mean: 21.1 s) and NL-G-F/E4 (mean: 25.2 s) mice.

In E3 and E4, there were no significant effects (**Supplementary Figures S4A, B**).

##### 3.2.5.2 Analysis 2

When time spent exploring the objects on the object recognition test day in NL-G-F/E2, NL-G-F/E3, and NL-G-F/E4 mice was analyzed, there was an effect of genotype (*F*(2,126) = 10.954, *p* < 0.001), with NL-G-F/E2 mice (mean: 12.31) (**Supplementary Figure S3D**) spending less time exploring the objects than NL-G-F/E3 (27.16) (p = 0.008, Tukey’s) (**Supplementary Figure S3E**) and NL-G-F/E4 mice (32.91) (p < 0.001, Tukey’s) (**Supplementary Figure S3F**).

In E3 and E4 mice, there was an effect of genotype (*F*(2,73) = 9.496, *p* < 0.001), with E3 mice spending less time exploring the objects than E4 mice (S4C, S4D).

#### 3.2.6 Discrimination index

##### 3.2.6.1 Analysis 1

When the discrimination index in the object recognition test in NL-G-F/E2, NL-G-F/E3, and NL-G-F/E4 mice was analyzed, there were no significant effects (**Supplementary Figures S3G–H**).

When the discrimination index in young E3 and E4 mice was analyzed, there was no genotype or treatment effect (**Supplementary Figures S3E, F**).

##### 3.2.6.2 Analysis 2

When the discrimination index in the object recognition test in NL-G-F/E2, NL-G-F/E3, and NL-G-F/E4 mice was analyzed, there were no significant effects (**Supplementary Figures S3G-I**).

When the discrimination index in the object recognition test in E3 and E4 mice was analyzed, there were no significant genotype or treatment effects (**Supplementary Figure S4**).

### 3.5 Regular Y maze

We next assessed whether KUNV and genotype affect spontaneous alternations and activity levels in the regular Y maze.

#### 3.5.1 Arm entries in the regular Y maze

##### 3.5.1.1 Analysis 1

There was no effect of genotype or treatment in NL-G-F/E2 (Figure 6A) or NL-G-F/E3 (**Figure 6B**) mice. In NL-G-F/E4 mice, there was a trend towards an effect of treatment (*F*(1,33) = 3.509, *p* = 0.070) (**Figure 6C**), with a trend towards higher activity levels in KUNV- than PBS-treated mice.

**Figure 6:**
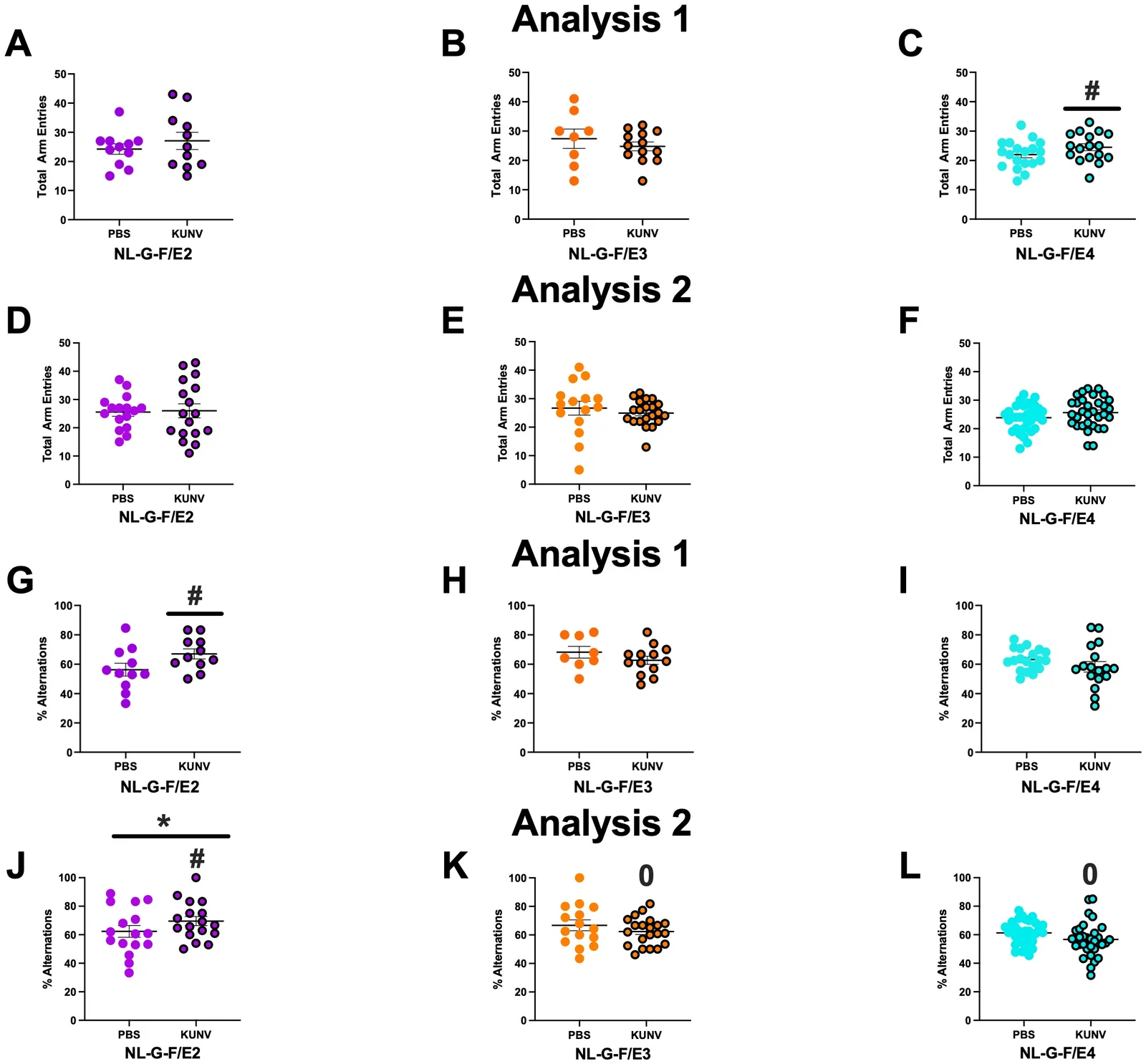
Arm entries **(A–F)** and percent spontaneous alternations **(G–L)** of KUNV- and PBS-treated NL-G-F/E2 **(A, D, G, J)**, NL-G-F/E3 **(B, E, H, L)**, and NL-G-F/E4 **(C, F, I, L)** mice. **(C)** In NL-G-F/E2 mice, there was a trend towards an effect of treatment with a trend towards higher activity levels in (*F*(1,19) = 3.937, ^#^*p* = 0.062). **(J)**. In NL-G-F/E2 mice and in analysis 2, there was a trend towards an effect of treatment with a trend towards a higher percentage of spontaneous alternation in KUNV- than PBS-treated mice (*F*(1,63) = 3.390, ^#^*p* = 0.070). **(J–L)**. There was an effect of genotype (*F*(2,126) = 4.316, *p = 0.015), with NL-G-F/E2 mice showing more spontaneous alternation than NL-G-F/E4 mice [*p* = 0.02, Tukey’s)] **(L)**. **(K, L)**. In NL-G-F/E3 and NL-G-F/E4 mice and in analysis 2, there was an effect of treatment with less spontaneous alternations in KUNV- than PBS-treated mice (*F*(1,95) = 4.775, ^0^*p* = 0.031).

##### 3.5.1.2 Analysis 2

No genotype or treatment effects were seen in analysis 2.

#### 3.5.2 Spontaneous alternation in the regular Y maze

##### 3.5.2.1 Analysis 1

In NL-G-F/E2, NL-G-F/E3, and NL-G-F/E4 mice (**Figures 6G–I**), there was a genotype × treatment interaction (*F*(2,72) = 3.684, *p* = 0.030). In NL-G-F/E2 mice (**Figure 6G**), there was a trend towards an effect of treatment (*F*(1,19) = 3.937, *p* = 0.062). There was no effect of genotype or treatment for arm entries.

In E3 and E4 mice, there was an effect of treatment (*F*(1,45) = 10.995, *p* = 0.002) (**Figures 7E, F**). There was no effect of genotype or treatment for arm entries (**Figures 7A–D**).

**Figure 7:**
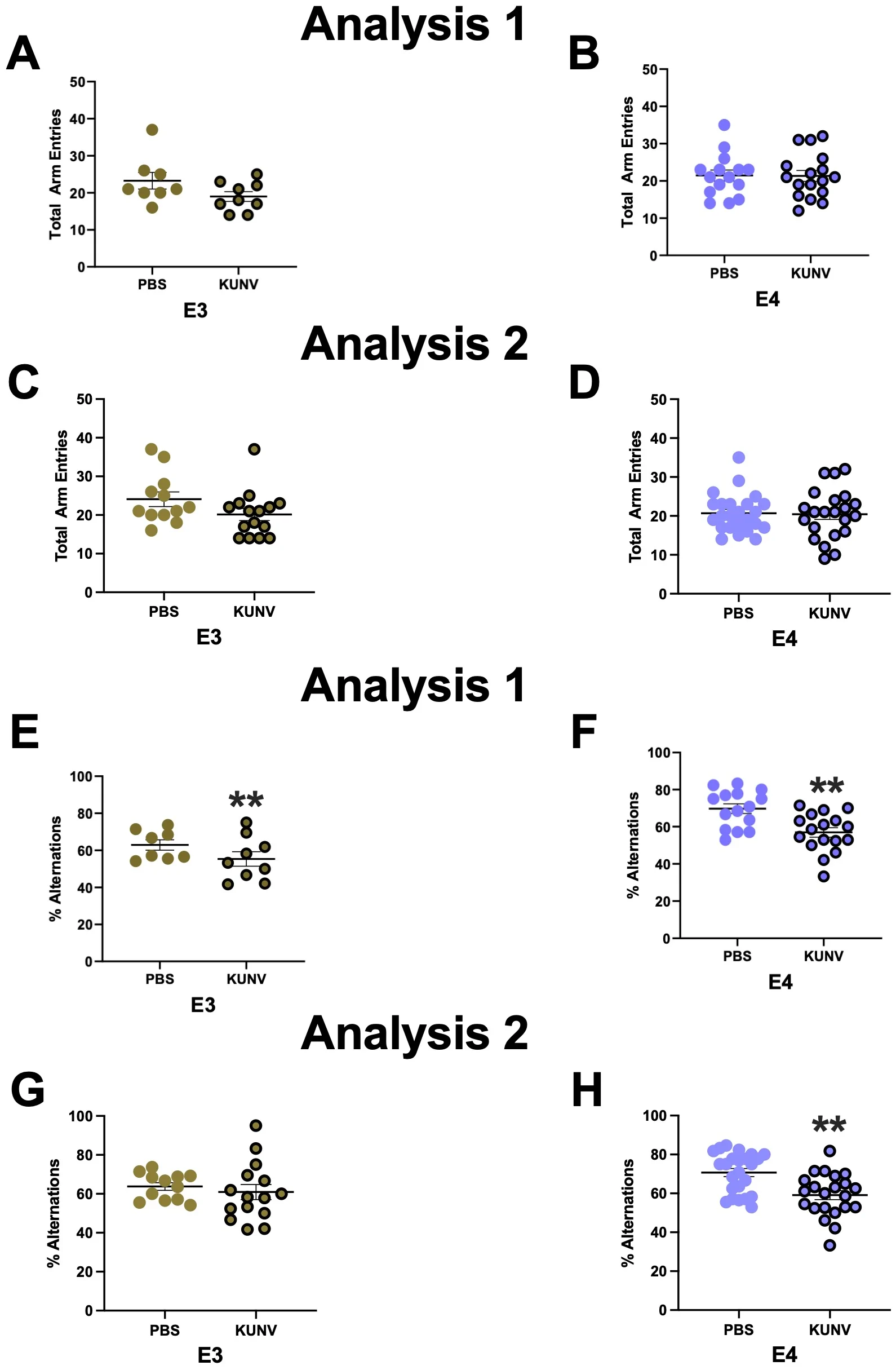
Arm entries **(A–D)** and percent spontaneous alternations **(E–H)** of KUNV- and PBS-treated E3 **(A, C, E, G)**, and E4 **(B, D, F, H)** mice. **(E, F)** In E3 and E4 mice and in analysis 1, there was an effect of treatment with less spontaneous alternation in KUNV- than PBS-treated mice (*F*(1,45) = 10.995, **p = 0.002). **(H)** In E4 mice and in analysis 2, there was an effect of treatment with less spontaneous alternation in KUNV- than PBS-treated mice (*F*(1,42) = 11.409, **p = 0.002).

##### 3.5.2.2 Analysis 2

There was no effect of genotype or treatment for arm entries in NL-G-F/E2 *Figure 6D), NL-G-F/E3 (Figure 6E), and N-L-G-F/E4 (**Figure 6F**) mice. When percent spontaneous alternation in NL-G-F/E2, NL-G-F/E3, and NL-G-F/E4 mice (**Figures 6J–L**), there was an effect of genotype (*F*(2,126) = 4.316, *p* = 0.015), with NL-G-F/E2 mice (66.07%) (**Figure 6J**) showing more spontaneous alternation than NL-G-F/E4 (mean: 59.02) (p = 0.02, Tukey’s)) (**Figure 6L**). There was also a trend towards a genotype × treatment interaction (*F*(2,126) = 3.064, *p* = 0.050).

When percent spontaneous alternation in NL-G-F/E3 and NL-G-F/E4 mice was analyzed, there was an effect of genotype (*F*(1,95) = 6.055, *p* = 0.016) and an effect of treatment (*F*(1,95) = 4.775, *p* = 0.031) (**Figures 6K, L**).

In NL-G-F/E4 mice, there was a trend towards an effect of treatment (*F*(1,63) = 3.390, *p* = 0.070).

When percent spontaneous alternation in E3 and E4 mice was analyzed, there was an effect of treatment (*F*(1,67) = 6.536, *p* = 0.013) (**Figures 7G, H**).

In E4 mice, there was an effect of treatment (*F*(1,42) = 11.409, *p* = 0.002) (**Figure 7H**).

### 3.6 Spatial Y maze

Subsequently, we assessed whether KUNV and genotype affect cognitive performance in the spatial Y maze.

#### 3.6.1 Time spent in the novel arm in the spatial Y maze

##### 3.6.1.1 Analysis 1

In NL-G-F/E2, NL-G-F/E3 and NL-G-F/E4 mice, there were no significant effects (**Supplementary Figures S5A–S5C**).

In E3 and E4 mice, there was an effect of treatment (*F*(1,44) = 4.116, *p =* 0.049) (**Figure 8A**), with KUNV-treated mice spending more time in the novel arm than PBS-treated mice.

**Figure 8:**
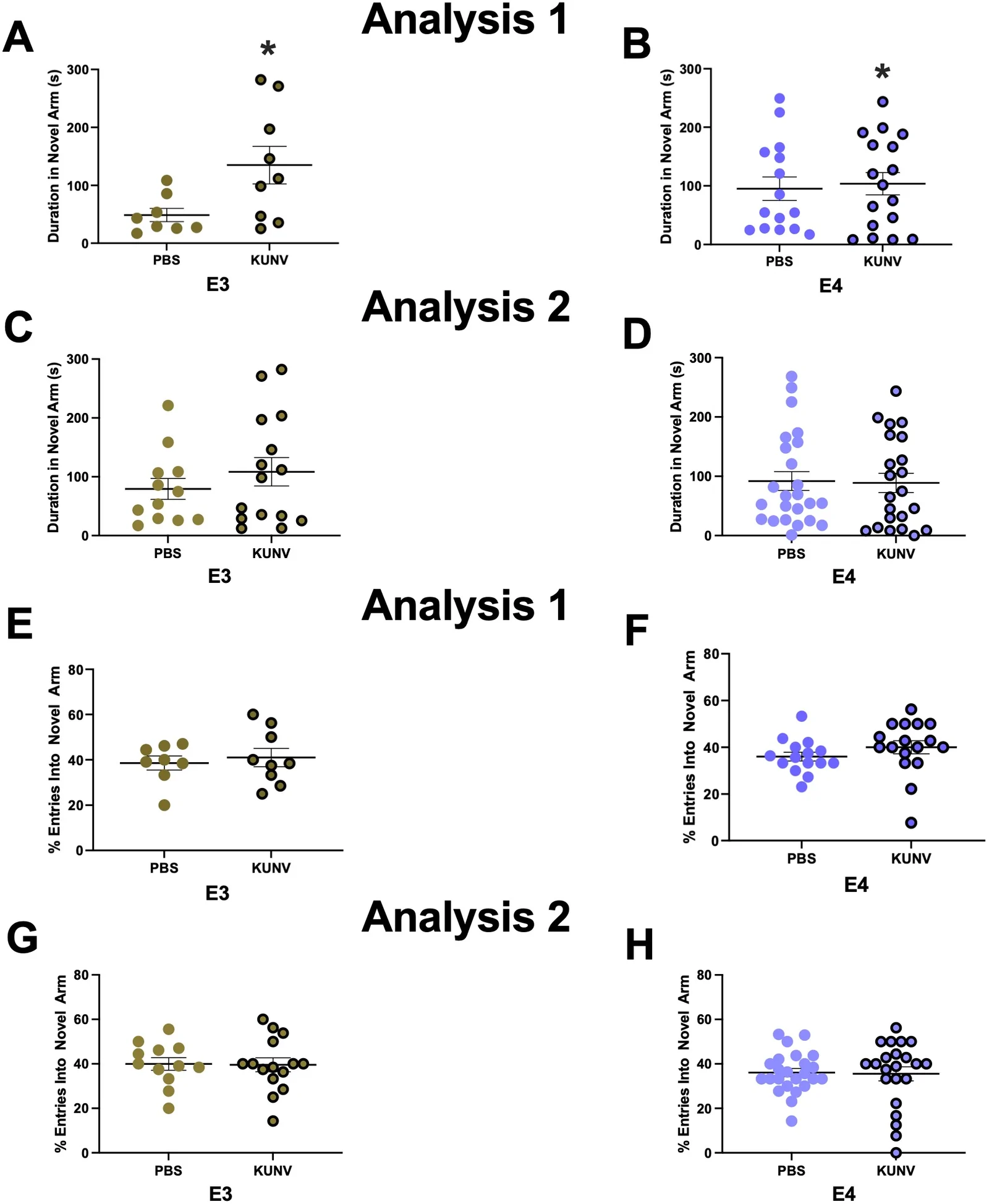
Duration **(A–D)** and percent entries **(E–H)** in the novel arm of the spatial Y maze of KUNV- and PBS-treated E3 **(A, C, E, G)** and E4 **(B, D, F, H)** mice. **(A, B)** In E3 and E4 mice and in analysis 1, there was an effect of treatment (*F*(1,44) = 4.116, *p = 0.049), with KUNV-treated mice spending more time in the novel arm than PBS-treated mice. **(C, D)**.

##### 3.6.1.2 Analysis 2

When time spent in the novel arm of NL-G-F/E2, NL-G-F/E3, and NL-G-F/E4 mice was analyzed, there was no effect of genotype or treatment (**Supplementary Figures S5D–F**).

When time spent in the novel arm of E3 and E4 mice was analyzed, KUNV-treated E3 and E4 mice spent more time in the novel arm than genotype-matched PBS-treated mice (**Figures 8A, B**).

#### 3.6.2 Percent entries in the novel arm in the spatial Y maze

##### 3.6.2.1 Analysis 1

When the percent entries in the novel arm of NL-G-F/E2, NL-G-F/E3, and NL-G-F/E4 mice was analyzed in NL-G-F/E2, NL-G-F/E3, and NL-G-F/E4 mice, there were no significant effects (**Supplementary Figures S5A–C**).

In E3 and E4 mice, there were no significant effects either (**Figures 8C, D**). There were no effects of genotype and treatment for percent entries into the novel arm (**Figures 8E–H**).

##### 3.6.2.2 Analysis 2

When the percent entries in the novel arm of NL-G-F/E2, NL-G-F/E3, and NL-G-F/E4 mice was analyzed, there was a trend towards an effect of sex (*F*(1,126) = 3.034, *p =* 0.084), and a trend towards a genotype × treatment interaction (*F*(1,126) = 2.355, *p =* 0.099), with a trend towards a higher percent entries in the novel arm in KUNV- than PBS-treated NL-G-F/E2 and NL-G-F/E3 mice; this pattern was not seen in NL-G-F/E4 mice (**Supplementary Figure S5G–I**).

In older NL-G-F/E3 and NL-G-F/E4 mice, there was a trend towards an effect of genotype (*F*(1,40) = 3.593, *p =* 0.065).

### 3.7 Circadian body temperatures

We also assessed whether KUNV and genotype affect circadian body temperatures.

#### 3.7.1 Analysis 1

##### 3.7.1.1 Week 1

In NL-G-F/apoE mice during the dark periods, there was a treatment × genotype interaction (*F*(2,39) = 4.309, *p =* 0.020). In NL-G-F/apoE mice during the light periods, there was a trend towards a treatment × genotype interaction (**Figures 9A, C, E**).

**Figure 9:**
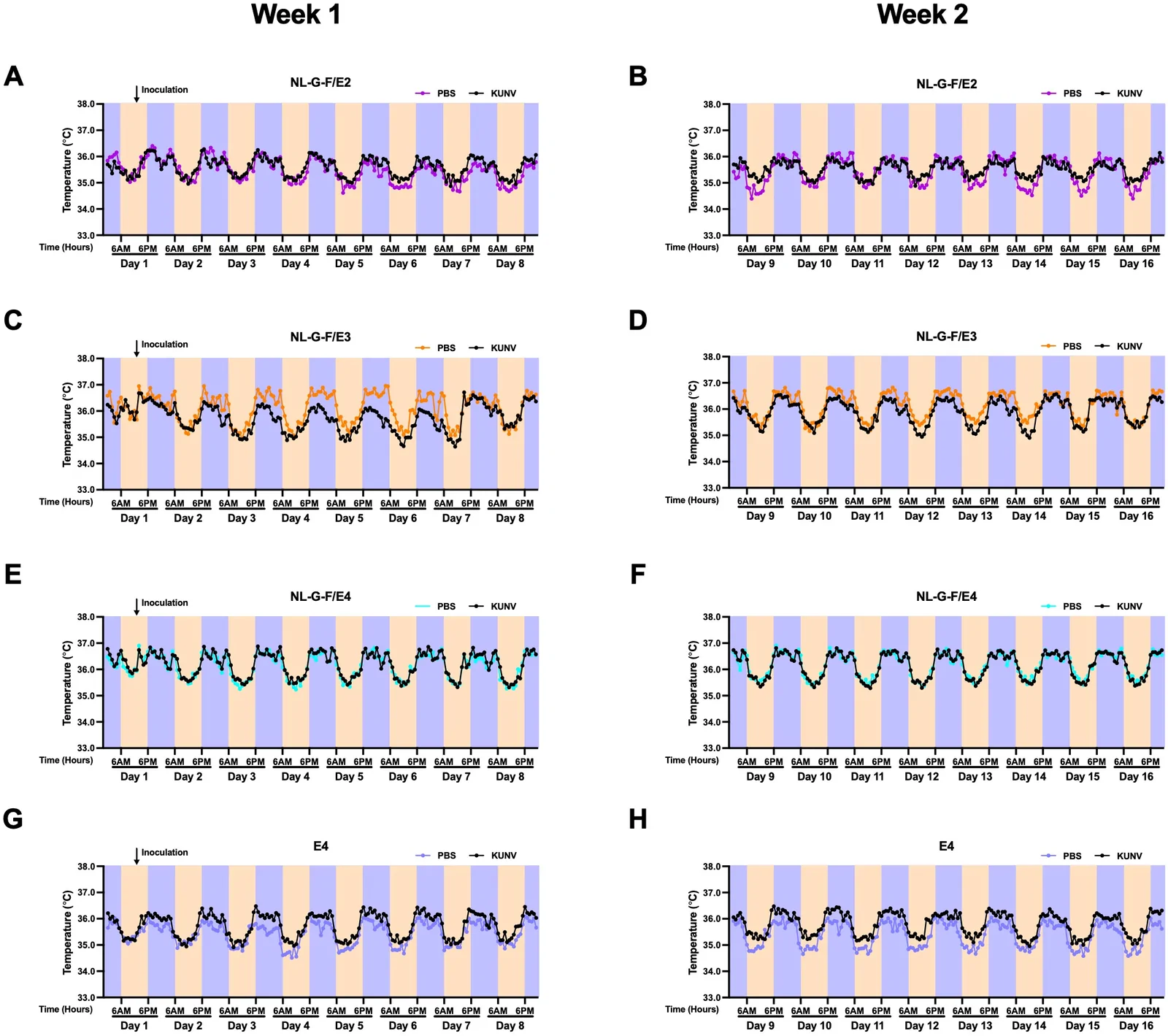
Circadian body temperatures of KUNV- and PBS-treated NL-G/E2 **(A, B)**, NL-G-F/E3 **(C, D)**, NL-G-F/E4 **(E, F)** and E4 **(G, H)** in the first **(A, C, E, G)** and second **(B, D, F, H)** weeks following treatment.

In NL-G-F/E2 mice during the dark periods, there were no significant effects (**Figure 9A**). In NL-G-F/E2 mice during the light periods, there was an effect of treatment (*F*(1,8) = 5.400, *p =* 0.049), with higher body temperatures in KUNV- than PBS-treated mice (**Figure 9A**). This effect was most pronounced during days 6-8.

In NL-G-F/E3 mice during the dark periods, there was an effect of treatment (*F*(1,14) = 9.460, *p =* 0.008), with lower body temperatures in KUNV- than PBS-treated mice (**Figure 9C**). This effect was most pronounced during nights 3-6. During the light periods, there was a trend towards an effect of treatment (*F*(1,14) = 4.500, *p =* 0.052), with a trend towards lower body temperature in KUN- than PBS-treated mice (**Figure 9C**). This trend was most pronounced during days 3-6.

##### 3.7.1.2 Week 2

In NL-G-F/apoE mice during the dark periods, there was an effect of genotype (*F*(2,38) = 3.397, *p =* 0.044), with a trend towards body temperatures in NL-G-F/E2 (35.97°C) being lower than those in NL-G-F/E3 (36.33°C) (*p* = 0.060, Tukey) (**Figures 9B, D, F**). In NL-G-F/apoE mice during the light periods, there was a trend towards a day × genotype interaction (*F*(8.871,168.542) = 1.807, *p =* 0.071, Greenhous–Geisser correction) (**Figures 9B, D, F**).

In NL-G-F/E2 mice during the dark periods, there were no significant effects (**Figure 9B**). In NL-G-F/E2 mice during the light periods, there was an effect of treatment (*F*(1,8) = 12.620, *p =* 0.007), with higher body temperatures in KUNV- than PBS-treated mice (**Figure 9B**). This effect was most pronounced on day 9 and days 12-16. In addition, there was a trend towards a day × treatment interaction (*F*(7,56) = 1.968, *p =* 0.076) (**Figure 9B**).

In NL-G-F/E3 mice during the light periods, there was a trend towards a day × treatment interaction (*F*(2.263,27.154) = 2.715, *p =* 0.078, Greenhous–Geisser correction) (**Figure 9D**).

When the body temperatures during the dark periods in NL-G-F/E4 and E4 mice were compared, there were no significant effects (**Figures 9E–H**). When the body temperatures during the light periods in NL-G-F/E4 and E4 mice were compared, there was a day by NL-G-F interaction (*F*(3.852,115.573) = 8.763, *p <* 0.001, Greenhous–Geisser correction). However, on no day was there a genotype difference in body temperatures (**Supplementary Table S1**).

##### 3.7.1.3 Week 4 (S6)

In NL-G-F/E4 mice during the dark and light periods, there were no effects of treatment.

In E4 mice during the light periods, there was a day × treatment interaction (*F*(1.872,22.469) = 5.327, *p =* 0.014, Greenhouse–Geisser). There was a trend towards an effect of treatment on day 2 (*p* = 0.082, two-tailed t test), with a trend towards a higher body temperature in KUNV (35.49 ± 0.17°C)- than PBS (34.86 ± 0.34°C)-treated mice.

When body temperatures in NL-G-F/E4 and E4 mice during the dark periods were compared, there was a night × NL-G-F interaction (*F*(2.306,48.417) = 3.265, *p =* 0.040, Greenhouse–Geisser), but no effect of NL-G-F in any night, and a trend towards a night × treatment interaction (*F*(2.306,48.417) = 2.760, *p =* 0.066, Greenhouse–Geisser). When body temperatures in NL-G-F/E4 and E4 mice during the light periods were compared, there was a day × treatment interaction (*F*(2.307,48.450) = 3.423, *p =* 0.035, Greenhouse–Geisser), with no effect of treatment on any day, a day × treatment × NL-G-F interaction (*F*(2.307,48.450) = 3.420, *p =* 0.035, Greenhouse–Geisser). There was a more profound treatment effect in E4 than NL-G-F/E4 mice, and this was seen on days 25 and 26 (**Supplementary Figure S6**). There was also a trend towards a day × NL-G-F interaction (*F*(2.307,48.450) = 2.405, *p =* 0.093, Greenhouse–Geisser).

#### 3.7.2 Analysis 2

##### 3.7.2.1 Week 1

In NL-G-F/apoE mice during the dark periods, there was an effect of genotype (*F*(2,88) = 10.289, *p <* 0.001), with body temperatures in NL-G-F/E2 (35.84°C) (**Figure 9A**) being lower than those in NL-G-F/E3 (36.20°C) (*p* = 0.043, Tukey) (**Figure 9C**) and NL-G-F/E4 mice (36.52°C) (*p* < 0.001, Tukey) (**Figure 9E**), those in NL-G-F/E3 being lower than those in NL-G-F/E4 (*p* = 0.024, Tukey), sex (*F*(1,88) = 29.539, *p <* 0.001), with higher body temperatures in females than males (**Supplementary Table S2**), age (*F*(1,88) = 4.420, *p =* 0.038), a treatment × genotype interaction (*F*(2,88) = 3.203, *p =* 0.045), and a night × genotype interaction (*F*(3.119,44.421) = 2.956, *p =* 0.007, Greenhouse–Geisser). In NL-G-F/apoE mice during the light periods, there was an effect of genotype (*F*(2,88) = 13.077, *p <* 0.001), with NL-G-F/E2 (35.24°C) being lower than those in NL-G-F/E3 (35.54°C) (*p* = 0.029, Tukey) and NL-G-F/E4 (35.81°C) (*p* < 0.001, Tukey) and NL-G-F/E3 being lower than those NL-G-F/E4 (*p* = 0.017, Tukey), a day × genotype interaction (*F*(7.289,320.733) = 6.246, *p <* 0.001, Greenhouse–Geisser), and day × treatment × genotype interaction (*F*(7.289,320.733) = 2.221, *p =* 0.030, Greenhouse–Geisser).

In NL-G-F/E3 mice during the dark periods, there was an effect of treatment (*F*(1,24) = 11.843, *p =* 0.002), with lower body temperatures in KUNV- than PBS-treated mice (**Figure 10C**). In NL-G-F/E3 mice during the light periods, there was an effect of treatment (*F*(1,24) = 3.310, *p =* 0.024), with higher body temperatures in KUNV- than PBS-treated mice.

**Figure 10:**
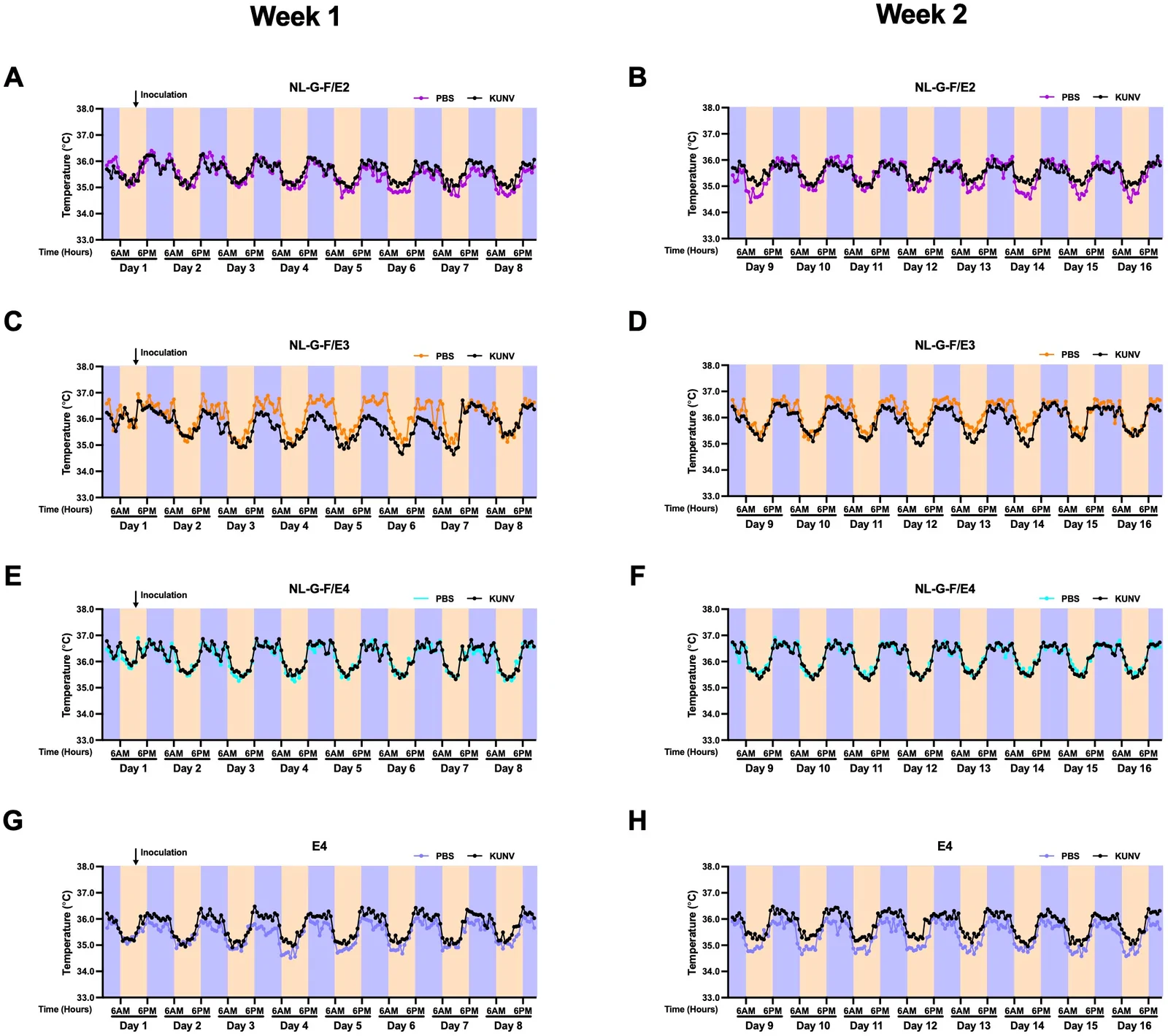
Circadian body temperatures of KUNV- and PBS-treated NL-G/E2 **(A, B)**, NL-G-F/E3 **(C, D)**, NL-G-F/E4 **(E, F)**, and E4 **(G, H)** mice in the first **(A, C, E, G, I)** and second **(B, D, F, H, J)** weeks following treatment.

In E4 mice during the dark periods, there was an effect of treatment (*F*(1,26) = 8.073, *p =* 0.009), with higher body temperatures in KUNV- than PBS-treated mice. In E4 mice during the light periods, there was a trend towards an effect of treatment (*F*(1,26) = 3.241, *p =* 0.083).

When the body temperatures in the dark periods in the NL-G-F/E4 and E4 mice were compared, there was an effect of treatment (*F*(1,72) = 5.429, *p =* 0.023), sex (*F*(1,72) = 60.969, *p <* 0.001), NL-G-F (*F*(1,72) = 16.419, *p =* 0.023), and a night × NL-G-F interaction (*F*(4.267,307.199) = 2.375, *p =* 0.048, Greenhouse–Geisser), with higher body temperatures in NL-G-F/E4 than E4 mice on all days (**Supplementary Table S10**). When the body temperatures in the light periods in the NL-G-F/E4 and E4 mice were compared, there was an effect of treatment (*F*(1,72) = 4.455, *p =* 0.038), NL-G-F (*F*(1,72) = 22.968, *p <* 0.001), a day × sex interaction (*F*(4.440,319.696) = 4.771, *p <* 0.001, Greenhouse–Geisser), and a day × NL-G-F interaction (*F*(4.440,319.696) = 9.377, *p <* 0.001, Greenhouse–Geisser), with higher body temperatures in NL-G-F/E4 than E4 mice on all days (**Supplementary Table S12**).

##### 3.7.2.2 Week 2

In NL-G-F/apoE mice during the dark periods, there was an effect of genotype (*F*(2,86) = 11.624, *p <* 0.001), with body temperatures in NL-G-F/E2 mice (35.81°C) (**Figure 10B**) being lower than those in NL-G-F/E3 (36.41°C) (*p* < 0.001, Tukey) (**Figure 10D**) and NL-G-F/E4 (36.57°C) mice (*p* < 0.001, Tukey) (**Figure 10F**), a night × treatment interaction (*F*(4.574,393.384) = 2.434, *p =* 0.039, Greenhouse–Geisser), but there was no effect of treatment in any night (**Supplementary Table S14**), a night × treatment × genotype interaction (*F*(9.184,393.384) = 2.353, *p =* 0.013, Greenhouse–Geisser. In NL-G-F/apoE mice during the light periods, there was an effect of genotype treatment (*F*(2,86) = 6.466, *p =* 0.002), with body temperatures in NL-G-F/E2 mice (35.20°C) being lower than those in NL-G-F/E3 (35.61°C) (*p* = 0.18, Tukey) and NL-G-F/E4 mice (35.75°C) (*p* < 0.001, Tukey), a day × treatment × genotype interaction (*F*(4.241,364.722) = 2.489, *p =* 0.011, Greenhouse–Geisser), and a trend towards a day × treatment interaction (*F*(4.241,364.722) = 1.977, *p =* 0.093, Greenhouse–Geisser).

In NL-G-F/E2 mice during the dark periods, there was an effect of treatment (*F*(1,18) = 6.403, *p =* 0.021), with higher body temperatures in KUNV- than PBS-treated mice. In NL-G-F/E2 mice during the light periods, there was a day × treatment interaction (*F*(1,17) = 2.273, *p =* 0.033), but no effect of treatment on any day (**Supplementary Table S17**).

In NL-G-F/E3 mice during the dark periods, there was a night × treatment interaction (*F*(7,147) = 2.589, *p =* 0.015), with lower body temperatures in KUNV- than PBS-treated mice on days 5 and 6 and a trend towards lower body temperatures in KUNV- than PBS-treated mice on days 2-4 (**Supplementary Table S20**). In NL-G-F/E3 mice during the light periods, there was a day × treatment interaction (*F*(3.702,81.441) = 3.742, *p =* 0.009, Greenhouse–Geisser), with lower body temperatures in KUNV- than PBS-treated mice on days 4 and 6 and a trend towards lower body temperatures in KUNV- than PBS-treated mice on day 5 (**Supplementary Table S21**).

In NL-G-F/E4 mice during the dark and light periods, there were no effects of treatment.

In E4 mice during the dark periods, there was an effect of treatment (*F*(1,25) = 4.719, *p =* 0.040), with lower body temperatures in KUNV- than PBS-treated mice. In E4 mice during the light periods, there was an effect of treatment (*F*(1,24) = 5.659, *p =* 0.026), with higher body temperatures in KUNV- than PBS-treated mice.

When body temperatures during the dark periods in NL-G-F/E4 and E4 mice were compared, there was an effect of treatment (*F*(1,72) = 5.204, *p =* 0.025), NL-G-F (*F*(1,71) = 11.698, *p =* 0.001), sex (*F*(1,71) = 62.117, *p <* 0.001). When body temperatures during the light periods in NL-G-F/E4 and E4 mice were compared, there was an effect of treatment (*F*(1,71) = 5.791, *p =* 0.019), NL-G-F (*F*(1,71) = 8.740, *p =* 0.004), a treatment × NL-G-F interaction (*F*(1,71) = 5.475, *p =* 0.022), and a day × NL-G-F interaction (*F*(3.411,244.342) = 3.026, *p =* 0.024, Greenhouse–Geisser).

##### 3.7.2.3 Week 3

In NL-G-F/E2 mice during the dark periods, there was a trend towards an effect of sex (*F*(1,7) = 5.313, *p* = 0.055) (**Supplementary Figures S7A**). In NL-G-F/E2 mice during the light periods, there was an effect of sex (*F*(1,7) = 13.556, *p* = 0.008), with higher body temperatures in females (35.29 ± 0.10°C) than males (33.94 ± 0.11°C), and a trend towards a day × age interaction (*F*(7,49) = 2.206, *p* = 0.050).

In E4 mice during the dark periods, there was an effect of treatment (*F*(1,24) = 6.061, *p* = 0.021), with higher body temperatures in KUNV- (35.50 ± 0.08°C) than PBS- (34.66 ± 0.17°C)-treated mice, an effect of sex (*F*(1,24) = 20.113, *p* < 0.001), with higher body temperatures in females (35.81 ± 0.10°C) than males (34.66 ± 0.11°C), and an effect of age (*F*(1,24) = 4.284, *p* = 0.049), with higher body temperatures in younger (35.15 ± 0.11°C) than older (34.66 ± 0.13°C) mice (**Supplementary Figures S7B**). In E4 mice during the light periods, there was an effect of sex (*F*(1,24) = 42.373, *p* < 0.001), with higher body temperatures in females (35.23 ± 0.09°C) than males (34.13 ± 0.09°C), a trend towards an effect of treatment (*F*(1,24) = 3.894, *p* = 0.060), a trend towards a day × sex interaction (*F*(2.082,49.961) = 3.098, *p =* 0.052, Greenhouse–Geisser), and a trend towards a day × age interaction (*F*(2.082,49.961) = 2.409, *p =* 0.098, Greenhouse–Geisser).

##### 3.7.2.4 Week 4

In NL-G-F/apoE mice during the dark periods, there was an effect of genotype (*F*(1,30) = 3.355, *p* = 0.048), with NL-G-F/E2 being different from NL-G-F/E4 (*p* = 0.037, Tukey’s), with lower body temperatures in NL-G-F/E2 mice being lower than those in NL-G-F/E4 mice, and with a trend of NL-G-F/E2 being different from NL-G-F/E3 (*p* = 0.062, Tukey’s), and a trend towards a genotype × treatment interaction (*F*(1,30) = 2.951, *p* = 0.080). In NL-G-F/apoE mice during the light periods, there was a trend towards an effect of genotype (*F*(2,29) = 3.397, *p* = 0.076) and a trend towards an effect of treatment (*F*(1,28) = 3.397, *p* = 0.076) (**Supplementary Figure S8**).

In NL-G-F/E2 mice during the dark periods, there was a day × treatment interaction (*F*(2.499,17.490) = 3.847, *p =* 0.034, Greenhouse–Geisser). In NL-G-F/E2 mice during the light periods, there was a day × treatment interaction.

In NL-G-F/E3 mice during the dark periods, there were no significant effects. In NL-G-F/E3 mice during the light periods, there were no significant effects.

In NL-G-F/E4 mice during the dark and light periods, there were no treatment effects.

In E4 mice during the dark period, there was an effect of treatment (*F*(1,23) = 7.917, *p* = 0.010), with higher body temperatures in KUNV- (35.35 ± 0.09°C) than PBS- (34.75 ± 0.15°C)-treated mice. In E4 mice during the light periods, there was a day × treatment interaction (*F*(2.539,58.391) = 4.961, *p =* 0.006, Greenhouse–Geisser), with higher body temperatures in KUNV- than PBS-treated mice on days 1 (PBS: 34.82 ± 0.15°C; KUNV: 35.40 ± 0.14°C; *p* = 0.009, two-tailed *t test*), 2 (PBS: 34.75 ± 0.16°C; KUNV: 35.36 ± 0.15°C; *p* = 0.009, two-tailed *t test*), and 8 (PBS: 34.81 ± 0.18°C; KUNV: 35.25 ± 0.08°C; *p* = 0.045, two-tailed *t test*) and a trend V a higher body temperature in KUNV- than PBS-treated mice on day 7 (PBS: 35.08 ± 0.16°C; KUNV: 35.38 ± 0.08°C; *p* = 0.097, two-tailed *t test*), and a trend towards an effect of treatment (*F*(1,23) = 3.532, *p* = 0.073).

Comparing body temperatures in NL-G-F/E4 and E4 mice during the dark periods, there was a NL-G-F × treatment interaction (*F*(1,37) = 4.770, *p* = 0.035), with more profound treatment effects in E4 than NL-G-F/E4 mice, and a night × NL-G-F interaction (*F*(2.834,104.850) = 3.389, *p =* 0.023, Greenhouse–Geisser), with lower body temperatures in NL-G-F/E4 than E4 mice in all nights (day 1: E4: 36.29 ± 0.13°C; NL-G-F/E4: 35.80 ± 0.10°C; *p* = 0.004, two-tailed *t test*; day 2: E4: 36.18 ± 0.10°C; NL-G-F/E4: 35.83 ± 0.11°C; *p* = 0.041, two-tailed *t test*; day 3: E4: 36.20 ± 0.11°C; NL-G-F/E4: 35.79 ± 0.11°C; *p* = 0.023, two-tailed *t test*; day 4: E4: 36.61 ± 0.11°C; NL-G-F/E4: 35.83 ± 0.10°C; *p* < 0.001, two-tailed *t test*; day 5: E4: 36.53 ± 0.10°C; NL-G-F/E4: 35.80 ± 0.10°C; *p* < 0.001, two-tailed *t test*; day 6: E4: 36.49 ± 0.10°C; NL-G-F/E4: 35.85 ± 0.10°C; *p* < 0.001, two-tailed *t test*; day 7: E4: 36.27 ± 0.08°C; NL-G-F/E4: 35.74 ± 0.09°C; *p* < 0.001, two-tailed *t test*; day 8: E4: 36.24 ± 0.08°C; NL-G-F/E4: 35.82 ± 0.11°C; *p* = 0.003, two-tailed *t test*). Comparing body temperatures in NL-G-F/E4 and E4 mice during the light periods, there was an effect of NL-G-F (*F*(1,37) = 9.746, *p* = 0.003), a day × NL-G-F × treatment interaction (*F*(3.114,115.8205) = 2.849, *p =* 0.039, Greenhouse–Geisser), with more profound treatment effects in E4 than NL-G-F/E4 mice on days 25 and 26, a trend towards an effect of age (*F*(1,37) = 2.915, *p* = 0.096) and a trend towards a day × treatment interaction (*F*(3.114,115.8205) = 2.511, *p =* 0.060, Greenhouse–Geisser).

### 3.8 Insoluble and soluble cortical Aβ40, Aβ42, the Aβ42/40 ratio, insoluble and soluble p-tau, and the insoluble/soluble p-tau ratio

Following behavioral testing, we assessed whether KUNV and genotype affect cortical Aβ and p-tau pathology.

The cortical tissues of the young mice (analysis 1) were processed for analysis of markers of AD pathology. In NL-G-F/apoE mice, there was an effect of genotype for insoluble cortical Aβ40 levels (*F*(2,56) = 6.588, *p* < 0.001) (**Figure 11A**). Insoluble cortical Aβ40 levels were higher in NL-G-F/E3 than NL-G-F/E2 (*p* < 0.001, Tukey’s) and NL-G-F/E4 mice (*p* = 0.007, Tukey’s).

**Figure 11:**
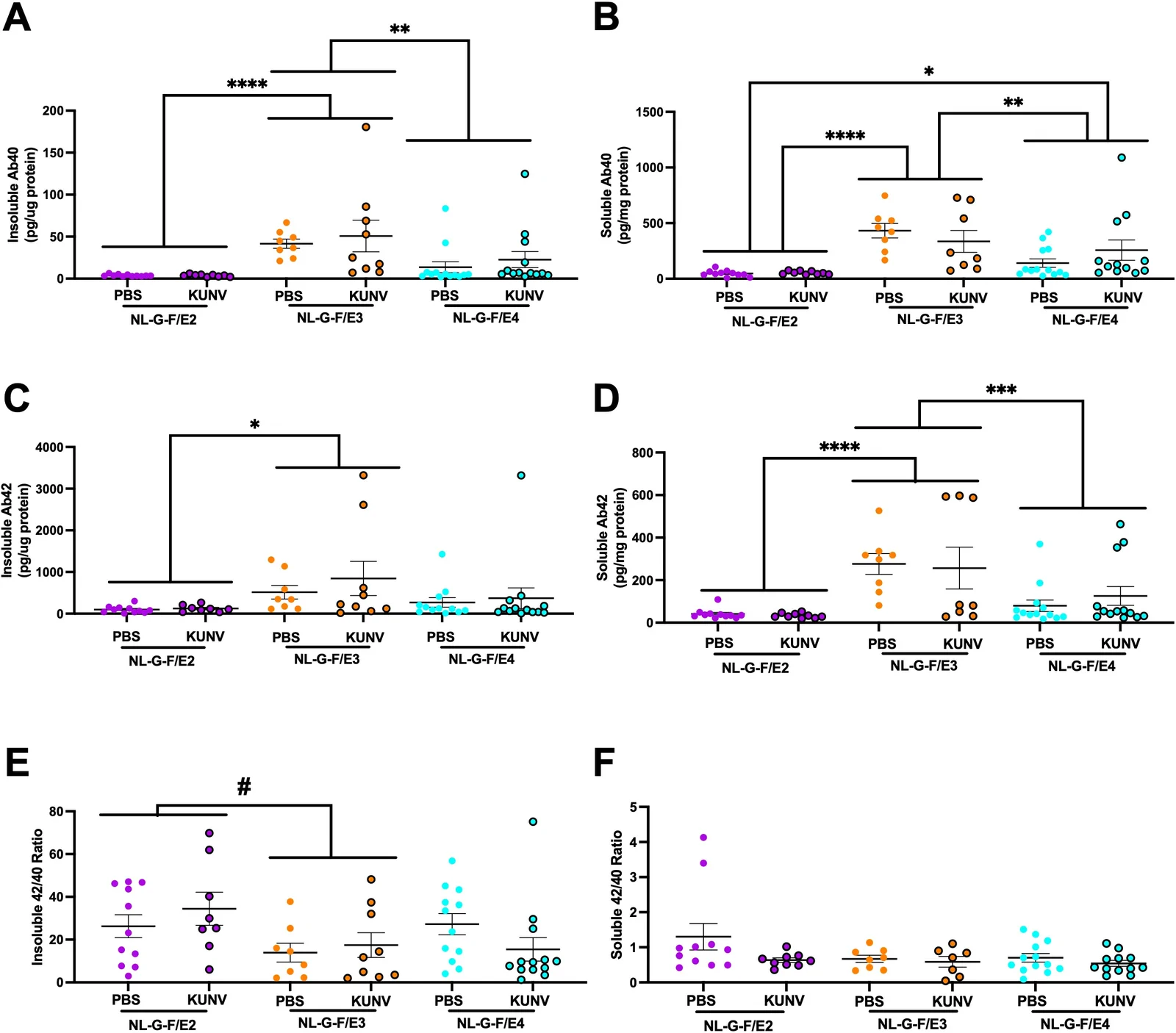
Insoluble **(A, C, E)** and soluble **(B, D, F)** Aβ40 **(A, B)** and Aβ42 **(C, D)** and the insoluble and soluble Aβ 42/40 ratio **(E, F)** in the cortex of KUNV- and PBS-treated NL-G-F/E2, NL-G-F/E3, and NL-G-F/E4 mice. **(A)** In NL-G-F/apoE mice, there was an effect of genotype for insoluble cortical Aβ40 levels (*F*(2,56) = 6.588, *p* < 0.001). Insoluble cortical Aβ40 levels were higher in NL-G-F/E3 than NL-G-F/E2 (****p < 0.001, Tukey’s) and NL-G-F/E4 mice (**p = 0.007, Tukey’s). **(B)** There was an effect of genotype (*F*(2,54) = 13.763, *p* < 0.001) for soluble cortical Aβ40 levels. Soluble cortical Aβ40 levels were higher in NL-G-F/E3 than NL-G-F/E2 (****p < 0.001, Tukey’s) and NL-G-F/E4 mice (**p = 0.007, Tukey’s) and higher in NL-G-F/E4 than NL-G-F/E2 mice (*p = 0.007, Tukey’s). **(C)** There was an effect of genotype on insoluble cortical Aβ42 levels (*F*(2,55) = 3.186, *p* = 0.049). Insoluble cortical Aβ42 levels were higher in NL-G-F/E3 than NL-G-F/E2 mice (*p = 0.034, Tukey’s). **(D)** For soluble cortical Aβ42 levels, there was an effect of genotype (*F*(2,56) = 12.369, *p* < 0.001). Insoluble cortical Aβ42 levels were higher in NL-G-F/E3 than NL-G-F/E2 (****p < 0.001, Tukey’s) and NL-G-F/E4 mice (***p = 0.002, Tukey’s). **(E)** For the insoluble Aβ42/40 ratio, there was a trend towards an effect of genotype (*F*(2,55) = 3.011, *p* = 0.057), with a trend towards a higher insoluble Aβ42/40 ratio in NL-G-F/E2 than NL-G-F/E3 mice (#*p* = 0.063, Tukey’s). **(F)** For the soluble Aβ42/40 ratio, there were no effects of genotype or treatment.

There was an effect of genotype (*F*(2,54) = 13.763, *p* < 0.001) for soluble cortical Aβ40 levels (**Figure 11B**). Soluble cortical Aβ40 levels were higher in NL-G-F/E3 than NL-G-F/E2 (*p* < 0.001, Tukey’s) and NL-G-F/E4 mice (*p* = 0.007, Tukey’s) (**Figure 11B**). In addition, soluble cortical Aβ40 levels were higher in NL-G-F/E4 than NL-G-F/E2 mice (*p* = 0.007, Tukey’s).

There was an effect of genotype on insoluble cortical Aβ42 levels (*F*(2,55) = 3.186, *p* = 0.049). Insoluble cortical Aβ42 levels were higher in NL-G-F/E3 than NL-G-F/E2 mice (*p* = 0.034, Tukey’s) (**Figure 11C**).

For soluble cortical Aβ42 levels, there was also an effect of genotype (*F*(2,56) = 12.369, *p* < 0.001). Insoluble cortical Aβ42 levels were higher in NL-G-F/E3 than NL-G-F/E2 (*p* < 0.001, Tukey’s) and NL-G-F/E4 mice (*p* = 0.002, Tukey’s) (**Figure 11D**).

For the insoluble Aβ42/40 ratio, there was a trend towards an effect of genotype (*F*(2,55) = 3.011, *p* = 0.057), with a trend towards a higher insoluble Aβ42/40 ratio in NL-G-F/E2 than NL-G-F/E3 mice (*p* = 0.063, Tukey’s) (**Figure 11E**).

For the soluble Aβ42/40 ratio, there was no effect of genotype or treatment (**Figure 11F**).

We next analyzed insoluble and soluble cortical p-tau levels. In the NL-G-F/apoE mice, there was a trend towards an effect of treatment in mice for insoluble cortical p-tau levels (*F*(1,29) = 3.612, *p* = 0.067), with a trend towards lower insoluble p-tau levels in KUNV- than PBS-treated NL-G-F/E4 mice (*t* = 2.012, *p* = 0.072) (**Figure 13A**). For the soluble cortical p-tau levels, there was a trend towards an effect of genotype (*F*(2,30) = 2.990, *p* = 0.065), with a trend towards lower soluble p-tau levels in NL-G-F/E4 than NL-G-F/E2 and NL-G-F/E3 mice (**Figure 13B**). For the insoluble/soluble p-tau ratio, there was an effect of treatment (*F*(1,30) = 6.369, *p* = 0.015); the insoluble/soluble p-tau ratio was lower in KUNV- than PBS-treated NL-G-F/E4 mice and there was a trend towards a lower insoluble/soluble p-tau ration in KUNV- than PBS-treated NL-G-F/E3 mice (**Figure 13C**).

In the apoE mice, there were no effects on insoluble cortical p-tau levels (**Figure 13D**). For soluble cortical p-tau levels, there was a trend towards a genotype × treatment interaction (*F*(1,20) = 3.787, *p* = 0.066), with a trend towards higher soluble p-tau levels in KUNV- than PBS-treated E3 mice but lower soluble p-tau levels in KUNV- than PBS-treated E4 mice (**Figure 13E**). There were no effects for the insoluble/soluble tau ratio (**Figure 13F**).

### 3.9 Viral loads in the cortex

We next assessed whether KUNV and genotype affect viral loads in the cortex.

Viral loads in the cortex 7 weeks after treatment are illustrated in **Figure 12**. In analysis 1, there was no difference in the number of mice with viral load between NL-G-F/E3 and either NL-G-F/E4 or NL-G-F/E2 mice, NL-G-F/E2 and NL-G-F/E4 mice, or between E3 and E4 mice (**Figure 12A**). However, more E3 mice had viral load (44%) than NL-G-F/E3 mice (0%) (*p* = 0.0172, two-sided, Fisher’s). There was no difference in NL-G-F/E4 and E4 mice with viral load.

**Figure 12:**
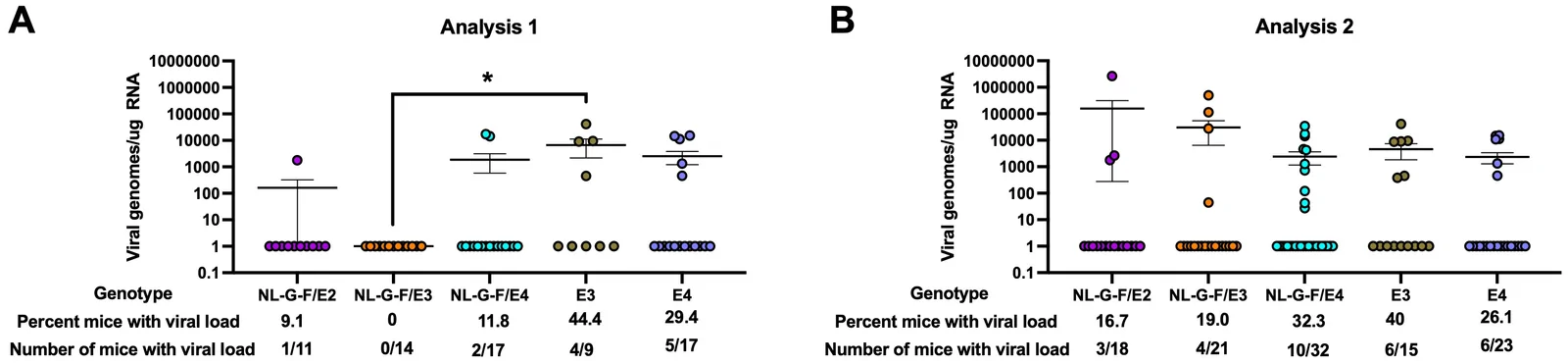
**(A)** NL-G-F/E2, NL-G-F/E3, NL-G-F/E4, E3, and E4 mice with viral load in analysis 1. More E3 mice had viral load than NL-G-F/E3 mice. *p = 0.0172, two-sided, Fisher’s. **(B)** NL-G-F/E2, NL-G-F/E3, NL-G-F/E4, E3, and E4 mice with viral load in analysis 2.

**Figure 13:**
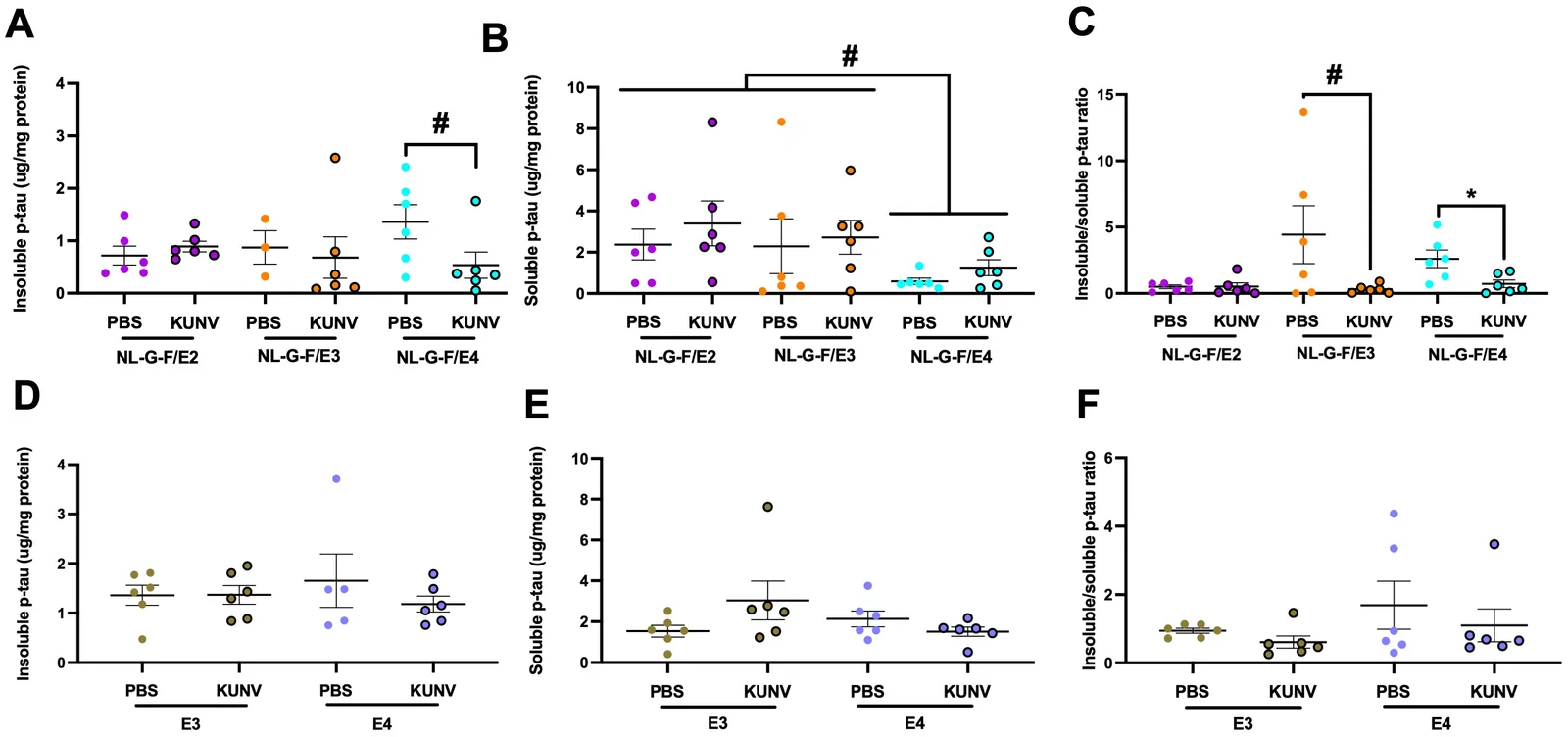
Insoluble and soluble cortical p-tau levels and the insoluble/soluble p-tau ratio in KUNV- and PBS-treated NL-G-F/apoE **(A–C)** and apoE **(D–F)** mice. **(A)** In NL-G-F/apoE mice, there was a trend towards an effect of treatment in mice for insoluble cortical p-tau levels (*F*(1,29) = 3.612, *p* = 0.067), with a trend towards lower insoluble p-tau levels in KUNV- than PBS-treated NL-G-F/E4 mice (*t* = 2.012, ^#^*p* = 0.072). **(B)** For the soluble cortical p-tau levels, there was a trend towards an effect of genotype (*F*(2,30) = 2.990, ^#^*p* = 0.065), with a trend towards lower soluble p-tau levels in NL-G-F/E4 than NL-G-F/E2 and NL-G-F/E3 mice. **(C)** For the insoluble/soluble p-tau ratio, there was an effect of treatment (*F*(1,30) = 6.369, *p* = 0.015); the insoluble/soluble p-tau ratio was lower in KUNV- than PBS-treated NL-G-F/E4 (*t* = 2.627, *p* = 0.0253, *t test*) mice, and there was a trend towards a lower insoluble/soluble p-tau ratio in KUNV- than PBS-treated NL-G-F/E3 mice (*t* = 1.877, *p* = 0.09, *t test*). **(D)** In the apoE mice, there were no effects on insoluble cortical p-tau levels. **(E)** For soluble cortical p-tau levels, there was a trend towards a genotype × treatment interaction (*F*(1,20) = 3.787, *p* = 0.066), with a trend towards higher soluble p-tau levels in KUNV- than PBS-treated E3 mice but lower soluble p-tau levels in KUNV- than PBS-treated E4 mice. **(F)** There were no effects for the insoluble/soluble tau ratio.

In analysis 2, there was no difference in the number of mice with viral load between NL-G-F/E3 and either NL-G-F/E2 or NL-G-F/E3 mice, between NL-G-F/E2 and NL-G-F/E4 mice, or between E3 and E4 mice (**Figure 12B**). There was no difference in NL-G-F/E3 and E3 or between NL-G-F/E4 and E4 mice with viral load.

### 3.10 Immune measures in cortex

Subsequently, we assessed whether KUNV and genotype affect immune measures in the cortex.

#### 3.10.1 Analysis 1

In NL-G-F/apoE mice, there were no significant effects of cortical IFN-γ transcript levels (**Figure 14A**).

**Figure 14:**
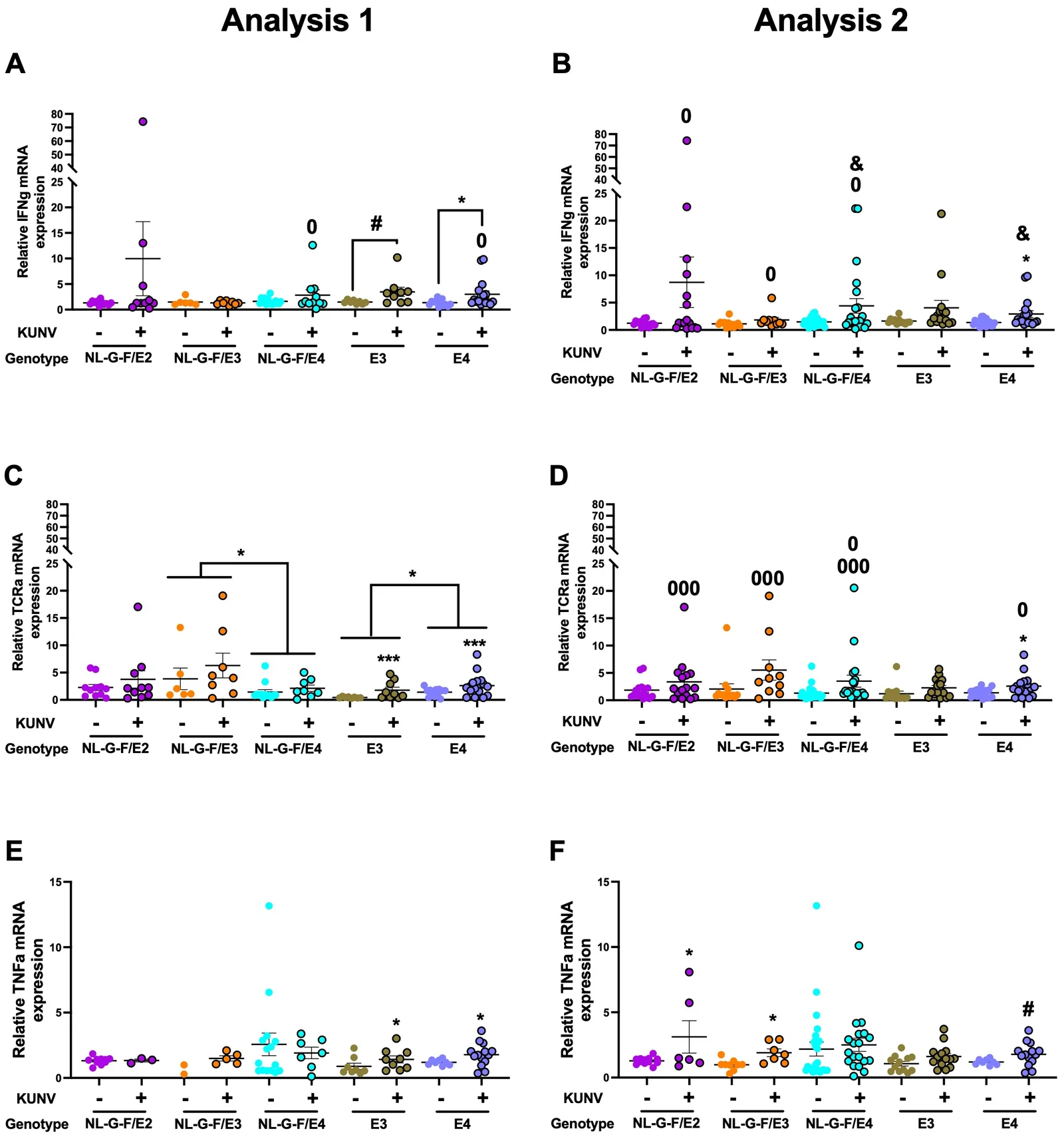
IFN-γ, TCR-α, and TNF-α transcript levels in the cortex of NL-G-F/E2, NL-G-F/E3, NL-G-F/E4, E3 and E4 mice in analysis 1 **(A, C, E)** and analysis 2 **(B, D, F)**. **(A)** IFN-γ transcript levels in analysis 1. In apoE mice, there was an effect of treatment (*F*(1,43) = 8.132, *p* = 0.006) on the cortical levels of IFN-γ transcripts. In E3 mice, there was a trend towards an effect of treatment (*F*(1,15) = 4.177, ^#^*p* = 0.059). In E4 mice, there was an effect of treatment (*F*(1,28) = 4.477, *p = 0.043). When IFN-γ cortical transcript levels in NL-G-F/E4 and E4 mice were compared, there was an effect of treatment (*F*(1,51) = 5.431, ^0^*p* = 0.024). **(B)** IFN-γ transcript levels in analysis 2. In NL-G-F/apoE mice, there was an effect of treatment for cortical IFN-γ transcript levels (*F*(1,96) = 5.67, ^0^*p* = 0.019) with higher IFN-γ transcript levels in KUNV- than PBS-treated mice. In E4 mice, there was an effect of treatment (*F*(1,33) = 5.311, *p = 0.028). When IFN-γ cortical transcript levels in NL-G-F/E4 and E4 mice were compared, there was an effect of treatment (*F*(1,80) = 103.620, ^&^*p* = 0.006). **(C)** TCR-α transcript levels in analysis 1. In NL-G-F/apoE mice, there was an effect of genotype for TCR-α transcript levels (*F*(2,49) = 3.317, *p* = 0.045) with higher levels in NL-G-F/E3 than NL-G-F/E4 mice (*p = 0.032, Tukey’s). In apoE mice, there was an effect of genotype (*F*(1,42) = 6.461, *p = 0.015), with higher TCR-α transcript levels in E4 than E3 mice and an effect of treatment (*F*(1,42) = 9.902, ***p = 0.003), with higher TCR-α transcript levels in KUNV- than PBS-treated mice. **(D)** TCR-α transcript levels in analysis 2. In NL-G-F/apoE mice, there was an effect of treatment for TCR-α transcript levels in the cortex (*F*(1,92) = 9.361, ^000^*p* = 0.003). In E4 mice, there was an effect of treatment (*F*(1,32) = 6.576, *p = 0.015), with higher TCR-α transcript levels in KUNV- than PBS-treated mice. **(E)** TNF-α transcript levels in analysis 1. In apoE mice, there was an effect of treatment (*F*(1,34) = 6.284, *p = 0.017), with higher TNF-α transcript levels in KUNV- than PBS-treated mice. **(F)** TNF-α transcript levels in analysis 2. In NL-G-F/E2 and NL-G-F/E3 mice, there was an effect of treatment (*p < 0.05). In E4 mice, there was a trend towards an effect of treatment, with a trend towards higher TNF-α transcript levels in KUNV- than PBS-treated mice (*F*(1,19) = 3.645, ^#^*p* = 0.071).

In apoE mice, there was an effect of treatment (*F*(1,43) = 8.132, *p* = 0.006) on the cortical levels of IFN-γ transcripts (**Figure 14A**). In E3 mice, there was a trend towards an effect of treatment (*F*(1,15) = 4.177, *p* = 0.059). In E4 mice, there was an effect of treatment (*F*(1,28) = 4.477, *p* = 0.043), with higher IFN-γ transcript levels in KUNV- than PBS-treated mice.

In NL-G-F/apoE mice, there was an effect of genotype for TCR-α transcript levels (*F*(2,49) = 3.317, *p* = 0.045) (**Figure 14C**). TCR-α transcript levels were higher in NL-G-F/E3 than NL-G-F/E4 mice (*p* = 0.032, Tukey’s).

In apoE mice, there was an effect of genotype (*F*(1,42) = 6.461, *p* = 0.015), with higher TCR-α transcript levels in E4 than E3 mice and an effect of treatment (*F*(1,42) = 9.902, *p* = 0.003), with higher TCR-α transcript levels in KUNV- than PBS-treated mice (**Figure 14C**).

In NL-G-F/apoE mice, there were no significant effects for levels of cortical TNF-α transcripts (**Figure 14E**).

In apoE mice, there was an effect of treatment (*F*(1,34) = 6.284, *p* = 0.017), with higher TNF-α transcript levels in KUNV- than PBS-treated mice.

When IFN-γ cortical transcript levels in NL-G-F/E4 and E4 mice were compared, there was an effect of treatment (*F*(1,51) = 5.431, *p* = 0.024), with higher IFN-γ levels in KUNV- than PBS-treated mice (**Figure 14A**).

#### 3.10.2 Analysis 2

In NL-G-F/apoE mice, there was an effect of treatment for cortical IFN-γ transcript levels (*F*(1,96) = 5.67, *p* = 0.019) with higher IFN-γ transcript levels in KUNV- than PBS-treated mice (**Figure 14B**).

In apoE mice or E3 mice, there was no overall effect of treatment for cortical IFN-γ transcript levels. In E4 mice, there was an effect of treatment (*F*(1,33) = 5.311, *p* = 0.028) (**Figure 14B**).

In NL-G-F/apoE mice, there was an effect of treatment for TCR-α transcript levels in the cortex (*F*(1,92) = 9.361, *p* = 0.003) (**Figure 14D**).

In apoE mice, there was no overall effect of treatment for TCR-α transcript levels in the cortex (**Figure 14D**), with higher TCR-α transcript levels in KUNV- than PBS-treated mice. In E3 mice, there were no significant effects (**Figure 14D**). In E4 mice, there was an effect of treatment (*F*(1,32) = 6.576, *p* = 0.015), with higher TCR-α transcript levels in KUNV- than PBS-treated mice (**Figure 14D**).

In NL-G-F/apoE mice, there was a trend towards an effect of treatment for cortical TNF-α transcript levels in the cortex (*F*(1,73) = 3.566, *p* = 0.063) (**Figure 14F**). In NL-G-F/E2 mice, there was an effect of treatment (*F*(1,16) = 4.582, *p* = 0.048). In NL-G-F/E3 mice, there was an effect of treatment (*F*(1,13) = 5.294, *p* = 0.039). In NL-G-F/E4 mice, there were no significant effects.

In apoE mice, there was no overall effect of treatment for TNF-α transcript levels in the cortex. In E3 mice, there was no significant effect. In E4 mice, there was a trend towards an effect of treatment, with a trend towards higher TNF-a transcript levels in KUNV- than PBS-treated mice (*F*(1,19) = 3.645, *p* = 0.071).

When IFN-γ cortical transcript levels in NL-G-F/E4 and E4 mice were compared, there was an effect of treatment (*F*(1,80) = 103.620, *p* = 0.006). For TCR-α cortical transcript levels, there was an effect of treatment (*F*(1,74) = 6.886, *p* = 0.011). For TNF-α cortical transcript levels, there were no significant effects.

### 3.11 Immune measures in cortex by viral load

#### 3.11.1 Analysis 1

As only one NL-G-F/E2, no NL-G-F/E3, and two NL-G-F/E4 showed viral loads in analysis, we limited this analysis to E3 and E4 mice. IFN-γ transcript levels were not different in E3 mice with and without viral loads (**Figure 15A**) but higher in E4 mice with viral loads than those without detectable viral load (*t* = 3.024, *p* = 0.0376, two-tailed *t test*) (**Figure 15B**).

**Figure 15:**
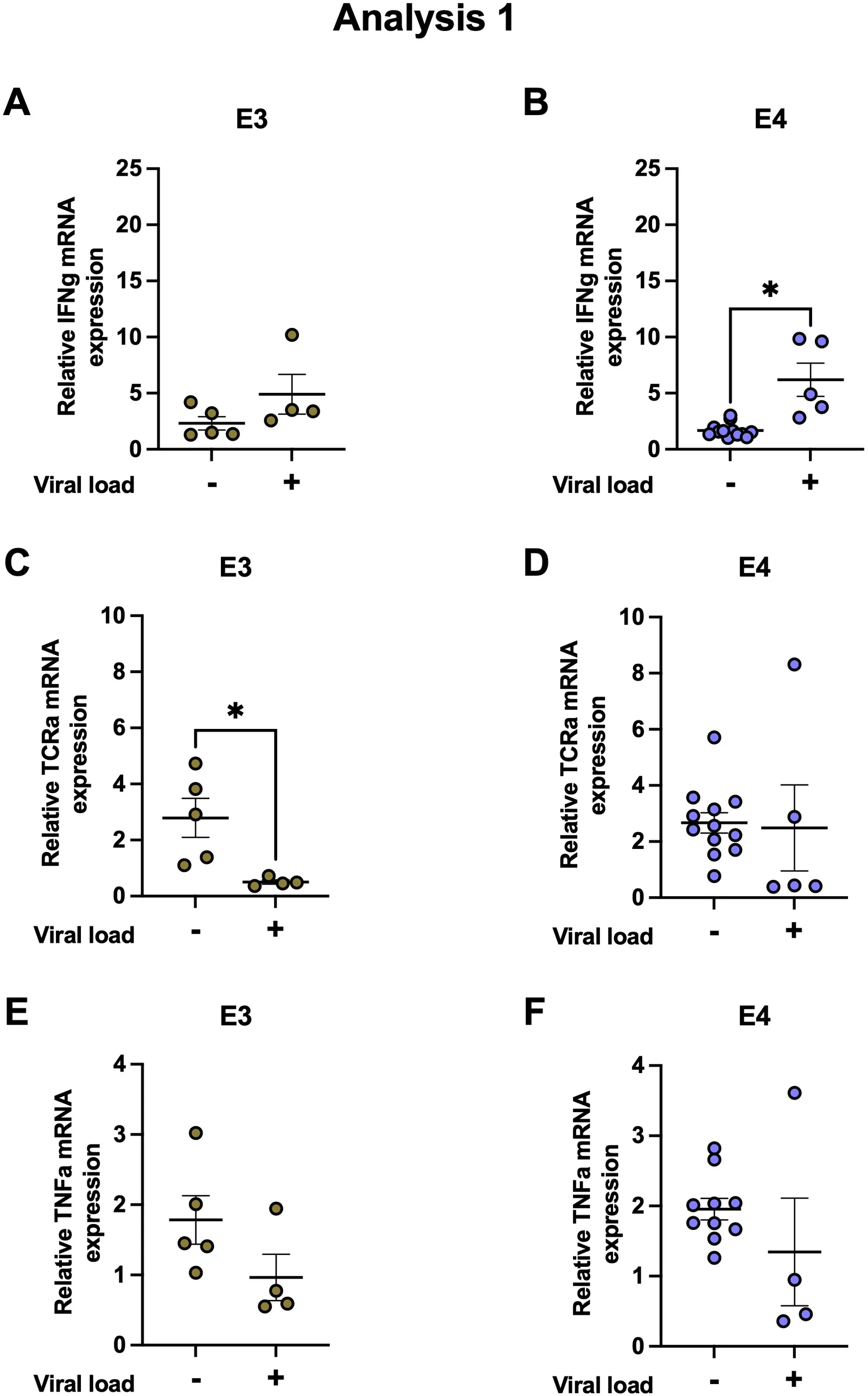
IFN-γ **(A, B)**, TCR-α **(C, D)**, and TNF-α **(E, F)** transcript levels in the cortex of E3 **(A, C, E)** and E4 **(B, D, F)** mice in analysis 1. **(B)** TCR-α transcript levels were higher in E4 mice with viral loads than those without detectable viral load (*t* = 3.024, *p = 0.0376, two-tailed *t test*). **(C)** TCR-α transcript levels were lower in E3 mice with viral load than those without viral loads (*t* = 3.269, *p = 0.0298, two-tailed *t test*).

TCR-α transcript levels were lower in E3 mice with viral load than those without viral loads (*t* = 3.269, *p* = 0.0298, two-tailed *t test*) (**Figure 15C**) but not different in E4 mice with and without viral loads (**Figure 15D**).

TNF-α transcript levels were not different in E3 (**Figure 15E**) or E4 (**Figure 15F**) mice with and without viral loads.

#### 3.11.2 Analysis 2

As only two NL-G-F/E3 mice showed viral loads (**Supplementary Figures S9B, G, L**), they were excluded from the statistical analysis. As we had only one data point for TNF-α transcript levels in a mouse with viral loads in NL-G-F/E2 mice (**Supplementary Figure S9K**), we excluded this from statistical analysis as well. IFN-γ transcript levels were higher in NL-G-F/E4 mice with viral loads than those without it (*t* = 2.583, *p* = 0.0288, two-tailed *t test*) (**Supplementary Figure S9C**) and higher in E4 mice with viral loads than those without it (*t* = 3.046, *p* = 0.0369, two-tailed *t test*) (**Supplementary Figure S9E**). No significant differences in IFN-γ transcript levels were seen in mice with and without viral loads in NL-G-F/E2 (**Supplementary Figure S9A**) and E3 (**Supplementary Figure S9D**) mice.

TCR-α transcript levels were lower in E3 mice with viral loads than without viral loads (*t* = 2.861, *p* = 0.0134, two-tailed *t test*) (**Supplementary Figure S9I**). No significant differences in TCR-α transcripts were seen in mice with and without viral loads in NL-G-F/E2 (**Supplementary Figure S9F**), NL-G-F/E4 (**Supplementary Figure S9H**), and E4 (**Supplementary Figure S9J**) mice.

TNF-α transcript levels were not different in mice with and without viral loads in any genotype (**Supplementary Figures S9M–O**).

### 3.12 PCAs analyze

#### 3.12.1 PCA analysis 1: NL-G-F/ApoE mice only

This analysis revealed eight factors with eigenvalues >1.0, which explained a total of 69.16% of the variance among the measures in the model (**Supplementary Table S26**). The eight factors explained 23.56%, 9.61%, 7.64%, 7.04%, 6.00%, 5.47%, 5.07%, and 4.78% of the variance, respectively.

Measures that entered into the model were as follows: genotype (ApoE), distance moved in the open field on day 1 (activity measure), time spent in the center of the open field on day 1 (anxiety measure), difference in activity levels in the open field between days 1 and 2 divided by activity levels on day 1 (spatial habituation: cognitive measure), arm entries in the Y maze (activity measure), spontaneous alternation in the Y maze (cognitive measures), time spent (s) in the novel arm on the spatial Y maze, cortical levels of soluble and insoluble p-tau, Aβ40 and Aβ42 (only for the PCA with only NL-G-F/apoE mice), and cortical transcript levels of IFN-γ, TCR-α, and TNF-α levels.

ApoE genotype and body temperatures during the dark and light periods loaded on factor 1. The directions of the component loadings in factor 1 were such that increasing values of the factor (E4 > E3 > E2) increased the mean body temperatures.

Distance moved in the open field on day 1, arm entries in the Y maze, and soluble cortical p-tau levels loaded on factor 2. The directions of the component loadings in factor 2 were such that increases in activity levels in the open field and in the Y maze increased soluble cortical p-tau levels.

Cortical IFN-γ, TCR-α, and TNF-α loaded on factor 3. The directions of the component loadings in factor 3 were such that increases in immune measures of one of the immune measures increases the levels of the other two.

Soluble and insoluble cortical Aβ42 levels loaded on factor 4. The directions of the component loadings in factor 4 were such that increases in soluble Aβ42 increased insoluble Aβ42 levels.

Time spent in the center of the open field and spontaneous alternation in the Y maze loaded on factor 5. The directions of the component loadings in factor 5 were such that increases in time spent in the center of the open increased spontaneous alternation in the Y maze.

Spatial habituation in the open field, time spent in the novel arm of the spatial Y maze, and cortical insoluble Aβ40 levels loaded on factor 6. The directions of the component loadings in factor 6 were such that decreases in spatial habituation increased cortical insoluble Aβ40 levels.

Time spent in the novel arm of the spatial Y maze and soluble cortical Aβ40 levels loaded on factor 7. The directions of the component loadings in factor 7 were such that increases in time spent in the novel arm in the spatial Y maze increased cortical soluble Aβ40 levels.

ApoE genotype and insoluble cortical p-tau levels loaded on factor 8. The directions of the component loadings in factor 8 were such that decreases in *ApoE* genotype (E2 < E3 < E4) increased insoluble cortical p-tau levels.

The data of PCA analysis 1 suggest a detrimental effect of cortical insoluble Aβ40 levels and beneficial effects of soluble Aβ40 and soluble p-tau levels on behavioral performance. In contrast to NL-G-F/apoE mice, soluble p-tau levels did not load on a factor that behavioral measures loaded on in apoE mice.

#### 3.12.2 PCA analysis 2: ApoE mice only (without NL-G-F)

This analysis revealed seven factors with eigenvalues >1.0, which explained a total of 77.94% of the variance among the measures in the model (**Supplementary Table S27**). The seven factors explained 28.96%, 12.49%, 9.85%, 7.65%, 6.97%, 6.39%, and 5.62% of the variance, respectively.

Measures entered into the model were: genotype (ApoE), distance moved in the open field on day 1 (activity measure), time spent in the center of the open field on day 1 (anxiety measure), difference in activity levels in the open field between days 1 and 2 divided by activity levels on day 1 (spatial habituation: cognitive measure), arm entries in the Y maze (activity measure), spontaneous alternation in the Y maze (cognitive measures), time spent (s) in the novel arm on the spatial Y maze, cortical levels of soluble and insoluble p-tau, Aβ40 and Aβ42 (only for the PCA with only NL-G-F/apoE mice), and cortical transcript levels of IFN-γ, TCR-α, and TNF-α levels.

Mean body temperatures in the dark and light periods loaded on factor 1. The directions of the component loadings in factor 1 were such that increases in body temperatures in the dark periods increased body temperatures in the light periods and this pattern was consistent across weeks as well.

Treatment (viral exposure or PBS) and spontaneous alternation in the Y maze, IFN-γ and TNF-α transcript levels in the cortex loaded on factor 2. The directions of the component loadings in factor 2 were such that viral exposure decreased spontaneous alternation in the Y maze and increased IFN-γ and TNF-α transcript levels in the cortex.

ApoE genotype, distance moved in the open field on day 1, and arm entries in the Y maze loaded on factor 3. The directions of the component loadings in factor 3 were such that decreases in ApoE genotype (E2 < E3 < E4) increased activity levels in the open field on day 1 and increased arm entries in the Y maze.

Spontaneous alternation in the Y maze and time spent in the novel arm of the spatial Y maze loaded on factor 4. The directions of the component loadings in factor 4 were such that decreases in spontaneous alternation in the regular Y maze (a measure of spatial working memory) increased time spent in the novel arm in the spatial Y maze (a measure of long-term (24-h) spatial memory retention).

Spatial habituation, soluble and insoluble cortical p-tau levels, and cortical TCR-α transcript levels loaded on factor 5. The directions of the component loadings in factor 5 were such that decreases in spatial habituation increased TCR-α transcript levels in the cortex.

Soluble and insoluble cortical p-tau levels loaded on factor 6. The directions of the component loadings in factor 5 were such that increases in soluble p-tau levels increased insoluble p-tau levels.

In PCA analysis 2, soluble and insoluble cortical p-tau levels loaded on the same factor, and the directions of the component loadings were such that increases in soluble p-tau levels increased insoluble p-tau levels.

p-tau immunotherapy reduced both insoluble and soluble p-tau levels in aged 3XTg-AD mice (47). We recognize that we analyzed p-tau levels in the whole cortex, and it is conceivable that in subregions in cortex, there might distinct relationships between p-tau levels and behavioral measures. In addition, it is conceivable that there might be important species differences in the relationship between soluble p-tau levels and behavioral measures in humans and animal models.

Time spent in the center of the open field on day 1, a measure of anxiety, and spatial habituation loaded on factor 7. The directions of the component loadings in factor 7 were such that increases in time spent in the center of the open field, reflecting reduced anxiety levels, increased spatial habituation.

#### 3.12.3 PCA analysis 3: all mice

This analysis revealed six factors with eigenvalues >1.0, which explained a total of 65.35% of the variance among the measures in the model (**Supplementary Table S28**). The six factors explained 28.15%, 10.76%, 8.53%, 6.27%, 5.99%, 7%, and 5.67% of the variance.

Measures entered into the model were as follows: genotype (ApoE), NL-G-F, treatment, distance moved in the open field on day 1 (activity measure), time spent in the center of the open field on day 1 (anxiety measure), difference in activity levels in the open field between days 1 and 2 divided by activity levels on day 1 (spatial habituation: cognitive measure), arm entries in the Y maze (activity measure), spontaneous alternation in the Y maze (cognitive measures), time spent (s) in the novel arm on the spatial Y maze, cortical levels of soluble and insoluble p-tau, Aβ40, and Aβ42 (only for the PCA with only NL-G-F/apoE mice), and cortical transcript levels of IFN-γ, TCR-α, and TNF-α levels.

Mean body temperatures in the dark and light periods loaded on factor 1. The directions of the component loadings in factor 1 were such that increases in body temperatures in the dark periods increased body temperatures in the light periods, and this pattern was consistent across weeks as well.

ApoE genotype, NL-G-F, treatment, distance moved in the open field on day 1, arm entries in the Y maze, and soluble cortical p-tau levels loaded on factor 2. The directions of the component loadings in factor 2 were such that decreases in ApoE genotype (E2 < E3 < E4) decreased the NL-G-F genotype (NL-G-F/E2 < NL-G-F/E3 < NL-G-F/E4) and increased activity levels in the open field on day 1, arm entries in the Y maze, and soluble cortical p-tau levels.

Treatment and cortical transcripts of IFN-γ, TCR-α, and TNF-α loaded on factor 3. The directions of the component loadings in factor 3 were such that increases in treatment (viral exposure) increased cortical transcripts of IFN-γ, TCR-α, and TNF-α.

Time spent in the center of the open field on day 1, a measure of anxiety, and spatial habituation loaded on factor 4. The directions of the component loadings in factor 4 were such that increases in time spent in the center of the open field, reflecting reduced anxiety levels, increased spatial habituation.

Treatment and cortical insoluble p-tau levels loaded on factor 5. The directions of the component loadings in factor 5 were such that decreases in treatment (PBS < KUNV) increased insoluble cortical p-tau levels in the cortex.

Spontaneous alternation in the Y maze and time spent in the novel arm of the spatial Y maze loaded on factor 6. The directions of the component loadings in factor 6 were such that decreases in spontaneous alternation in the regular Y maze (a measure of spatial working memory) increased time spent in the novel arm in the spatial Y maze. (a measure of long-term (24-h) spatial memory retention).

In the PCA of all the mice combined, ApoE genotype, NL-G-F, treatment, distance moved in the open field on day 1, arm entries in the Y maze, and soluble cortical p-tau levels loaded on the same factor and the directions of the component loadings were such that increases in soluble cortical p-tau levels increased activity levels in the open field on day 1 and arm entries in the Y maze. Treatment and cortical insoluble p-tau levels loaded on the same factor and the directions of the component loadings were such that decreases in treatment (PBS < KUNV) increased insoluble cortical p-tau levels in the cortex. These results suggest that soluble p-tau levels might be more important for modulating behavioral measures than insoluble p-tau levels. Consistent with this pattern, insoluble cortical p-tau levels did not load on the same factor as behavioral mice in the PCA of NL-G-F/apoE mice or apoE mice. This is an important observation based on the tight link between tau pathology and cognitive injury in AD (48) and the therapeutic strategies to reduce or even eliminate hyperphosphorylated tau (14–16).

In the PCA of NL-G-F/apoE mice, IFN-γ, TCR-α, and TNF-α transcript levels loaded on the same factor, but no other measure loaded on that factor. In the PCA of all mice combined, treatment loaded on the same factor as the three immune measures. However, in the PCA of apoE mice, treatment (viral exposure or PBS), spontaneous alternation in the Y maze, IFN-γ and TNF-α transcript levels in the cortex loaded on the same factor, and the directions of the component loadings were such that viral exposure decreased spontaneous alternation in the Y maze and increased IFN-γ and TNF-α transcript levels in the cortex. In addition, spatial habituation, soluble and insoluble cortical p-tau levels, and cortical TCR-α transcript levels loaded on the same factor and the directions of the component loadings such that decreases in spatial habituation increased TCR-α transcript levels in the cortex. These results suggest a protective effect of NL-G-F following KUNV exposure.

In the PCA in apoE mice and in the PCA in all mice combined, the mean body temperatures in the light and dark periods in the different weeks all loaded on the same factor and other behavioral, immune, or pathological measures did not lead on this factor. However, in the PCA in NL-G-F/apoE mice, ApoE genotype and body temperatures during the dark and light periods loaded on the same factor, and the directions of the component loadings were such that increasing values of the factor (E4 > E3 > E2) increased the mean body temperatures. These results suggest that the presence of NL-G-F might be required to detect apoE genotype differences in body temperatures. In analysis 2, body temperatures in NL-G-F/E2 mice during the dark and light periods in weeks 1 and 2 were lower than those in NL-G-F/E3 or NL-G-F/E4 mice. In week 4 during the dark periods, body temperatures were lower in NL-G-F/E2 than NL-G-F/E4 mice with a trend towards lower body temperatures in NL-G-F/E2 than NL-G-F/E3 mice. This genotype difference was more subtle in younger mice. In analysis 1, in week 2 and during the dark periods, there was an effect of genotype but only a trend towards lower body temperatures in NL-G-F/E2 than NL-G-F/E3 mice. The lower body temperatures seen in NL-G-F/E2 mice are consistent with a neuroprotective role of E2, as in humans without and with mild cognitive impairment (MCI), lower body temperatures were associated with better cognitive performance (49). Remarkably, in contrast to the human data, we did not see mean body temperatures in the dark or light periods loading on the same factor as behavioral, pathological, or immune measures in the same mice in any of three PCAs.

### 3.13 Study 2

To assess early effects of KUNV exposure, we assessed insoluble and soluble murine p-tau levels in the cortex 6 days following KUNV or PBS treatment. Genotype and treatment effects are described below. Effects of sex or interaction with sex are described in the supplemental document.

For insoluble p-tau levels, there was an effect of NL-G-F (*F*(1,60) = 7.561, *p* = 0.008), with lower levels in NL-G-F/apoE than apoE mice, treatment (*F*(1,60) = 34.623, *p* < 0.001), with lower levels in KUNV- than PBS-treated NL-G-F/E2, NL-G-F/E3, and NL-G-F/E3/E4 mice, a NL-G-F × treatment interaction (*F*(1,60) = 14.503, *p* < 0.001), with robust treatment effects in apoE but not in NL-G-F/apoE mice, and a trend towards an effect of genotype (*F*(3,60) = 2.258, *p* = 0.091) (**Figures 16A, B**). When insoluble p-tau levels in only apoE mice with NL-G-F were analyzed, there was no effect of treatment. When insoluble p-tau levels in only apoE mice without NL-G-F were analyzed, there was an effect of treatment (*F*(1,30) = 48.324, *p* < 0.001) with lower levels in NL-G-F- than PBS-treated mice (**Figure 16B**).

**Figure 16:**
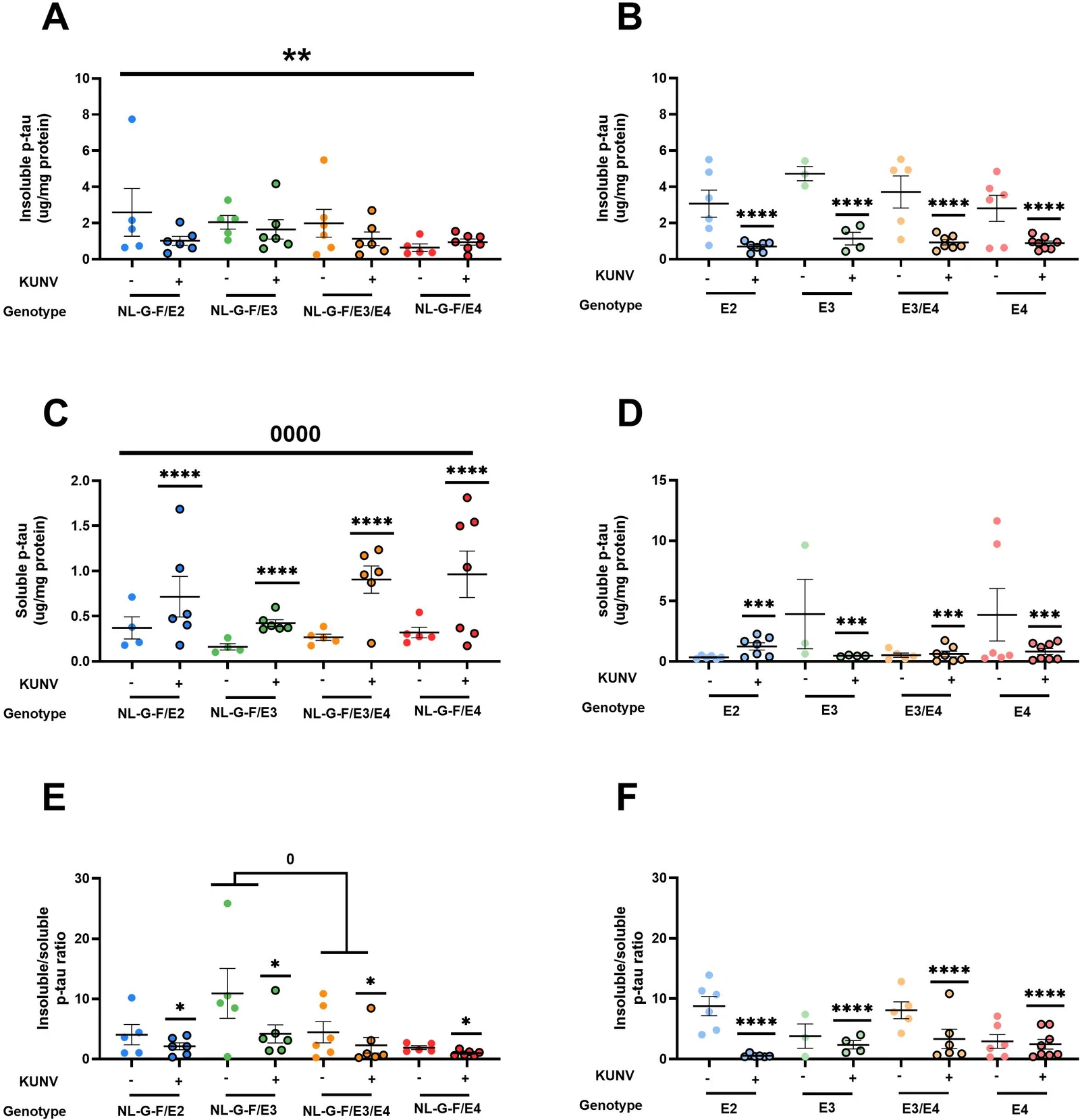
Cortical insoluble p-tau **(A, B)**, soluble p-tau **(C, D)**, and the insoluble/soluble p-tau ratio **(E, F)** in NL-G-F/apoE **(A, C, E)** and apoE **(B, D, F)** mice. **(A, B)** Insoluble p-tau levels, there was an effect of NL-G-F (*F*(1,60) = 7.561, **p = 0.008), with lower levels in NL-G-F/apoE than apoE mice. **(A)** When insoluble p-tau levels in only apoE mice with NL-G-F were analyzed, there was no effect of treatment. **(B)** When insoluble p-tau levels in only apoE mice without NL-G-F were analyzed, there was an effect of treatment (*F*(1,30) = 48.324, ****p < 0.001) with lower levels in NL-G-F- than PBS-treated mice. **(C, D)** For soluble p-tau levels, there was an effect of NL-G-F (*F*(1,57) = 17.405, ^0000^*p* < 0.001), with higher levels in apoE- than NL-G-F/apoE mice, apoE genotype (*F*(3,57) = 4.093, *p* = 0.011). **(C)** When soluble p-tau levels in only apoE mice with NL-G-F were analyzed, there was an effect of treatment (*F*(1,27) = 14.980, ****p < 0.001), with higher levels in KUNV- than PBS-treated mice. **(D)** When soluble p-tau levels in only apoE mice without NL-G-F were analyzed, there also was an effect of treatment (*F*(1,30) = 10.710, ***p = 0.003). **(E, F)** When the insoluble/soluble p-tau ratios in only apoE mice with NL-G-F were analyzed, there was an effect of genotype (*F*(3,30) = 3.810, *p* = 0.020), with higher ratios in E3/E3 than E4/E4 mice (^0^*p* = 0.027, Tukey’s) and an effect of treatment (*F*(1,30) = 4.296, *p = 0.047). When the insoluble/soluble p-tau ratios in only apoE mice without NL-G-F were analyzed, there was an effect of treatment (*F*(1,28) = 17.574, ****p < 0.001).

For soluble p-tau levels, there were three outliers in the NL-G-F/apoE mice (ROUT test) that were removed from the analysis. There was an effect of NL-G-F (*F*(1,57) = 17.405, *p* < 0.001), with higher levels in apoE- than NL-G-F/apoE mice, apoE genotype (*F*(3,57) = 4.093, *p* = 0.011), treatment (*F*(1,57) = 5.176, *p* = 0.027), with higher levels in KUNV- than PBS-treated mice, a NL-G-F × apoE genotype interaction (*F*(1,57) = 4.571, *p* = 0.006), NL-G-F × treatment interaction (*F*(1,57) = 15.730, *p* < 0.001), apoE genotype × treatment interaction (*F*(3,57) = 5.322, *p* = 0.003), a NL-G-F × apoE genotype × treatment interaction (*F*(3,57) = 5.585 *p* = 0.002) (**Figures 16C, D**).

When soluble p-tau levels in only apoE mice with NL-G-F were analyzed, there was an effect of treatment (*F*(1,27) = 14.980, *p* < 0.001), with higher levels in KUNV- than PBS-treated mice (**Figure 16C**).

When soluble p-tau levels in only apoE mice without NL-G-F were analyzed, there was an effect of apoE genotype (*F*(3,30) = 4.758, *p* = 0.008), with a trend towards a difference between E3/E4 and E4/E4 (*p* = 0.096, Tukey’s), treatment (*F*(1,30) = 10.710, *p* = 0.003), with higher levels in KUNV- than PBS-treated E2 and E4 mice, and an apoE genotype × treatment interaction (*F*(3,30) = 6.003, *p* = 0.002).

For the insoluble/soluble p-tau ratio, there were two outliers in the apoE mice (ROUT). There was an effect of treatment (*F*(1,58) = 15.290, *p* < 0.001), with lower levels in KUNV- than PBS-treated mice, a NL-G-F × apoE genotype interaction (*F*(3,58) = 3.757, *p* = 0.016), a trend towards an effect of genotype (*F*(3,58) = 2.629, *p* = 0.059), and a trend towards a NL-G-F × apoE genotype × treatment interaction (*F*(3,58) = 2.351, *p* = 0.082).

When the insoluble/soluble p-tau ratios in only apoE mice with NL-G-F were analyzed, there was an effect of genotype (*F*(3,30) = 3.810, *p* = 0.020), with a difference between E3/E3 and E4/E4 (*p* = 0.027, Tukey’s), and an effect of treatment (*F*(1,30) = 4.296, *p* = 0.047), with a lower ratio in KUNV- than PBS-treated mice.

When the insoluble/soluble p-tau ratios in only apoE mice without NL-G-F were analyzed, there was an effect of genotype (*F*(3,28) = 3.757, *p* = 0.022), with a trend towards a difference between E3/E4 and E4/E4 (*p* = 0.060, Tukey’s), treatment (*F*(1,28) = 17.574, *p* < 0.001), with a lower ratio in KUNV- than PBS-treated mice, and a genotype × treatment interaction (*F*(3,28) = 5.032, *p* = 0.006).

## 4 Discussion

The main effects of KUNV exposure are summarized in **Table 2**. These data show that apoE isoform-dependent responses to KUNV exposure on various measures are already seen at 6 months of age and are mostly more profound in the absence than in the presence of NL-G-F. While in analysis 1 activity levels were lower in KUNV- than PBS-treated E3 and E4 mice, they were not altered in NL-G-F/E3 and NL-G-F/E4 mice. In addition, in analysis 1, spontaneous alternations were lower in KUNV- than PBS-treated E3 and E4 mice but not altered in NL-G-F/E3 and NL-G-F/E4 mice. Furthermore, in the spatial Y maze in analysis 1, the time spent in the novel arm was higher in KUNV- than PBS-treated E3 and E4 mice but not altered in NL-G-F/E3 and NL-G-F/E4 mice. In analysis 1, viral loads at 7 weeks were higher in KUNV-treated E3 than NL-G-F/E3 mice. Also, in analysis 1 cortical IFN-γ TCR−α transcript levels were higher in KUNV- than PBS-treated E3 and E4 mice, but this was not seen in NL-G-F/E3 or NL-G-F/E4 mice. In analysis 1, in week 4, there was a day-by-treatment interaction for body temperatures in E4 mice and this was not seen in NL-G-F/E4 mice, with higher body temperatures in KUNV- than PBS-treated mice. Although it did not reach significance, in analysis 1, a trend towards higher body temperatures in KUNV- than PBS-treated E4 mice without this pattern showing in NL-G-F/E4 mice was also seen in weeks 1 and 2. In study 2, cortical insoluble p-tau levels were lower in KUNV- than PBS-treated apoE, but not NL-G-F/apoE mice. Soluble p-tau levels in the cortex were higher in KUNV- than PBS-treated mice, and this effect was more pronounced in NL-G-F/apoE than apoE mice. The insoluble/soluble p-tau ratio was lower in KUNV- than PBS-treated mice, and this effect was more pronounced in apoE than NL-G-F/apoE mice. These results indicate that the presence of NL-G-F is not required to detect apoE isoform-dependent differences in response to KUNV. The apoE isoform-dependent differences in response to KUNV exposure illustrate the importance of considering *APOE* genotyping following viral exposure in humans. ApoE isoform critically modulated whether there was an effect of viral exposure as well as the direction of the effect for a given outcome measure. Consistent with this result, apoE isoform differences in viral replication in brain were seen in a mouse model of HSV-1 (24, 25), entry of HIV-1 and HIV-1 disease progression (26).

**Table 2:** Summary of the data.

Measure	NL-G-F/E2	NL-G-F/E3	NL-G-F/E4	Genotype comparison	E3	E4	Genotype comparison
Activity levels in the open field		*Analysis 2:* Males: KUNV > PBS A day × treatment interaction	*Analysis 2:* Males: KUNV < PBS Females: young < old	*Analysis 2:* NL-G-F/E3 > NL-G-F/E4; NL-G-F/E3 > NL-G-F/E2 In NL-G-F/E3 and NL-G-F/E4: day × genotype × treatment interaction	*Analysis 1:* KUNV < PBS *Analysis 2:* females: day × age interaction	*Analysis 1:* KUNV < PBS	
Spatial habituation learning		*Analysis 1:* impairment in PBS- but not in KUNV-treated mice					
Time spent in the center of the open field	*Analysis 2:* KUNV < PBS		*Analysis 2:* Day × age interaction	*Analysis 1:* NL-G-F/E2 < NL-G-F/E3; NL-G-F/E2 < NL-G-F/E4 *Analysis 2:* NL-G-F/E2 < NL-G-F/E3; NL-G-F/E2 < NL-G-F/E4 Day × age interaction			*Analysis 1:* E3 > E4
Activity levels in the open field containing objects	Day × treatment interaction		Day × age interaction	*Analysis 2:* NL-G-F/E3 > NL-G-F/E2; NL-G-F/E3 > NL-G-F/E4 Day × age interaction Day × genotype interaction Effect of age: young < old			*Analysis 2:* day × genotype × treatment interaction
Time in the center of the open field containing object	*Analysis 2:* females: KUNV < PBS *Analysis 2:* effect of age: young < old		*Analysis 1:* day × treatment interaction	*Analysis 1:* NL-G-F/E2 < NL-G-F/E3; NL-G-F/E2 < NL-G-F/E4 *Analysis 2:* NL-G-F/E2 < NL-G-F/E4			*Analysis 1:* day × genotype × treatment interaction
Time spent exploring objects on the object recognition day	*Analysis 2:* effect of age: young < old	*Analysis 2:* effect of age: young < old	*Analysis 2:* effect of age: young < old	*Analysis 1:* NL-G-F/E2 < NL-G-F/E3; NL-G-F/E2 < NL-G-F/E4 *Analysis 2:* NL-G-F/E2 < NL-G-F/E3; NL-G-F/E2 < NL-G-F/E4 Effect of age: young < old Day × age interaction Day × genotype interaction			*Analysis 2:* E3 < E4
Discrimination index			*Analysis 2:* effect of age: old < young				
Spontaneous alternation in the regular Y maze	*Analysis 2:* KUNV < PBS Effect of age: old > young		*Analysis 2:* KUNV < PBS	*Analysis 1:* genotype × treatment interaction *Analysis 2:* NL-G-F/E2 > NL-G-F/E4 *Analysis 3:* NL-G-F/E2 > NL-G-F/E3; NL-G-F/E2 > NL-G-F/E4		*Analysis 2:* KUNV < PBS	*Analysis 1:* Effect of treatment; KUNV < PBS *Analysis 2:* effect of age: old > young
Spatial Y maze; time spent in novel arm				*Analysis 2:* effect off age: young > old	*Analysis 1:* KUNV > PBS	*Analysis 1:* KUNV > PBS	*Analysis 1:* effect of treatment: KUNV > PBS
Spatial Y maze; percent entries in novel arm			*Analysis 2:* effect off age: young > old	*Analysis 2:* effect off age: young > old			
Circadian body temperatures	*Analysis 1:* *Weeks 1 and 2 light:* KUNV > PBS *Analysis 2* *Week 2 dark:* KUNV > PBS :	*Analysis 1:* *Week 1 dark:* KUNV < PBS *Analysis 2:* *Week 1 dark:* KUNV < PBS *Week 1 light:* KUNV > PBS		*Analysis 1:* *Week 1 dark:* treatment x genotype interaction Analysis 2: *Weeks 1 and 2 dark:* effect of genotype: NL-G-F/E2 < NL-G-F/E3; NL-G-F/E2 < NL-G-F/E4 *Weeks 1 and 2 dark:* effect of genotype: NL-G-F/E2 < NL-G-F/E4		*Analysis 2:* *Week 1 dark:* KUNV > PBS *Weeks 2 and 3 dark:* KUNV < PBS *Week 2 light:* KUNV > PBS *Week 4 dark:* KUNV > PBS	
Insoluble cortical Aβ40 levels				Effect of genotype: NL-G-F/E3 > NL-G-F/E2; NL-G-F/E3 > NL-G-F/E4			
Soluble cortical Aβ40 levels				Effect of genotype: NL-G-F/E3 > NL-G-F/E2; NL-G-F/E3 > NL-G-F/E4; NL-G-F/E4 > NL-G-F/E2			
Insoluble cortical Aβ42 levels				Effect of genotype: NL-G-F/E3 > NL-G-F/E2			
Soluble cortical Aβ42 levels				Effect of genotype: NL-G-F/E3 > NL-G-F/E2; NL-G-F/E3 > NL-G-F/E4			
Insoluble/soluble cortical p-tau ratio			PBS > KUNV				
Viral load in cortex at 7 weeks post-inoculation		*Analysis 1:* E3 > NL-G-F/E3			*Analysis 1:* E3 > NL-G-F/E3		
Cortical IFN-γ transcript levels	*Analysis 2:* KUNV > PBS	*Analysis 2:* KUNV > PBS	*Analysis 2:* KUNV > PBS			*Analysis 1:* KUNV > PBS	
Cortical IFN-γ transcript levels by viral load			*Analysis 2:* KUNV > PBS			*Analyses 1 and 2:* KUNV > PBS	
Cortical TCR-α transcript levels	*Analysis 2:* KUNV > PBS	*Analysis 2:* KUNV > PBS	*Analysis 2:* KUNV > PBS	*Analysis 1:* NL-G-F/E3 > NL-G-F/E4	*Analysis 1:* KUNV > PBS	*Analysis 1:* KUNV > PBS *Analysis 2:* KUNV > PBS	*Analysis 1:* E4 > E3
Cortical TCR-α transcript levels by viral load					*Analyses 1 and 2:* KUNV < PBS		
Cortical TNF-α transcript levels	*Analysis 2:* KUNV > PBS	*Analysis 2:* KUNV > PBS	*Analysis 2:* KUNV > PBS		*Analysis 1:* KUNV > PBS	*Analysis 1:* KUNV > PBS	
Study 2 insoluble cortical p-tau levels				NL-G-F/apoE < apoE			KUNV < PBS
Study 2 soluble cortical p-tau levels	KUNV > PBS	KUNV > PBS	KUNV > PBS		KUNV < PBS	KUNV > PBS	
Study 2 insoluble/soluble cortical p-tau ratio							

The results of the current also indicate that effects of KUNV exposure are more profound in the absence than presence of NL-G-F. To the best of our knowledge, this is the first study in which effects of a virus are being compared in the context of human apoE isoforms and in the presence as compared with the absence of human APP/Aβ. Therefore, future efforts are warranted to determine whether this result is specific for KUNV or also seen following exposure to other neurotropic viruses and limited to the NL-G-F AD model.

HIV-1 infection of human APP immunodeficient mice increased amyloid pathology (50). In addition, human APP enhanced cellular entry of Severe Acute Respiratory Syndrome Coronavirus 2 (SARS-CoV-2) and enhanced amyloid pathology in the APP/PS1 AD mouse model (51). Typically, enhanced AD pathology is thought of as being detrimental and reduction of AD pathology, especially reduction of tau pathology (52), as being beneficial. However, based on the results of the current study showing more profound effects of KUNV in the absence than presence of NL-G-F and results in cultured cells supporting that Aβ and p-tau might have antiviral properties (7, 9) or may serve to ameliorate the consequences of neuroinflammation, amyloid and tau pathology in brain might serve to limit detrimental effects of viral exposure. Therefore, caution might be needed to pursue strategies to reduce or even eliminate hyperphosphorylated tau (14–16) and amyloid pathology (17–19). These therapeutic strategies might make the brain more susceptible to subsequent viral infection or viral recrudescence.

The insoluble/soluble p-tau ratio in cortex, a measure of p-tau pathology, was lower in KUNV- than PBS-treated NL-G-F/E4 mice, and there was a trend towards a lower insoluble/soluble p-tau ratio in KUNV- than PBS-treated NL-G-F/E3 mice. The same pattern was seen for cortical IFN-γ transcript levels and cortical IFN-γ transcript levels by viral load, with more profound increases in IFN-γ transcript levels in KUNV- than PBS-treated NL-G-F/E4 than NL-G-F/E3 mice. Consistent with this pattern, viral expression of IFN-γ in the hippocampus of the 3xTgAD model reduced p-tau pathology (53). No difference in the insoluble/soluble p-tau ratio in the cortex was seen in KUNV- compared with PBS-treated NL-G-F/E2, E3, or E4 mice, illustrating the combined NL-G-F- and apoE isoform-dependency of this pattern of p-tau pathology and IFN-γ transcript levels in cortex.

In analyses 1 and 2, following KUNV exposure, IFN-γ transcript levels were higher in E4, but not E3, mice with viral loads than those without detectable viral load, whereas TCR-α transcript levels were lower in E3, but not E4, mice with viral load than those without viral loads. The same pattern was seen in NL-G-F/apoE mice with increased IFN-γ transcript levels following KUNV exposure in NL-G-F/E4 mice. With regard to cortical IFN-γ transcripts, these results with increased IFN-γ transcript levels seen in the presence of E4 are consistent with the role of IFN-γ in driving neuroinflammation and neurodegeneration (54). Reduced TCR-α transcript levels, as seen in E3 mice, would be expected to reduce inflammation. TCR-α−expressing T cells move from the blood into the brain where they drive neuroinflammation (55, 56). Enhanced IFN-γ transcript levels in E4 and reduced TCR-α transcript levels in E3 mice are consistent with enhanced viral replication in E4 than E3 carriers seen following HSV (25) and HIV-1 (26). At 7 weeks after KUNV exposure, we did not detect genotype differences in cortical viral loads comparing NL-G-F/apoE3 and NL-G-F/E4 mice or E3 and E4 mice. It is conceivable that higher viral loads in E4-carrying mice might be present at 1 week following viral exposure, and future studies are warranted this. In addition, we recognize that the limitations of looking at only three immune measures and analyzing for a large battery of immune measure would allow to distinguish the apoE modulatory role in the immune response following viral exposure.

In analysis 2, spontaneous alternation was lower in KUNV- than PBS-treated NL-G-F/E3 and NL-G-F/E4 mice. This was not seen in NL-G-F/E2 mice, and there was even a trend to enhanced spontaneous alternation in NL-G-F/E2 mice. These data are consistent with a neuroprotective effect of E2 as compared with E3 and E4 (57).

With regard to the direction of the body temperature alterations in KUNV-treated as compared with PBS-treated mice, there was a decrease in NL-G-F/E3 mice. This might relate to hypothermia seen sometimes in WNV-associated meningitis encephalitis (58). This pattern in body temperatures was not seen in KUNV-treated NL-G-F/E2, NL-G-F/E4, or E4 mice. These data suggest that in patients with MCI and AD, there might be apoE isoform-dependent responses in body temperature following viral exposures.

In summary, there are apoE isoform-dependent responses to KUNV exposure on various measures already seen at 6 months of age and are mostly more profound in the absence than in the presence of NL-G-F. One week following exposure, cortical insoluble p-tau levels were lower in KUNV- than PBS-treated apoE, but not NL-G-F/apoE mice. Soluble p-tau levels in cortex were higher in KUNV- than PBS-treated mice, and this effect was more pronounced in NL-G-F/apoE than apoE mice. The insoluble/soluble p-tau ratio was lower in KUNV- than PBS-treated mice, and this effect was more pronounced in apoE than NL-G-F/apoE. These results are consistent with results in cultured cells supporting that Aβ and p-tau might have antiviral properties (7, 9). Future efforts are warranted to assess the early effects of KUNV on behavioral, immune, and pathological measures, to determine the pathways underlying these apoE isoform-dependent responses, whether these effects are limited to KUNV or are also seen following exposure to other neurotropic viruses, and whether susceptibility to viral exposures is increased following reduction of tau and/or amyloid pathology.

## Data Availability

The original contributions presented in the study are included in the article/**Supplementary Material**. Further inquiries can be directed to the corresponding author.
