# Supplementary material for: Effects of West Nile Virus on behavioral and cognitive performance, circadian body temperatures, cortical Aβ and p-tau pathology, and immune measures in human apoE and human amyloid precursor protein NL-G-F/human apoE mice
Source: Front Immunol. 2026 Oct 2;17:1899626. doi: 10.3389/fimmu.2026.1899626 (PMC13671938; doi:10.3389/fimmu.2026.1899626)
Supplement: Supplementary file 1 [file SupplementaryFile1.docx]

Supplementary text, figures, and tables for “Effects of West Nile Virus on behavioral and cognitive performance, circadian body temperatures, cortical Ab pathology and immune measures in human apoE and human amyloid precursor protein NL-G-F/human apoE mice”

Emily Bunnell^1#^, Elizabeth Saltonstall^1#^, Ella Shanava^1^, Abigail O’Neil^1^, Alexandra Pederson^1^, Shriya Marla^1^, Mariana Lopes Soares Llamas^1^, Isha Garg^1^, Mitali, Chaudhari^1^, Saanvi Vodapally^1^, Henry F Harrison^2^, Christopher J. Parkins^2^, Alec Hirsch^2^, Jacob Raber*^1,3,4,5^

*Activity levels in the open field*

*Analysis 1*

When the activity levels of NL-G-F/E2, NL-G-F/E3, and NL-G-F/E4 mice in the open field were analyzed (Figs. 2A-2C), there was a day x sex interaction (*F*(1,72) = 13.709, *p* < 0.001), and a trend towards an effect of sex (*F*(1,72) = 3.218, *p* = 0.077). In females, there was an effect of day (*F*(1,14) = 50.708, *p* < 0.001).

When activity levels of E3 and E4 mice were analyzed in the open field, there was an effect of sex (*F*(1,44) = 28.618, *p* < 0.001), and a day x sex interaction (*F*(1,44) = 6.922, *p* = 0.012).

*Analysis 2*

When the activity levels of NL-G-F/E2, NL-G-F/E3, and NL-G-F/E4 mice in the open field were analyzed, there was an effect of sex (*F*(1,127) = 4.789, *p* = 0.030), with females being more active than males, and a day x sex interaction (*F*(1,127) = 16.756, *p* < 0.001).

In males (Figs. 4A-4C), there was a trend towards a day x genotype x treatment interaction (*F*(2,50) = 2.634, *p* = 0.082). In NL-G-F/E2 males, there was only an effect of day (*F*(1,16) = 21.71, *p* = 0.0003) (Fig. 4A). In NL-G-F/E3 males, there was a day x treatment interaction (*F*(1,12) = 5.746, *p* = 0.0337) (Fig. 4B). In NL-G-F/E4 males, there was a trend towards an effect of day (*F*(1,22) = 3.060, *p* = 0.0942) (Fig. 4C).

In NL-G-F/E2 females (Fig. 4D), there was only an effect of day (*F*(1,14) = 39.84, *p* < 0.0001). In NL-G-F/E3 females, there was also only an effect of day (*F*(1,20) = 18.53, *p* = 0.0003) (Fig. 4E). In NL-G-F/E4 females, there was also only an effect of day (*F*(1,39) = 68.68, *p* < 0.0001) (Fig. 4F).

When the activity levels of NL-G-F/E3 and NL-G-F/E4 mice in the open field were analyzed, there was a day x sex interaction (*F*(1,95) = 10.511, *p* = 0.002), and a trend towards an effect of sex (*F*(1,95) = 2.804, *p* = 0.097).

In NL-G-F/E2 mice, there was an effect of day (*F*(1,31) = 61.474, *p* < 0.001). In NL-G-F/E3 mice, there was an effect of sex (*F*(1,32) = 5.150, *p* = 0.030), a day x sex interaction (*F*(1,32) = 4.674, *p* = 0.038), and a trend towards a day x age interaction (*F*(1,32) = 2.99, *p* = 0.093). In males, there was a day x treatment interaction (*F*(1,12) = 5.746, *p* = 0.034), with a more profound effect of treatment on day 1 than day 2; higher activity levels in KUNV- than PBS-treated NL-G-F/E3 mice, but lower activity levels in KUNV- than PBS-treated NL-G-F/E4 mice, with comparable activity levels in KUNV- and PBS-treated NL-G-F/E2 mice. In females, there was only an effect of day (*F*(1,20) = 18.528, *p* < 0.001). In NL-G-F/E4 mice, there was a day x sex interaction (*F*(1,62) = 8.290, *p* = 0.005), with more profound lower activity levels on day 2 than day 1 in females than males, and a trend towards a day x treatment interaction (*F*(1,62) = 3.598, *p* = 0.063). In males, there was a trend towards an effect of day (*F*(1,22) = 3.060, *p* = 0.094). In females, there was an effect of age (*F*(1,38) = 7.518, *p* = 0.009), with higher activity levels in old (mean: day 1: 1,856; day 2: 1,384) than young (mean: day 1: 1,583; day 2: 1,070) mice.

When activity levels of E3 and E4 mice in the open field were analyzed, there was an effect of sex (*F*(1,68) = 30.059, *p* < 0.001), with higher activity levels in females than males, day (*F*(1,68) = 11.198, *p* = 0.001), and a trend towards a day x genotype interaction (*F*(1,68) = 3.337, *p* = 0.076).

In E3 mice, there was an effect of sex (*F*(1,24) = 6.636, *p* = 0.017), with higher activity levels in females than males (Fig. S1A, S1C). In males, there was an effect of day (*F*(1,11) = 17.013, *p* = 0.002). In females, there was an effect of day (*F*(1,11) = 36.136, *p* < 0.001), a day x age interaction (*F*(1,11) = 7.307, *p* = 0.021), and a trend towards a day x treatment interaction (*F*(1,11) = 4.239, *p* = 0.064). In E4 mice, there was an effect of sex (*F*(1,42) = 24.538, *p* < 0.001), with higher activity levels in females than males (Fig. S1B, S1D) and a trend towards a day x age interaction (*F*(1,42) = 3.801, *p* = 0.058).

In males, there was a day x age interaction (*F*(1,26) = 5.669, *p* = 0.025). In E3 males, there was an effect of day (*F*(1,11) = 17.01, *p* = 0.0017) (Fig. S1A). In E4 males, there was also only an effect of day (*F*(1,27) = 59.06, *p* < 0.0001) (Fig. S1B).

In females, there was an effect of day (*F*(1,14) = 5.511, *p* = 0.034). In E3 females, there was an effect of day (*F*(1,12) = 79.22, *p* < 0.0001) (Fig. S1C). In E4 females, there also was only an effect of day (*F*(1,15) = 53.31, *p* < 0.0001) (Fig. S1D).

*3.2.2. Time spent in the center of the open field*

*Analysis 1*

In NL-G-F/E2, NL-G-F/E3, and NL-G-F/E4 mice (Figs. 2G-2H), there was an effect of day (*F*(1,73) = 27.684, *p* < 0.001), and a day x treatment interaction (*F*(1,73) = 4.092, *p* = 0.047), with a more pronounced effect of treatment on day 1 than day 2; less time spent in the center of the open field in KUNV- than PBS-treated NL-G-F/E2 and NL-G-F/E4 mice, with comparable time spent in the center of KUNV- and PBS-treated NL-G-F/E3 mice.

In E3 and E4 mice, there was a trend towards a day x genotype interaction (*F*(1,45) = 3.374, *p* = 0.073).

*Analysis 2*

When time spent in the center of the open field in NL-G-F/E2, NL-G-F/E3, and NL-G-F/E4 mice was analyzed, there was an effect of day (*F*(1,126) = 21.172, *p* < 0.001), a day x age interaction (*F*(1,126) = 10.365, *p* = 0.002), and a day x treatment interaction (*F*(1,126) = 4.267, *p* = 0.041), with a more pronounced effect of treatment on day 1 than day 2; less time spent in the center of the open field in KUNV- than PBS-treated NL-G-F/E2 and NL-G-F/E4 mice, with comparable time spent in the center of KUNV- and PBS-treated NL-G-F/E3 mice.

In NL-G-F/E3 mice, there was an effect of day (*F*(1,34) = 7.774, *p* = 0.009) (Fig. 2K). In NL-G-F/E4 mice, there was an effect of day (*F*(1,61) = 14.294, *p* < 0.001), a day x age interaction (*F*(1,61) = 5.063, *p* = 0.028), and a trend towards a day x treatment interaction (*F*(1,61) = 2.901, *p* = 0.094) (Fig. 2L).

When time spent in the center of the open field on the first day of testing of NL-G-F/E3 and NL-G-F/E4 mice was analyzed (Figs. 2K, 2L), there was an effect of sex (*F*(1,96) = 5.474, *p* = 0.021).

NL-G-F/E4 mice, there was an effect of sex (*F*(1,62) = 6.569, *p* = 0.013), with KUNV-treated mice spending less time in the center than PBS-treated mice.

When time spent in the center of the open field of E3 and E4 mice was analyzed (Figs.3G, 3H), there was an effect of day (*F*(1,67) = 7.148, *p* = 0.009) and a trend towards a day x genotype interaction (*F*(1,67) = 3.844, *p* = 0.054).

When time spent in the center of the open field on the first day of testing of E3 and E4 mice was analyzed, there was an effect of day (*F*(1,67) = 7.148, *p* = 0.009) and a trend towards a day x genotype interaction (*F*(1,67) = 3.844, *p* = 0.054) (Figs. 3G, 3H).

*Activity levels in the open field containing objects*

*Analysis 1*

In NL-G-F/E2, NL-G-F/E3, and NL-G-F/E4 mice, there was a trend towards an effect of sex (*F*(1,32) = 3.473, *p* = 0.072) (Fig. S2A-C).

When activity in the open field in the presence of the two objects of E3 and E4 mice was analyzed, there was an effect of day (*F*(1,15) = 15.451, *p* = 0.001).

*Analysis 2*

When activity in the open field in the presence of the two objects of NL-G-F/E2, NL-G-F/E3, and NL-G-F/E4 mice was analyzed, there was a day x age interaction (*F*(1,125) = 9.927, *p* = 0.002), a day x genotype interaction (*F*(2,125) = 4.739, *p* = 0.010), and a trend towards a day x genotype x treatment interaction (*F*(2,125) = 2.415, *p* = 0.094).

In NL-G-F/E2 mice, there was a day x treatment interaction (*F*(1,31) = 4.768, *p* = 0.037), with lower activity levels in PBS-treated mice on day 2 than day 1 but comparable activity levels in KUNV-treated mice (Fig. S2D). In NL-G-F/E3 mice, there were no significant effects (Fig. S2E). In NL-G-F/E4 mice, there was an effect of day (*F*(1,60) = 4.778, *p* = 0.033), a day x age interaction (*F*(1,60) = 15.680, *p* < 0.001), and a trend towards an effect of age (*F*(1,60) = 3.270, *p* = 0.076) (Fig. S2F).

When activity in the open field in the presence of the two objects in E3 and E4 mice was analyzed, there was an effect of day (*F*(1,68) = 33.480, *p* < 0.001) and a day x genotype x treatment interaction (*F*(1,68) = 4.433, *p* = 0.039), with higher activity levels in on day 1 than day 2 in PBS- than KUNV-treated E3 mice; in contrast, in E4 mice the activity levels on both days showed in similar patterns in PBS- and KUNV-treated mice (Fig. 5C, 5D).

In E3 mice, there was an effect of day (*F*(1,24) = 12.100, *p* = 0.002), a day x age interaction (*F*(1,24) = 6.150, *p* = 0.021), and a trend towards an effect of age (*F*(1,24) = 3.973, *p* = 0.058). In E4 mice, there was an effect of age (*F*(1,43) = 28.855, *p* < 0.001).

*3.2.4. Time spent in the center of the open field containing objects*

*Analysis 1*

In NL-G-F/E2, NL-G-F/E3, and NL-G-F/E4 mice, there was a trend towards a day x genotype x treatment interaction (*F*(2,72) = 2.958, *p* = 0.058).

In NL-G-F/E2 mice, there were no significant effects (Fig. S2H). In NL-G-F/E3 mice, there were no significant effects (Fig. S2I). In NL-G-F/E4 mice, there was a day x treatment interaction (*F*(1,33) = 4.969, *p* = 0.033) (Fig. S2I), with lower activity levels on day 2 than day 1 in KUNV-treated mice but comparable activity levels on both days in PBS-treated mice.

In E3 and E4 mice, there was a trend towards a day x genotype x treatment interaction (*F*(1,43) = 3.420, *p* = 0.071) (Figs. 5E, 5F), with a trend towards PBS-treated E3 and KUNV-treated E4 mice spending less time in the center of the open field containing objects on day 2 than day 1, while KUNV-treated E3 mice and PBS-treated E4 mice spent comparable times in the center of the open field containing objects on both days.

*Analysis 2*

When time spent in the center of the open field in the presence of the two objects of NL-G-F/E2, NL-G-F/E3, and NL-G-F/E4 mice was analyzed (Figs. S2J-S2L), there was an effect of age (*F*(1,125) = 33.350, *p* < 0.001), and a trend towards a day x genotype interaction (*F*(2,125) = 2.886, *p* = 0.060).

In NL-G-F/E2 mice, there was an effect of age (*F*(1,29) = 12.994, *p* = 0.001), with older mice (mean: day 53.9 sec ; day 2: 53.3 sec) spending more time in the center than younger (mean: day 1: 13.1 sec ; day 2: 16.8 sec) mice, an effect of sex (*F*(1,29) = 4.416, *p* = 0.044), a trend towards an effect of day (*F*(1,29) = 3.609, *p* = 0.067), and a trend towards a day x sex interaction (*F*(1,29) = 3.601, *p* = 0.068). In males, there was an effect of age (*F*(1,15) = 12.447, *p* = 0.003).

When time in the center of the open field in the presence of objects in E3 and E4 mice was analyzed, there were no significant effects. In E3 mice, there was an effect of day (*F*(1,25) = 46.969, *p* < 0.001), with mice spending less time in the center of the open field on day 2 than day 1. In E4 mice, there were no significant effects.

*3.2.5. Time spent exploring objects on the object recognition test day*

*Analysis 2*

When time spent exploring the objects on the object recognition test day in NL-G-F/E2, NL-G-F/E3, and NL-G-F/E4 mice was analyzed, there was an effect of age (*F*(1,126) = 26.654, *p* < 0.001), with younger mice (mean: 18.8 sec) spending less time exploring objects than older mice (37.1 sec).

When time spent exploring the objects on the object recognition test day in NL-G-F/E3 and NL-G-F/E4 mice was analyzed, there was an effect of age (*F*(1,96) = 15.486, *p* < 0.001), with younger mice spending less time exploring objects than older mice.

In NL-G-F/E2 mice, there was an effect of age (*F*(1,30) = 22.772, *p* < 0.001), with older mice (mean: 24.4 sec) exploring more than younger mice (mean: 6.2 sec). In NL-G-F/E3 mice, there also was an effect of age (*F*(1,33) = 8.177, *p* = 0.007), with older mice (mean: 35.7 sec) exploring more than younger mice (mean: 21.1 sec). In NL-G-F/E4 mice, there was an effect of age (*F*(1,61) = 10.052, *p* = 0.002), with older mice (mean: 42.5 sec) exploring more than younger mice (mean: 25.2 sec).

*3.2.6. Discrimination index*

*Analysis 1*

When the discrimination index in the object recognition test in NL-G-F/E2, NL-G-F/E3, and NL-G-F/E4 mice was analyzed, there were no significant effects (Figs. S3G-S3H).

When the discrimination index in young E3 and E4 mice was analyzed, there was a trend towards an effect of sex (*F*(1,47) = 3.108, *p* = 0.085) (Figs. S3E, S3F).

*Analysis 2*

When the discrimination index in the object recognition test in NL-G-F/E2, NL-G-F/E3, and NL-G-F/E4 mice was analyzed, there were no significant effects (Figs. S3G-S3I).

When the discrimination index in the object recognition test NL-G-F/E3 and NL-G-F/E4 mice was analyzed, there were no significant effects.

In NL-G-F/E2 mice, there was a trend towards an effect of sex (*F*(1,29) = 3.913, *p* = 0.057). In NL-G-F/E3 mice, there were no significant effects. In NL-G-F/E4 mice, there was an effect of age (*F*(1,61) = 4.905, *p* = 0.031), with a smaller discrimination index in old (mean: -0.98) than young (mean: 0.032) mice. In young mice, there were no significant effects. In older mice, there was a trend towards an effect of sex (*F*(1,26) = 3.997, *p* = 0.057).

When the discrimination index in the object recognition test in E3 and E4 mice was analyzed, there were no significant effects.

In E3 mice, there were no significant effects (Fig. S4G). In E4 mice, there was a trend towards an effect of sex (*F*(1,41) = 3.715, *p* = 0.061) (Fig. S4H).

*Regular Y maze*

*3.5.1. Arm entries in the regular Y maze*

*Analysis 1*

In NL-G-F/E2, NL-G-F/E3, and NL-G-F/E4 mice (Figs. 6A-6C), there was an effect of sex (*F*(1,44) = 8.043, *p* = 0.007), with higher activity levels in females than males.

In E3 and E4 mice (Figs. 7A, 7B), there was an effect of sex (*F*(1,44) = 8.043, *p* = 0.007).

*Analysis 2*

When the number of entries was analyzed as activity measure in NL-G-F/E2, NL-G-F/E3, and NL-G-F/E4 mice (Figs. 6D-6F), there was a trend towards an effect of sex (*F*(1,127) = 3.541, *p* = 0.062).

When the number of entries was analyzed as activity measure in NL-G-F/E3 and NL-G-F/E4 mice, there was an effect of sex (*F*(1,96) = 4.697, *p* = 0.033).

In NL-G-F/E2 mice, there were no significant effects. In NL-G-F/E3 mice, there was an effect of sex (*F*(1,33) = 4.503, *p* = 0.041). In NL-G-F/E4 mice, there was an effect of age (*F*(1,62) = 8.206, *p* = 0.006), with more entries in the older (mean: 23.2 entries) than younger (mean: 26.6 entries) mice.

When the number of entries was analyzed as activity measure in E3 and E4 mice (Figs. 7C, 7D), there was an effect of sex (*F*(1,68) = 12.418, *p* < 0.001). In E3 mice, there were no significant effects. In E4 mice, there was an effect of sex (*F*(1,42) = 12.115, *p* = 0.001).

*3.5.2. Spontaneous alternation in the regular Y maze*

*Analysis 2*

In NL-G-F/E2 mice, there was an effect of age (*F*(1,29) = 8.660, *p* = 0.006), with a higher percent spontaneous alternation in older (mean: 74.9) than younger (mean: 61.7) mice. In NL-G-F/E3 mice, there was a trend towards an effect of age (*F*(1,33) = 3.706, *p* = 0.063).

When percent spontaneous alternation in E3 and E4 mice was analyzed, there was an effect of age (*F*(1,67) = 6.770, *p* = 0.011), with a higher percent spontaneous alternation in older (mean: 69.2) than younger (mean: 61.5) mice.

In E4 mice, there was a trend towards an effect of age (*F*(1,42) = 2.936, *p* = 0.094) (Fig. 7H).

*Circadian body temperatures*

*Analysis 1*

*Week 1*

In NL-G-F/apoE mice during the dark periods, there was an effect of sex (*F*(1,39) = 4.294, *p =* 0.045), and a trend towards a day x sex interaction (*F*(2.470,96.312) = 2.488, *p =* 0.076, Greenhous-Geisser correction) (Fig. 9A, C, E). In NL-G-F/apoE mice during the light periods, there was an effect of day (*F*(2.750,107.256) = 3.588, *p =* 0.019, Greenhous-Geisser correction), sex (*F*(1,39) = 4.461, *p =* 0.041), and a trend towards a treatment x genotype interaction (Fig. 9A, C, E).

In NL-G-F/E4 mice during the dark periods, there was an effect of sex (*F*(1,17) = 8.067, *p =* 0.011) (Fig. 9E), with higher body temperatures in females than males (males: 35.94 ± 0.11^0^C; females: 36.50 ± 0.15^0^C). In NL-G-F/E4 mice during the light periods, there also was an effect of sex (*F*(1,17) = 7.640, *p =* 0.013) (Fig. 9E), with higher body temperatures in females than males (males: 35.40 ± 0.09^0^C; females: 35.86 ± 0.12^0^C).

In E4 mice during the dark periods, there also was an effect of sex (*F*(1,12) = 12.515, *p =* 0.004) (Fig. 9G), with higher body temperatures in females than males (males: 35.94 ± 0.13^0^C; females: 36.47 ± 0.09^0^C). In E4 mice during the light periods, there also was an effect of sex (*F*(1,12) = 30.309, *p <* 0.001) (Fig. 9G), with higher body temperatures in females than males (males: 35.09 ± 0.10^0^C; females: 35.91 ± 0.09^0^C).

Next, the body temperatures during the dark periods in NL-G-F/E4 and E4 mice were compared. There was only an effect of sex (*F*(1,30) = 19.124, *p <* 0.001). When the body temperatures during the light periods in NL-G-F/E4 and E4 mice were compared, there was an effect of day (*F*(3.154,94.626) = 4.146, *p =* 0.007, Greenhous-Geisser correction) and an effect of sex (*F*(1,30) = 28.212, *p <* 0.001).

*Week 2*

In NL-G-F/apoE mice during the dark periods, there was an effect of sex (*F*(1,38) = 11.579, *p =* 0.002), and a trend towards an effect of night (*F*(4.463,177.210) = 2.168, *p =* 0.064, Greenhous-Geisser correction) (Fig. 9B, D, F). In NL-G-F/apoE mice during the light periods, there was a trend towards an effect of day (*F*(4.435,168.542) = 2.301, *p =* 0.054, Greenhous-Geisser correction), a trend towards an effect of a day x sex interaction (*F*(4.435,168.542) = 2.319, *p =* 0.053, Greenhous-Geisser correction), and a trend towards a day x genotype interaction (*F*(8.871,168.542) = 1.807, *p =* 0.071, Greenhous-Geisser correction) (Fig. 9B, D, F).

In NL-G-F/E2 mice during the dark periods, there were no significant effects (Fig. 9B). In NL-G-F/E2 mice during the light periods, there was a trend towards a day x treatment interaction (*F*(7,56) = 1.968, *p =* 0.076) (Fig. 9B).

In NL-G-F/E3 mice during the dark periods, there was a trend towards an effect of sex (*F*(1,12) = 3.560, *p =* 0.084), a trend towards an effect of night (*F*(3.367,40.402) = 2.709, *p =* 0.052, Greenhous-Geisser correction) and a trend towards a night x sex interaction (*F*(3.367,40.402) = 2.709, *p =* 0.084, Greenhous-Geisser correction) (Fig. 9D). In NL-G-F/E3 mice during the light periods, there was an effect of sex (*F*(1,12) = 5.601, *p =* 0.036), with higher body temperatures in females than males (males: 35.24 ± 0.17^0^C; females: 36.67 ± 0.10^0^C), and a trend towards a day x treatment interaction (*F*(2.263,27.154) = 2.715, *p =* 0.078, Greenhous-Geisser correction) (Fig. 9D).

In NL-G-F/E4 mice during the dark periods, there was an effect of sex (*F*(1,17) = 8.725, *p <* 0.009) (Fig. 9F), with higher body temperatures in females than males (males: 35.98 ± 0.09 ^0^C; females: 36.49 ± 0.14^0^C). In NL-G-F/E4 mice during the light periods, there also was an effect of sex (*F*(1,17) = 15.236, *p =* 0.001) (Fig. 9F), with higher body temperatures in females than males (males: 35.22 ± 0.08^0^C; females: 35.77 ± 0.11^0^C).

In E4 mice during the dark periods, there was an effect of sex (*F*(1,12) = 8.593, *p =* 0.013) (Fig. 9H), with higher body temperatures in females than males (males: 35.93 ± 0.16^0^C; females: 36.58 ± 0.15^0^C). In E4 mice during the light periods, there also was an effect of sex (*F*(1,12) = 19.141, *p <* 0.001) (Fig. 9H), with higher body temperatures in females than males (males: 35.00 ± 0.14^0^C; females: 35.97 ± 0.17^0^C).

When the body temperatures during the dark periods in NL-G-F/E4 and E4 mice were compared, there were no significant effects. When the body temperatures during the light periods in NL-G-F/E4 and E4 mice were compared, there was an effect of sex (*F*(1,30) = 33.160, *p <* 0.001) and a day by NL-G-F interaction (*F*(3.852,115.573) = 8.763, *p <* 0.001, Greenhous-Geisser correction). However, on no day was there a genotype difference in body temperatures (Table S1).

*Week 4 (S6)*

In NL-G-F/E4 mice during the dark periods, there were no significant effects for body temperatures. In NL-G-F/E4 mice during the light periods, there was an effect of day (*F*(2.390,19.118) = 23.626, *p <* 0.001, Greenhouse-Geisser) and a day x sex interaction (*F*(2.390,19.118) = 14.514, *p <* 0.001, Greenhouse-Geisser). There was a trend towards a sex difference with higher body temperatures in females than males on day 3 (males: 35.04 ± 0.10^0^C; females: 35.55 ± 0.25^0^C; *p* = 0.073, 2-tailed t test) and day 4 (males: 35.31 ± 0.12^0^C; females: 35.73 ± 0.20^0^C; *p* = 0.096, 2-tailed t test).

In E4 mice during the dark periods, there was an effect of sex (*F*(1,12) = 4.806, *p =* 0.049) for body temperatures, with higher body temperatures in females (35.52 ± 0.17^0^C) than males (35.18 ± 0.12^0^C). In E4 mice during the light periods, there was an effect of sex (*F*(1,12) = 9.795, *p =* 0.009), with higher body temperature in females (35.18 ± 0.11^0^C) than males (34.55 ± 0.12^0^C), and a day x treatment interaction (*F*(1.872,22.469) = 5.327, *p =* 0.014, Greenhouse-Geisser).

When body temperatures in NL-G-F/E4 and E4 mice during the dark periods were compared, there was an effect of sex (*F*(1,21) = 4.932, *p =* 0.037), with higher body temperatures in females (35.52 ± 0.11^0^C) than males (35.18 ± 0.08^0^C), a night x NL-G-F interaction (*F*(2.306,48.417) = 3.265, *p =* 0.040, Greenhouse-Geisser), but no effect of NL-G-F in any night, and a trend towards a night x treatment interaction (*F*(2.306,48.417) = 2.760, *p =* 0.066, Greenhouse-Geisser). When body temperatures in NL-G-F/E4 and E4 mice during the light periods were compared, there was an effect of day (*F*(2.307,48.450) = 7.666, *p <* 0.001, Greenhouse-Geisser), sex (*F*(1,21) = 6.738, *p =* 0.017), with higher body temperatures in females (35.91 ± 0.09^0^C) than males (34.55 ± 0.09^0^C), a day x sex interaction (*F*(2.307,48.450) = 4.805, *p =* 0.009, Greenhouse-Geisser), with higher body temperatures in females than males on days 1 (males: 35.17 ± 0.11^0^C; females: 35.60 ± 0.18^0^C; *p* = 0.040, 2-tailed t test), 3 (males: 35.03 ± 0.09^0^C; females: 35.61 ± 0.14^0^C; p = 0.002, 2-tailed t test), 4 (males: 35.12 ± 0.09^0^C; females: 35.79 ± 0.12^0^C; *p* < 0.001, 2-tailed t test), 5 (males: 35.28 ± 0.16^0^C; females: 35.73 ± 0.12^0^C; p = 0.034, 2-tailed t test), a day x treatment interaction (*F*(2.307,48.450) = 3.423, *p =* 0.035, Greenhouse-Geisser), with no effect of treatment on any day.

*Analysis 2*

*Week 1*

In NL-G-F/apoE mice during the dark periods, there was an effect of sex (*F*(1,88) = 29.539, *p <* 0.001), with higher body temperatures in females than males (Table S2), age (*F*(1,88) = 4.420, *p =* 0.038), a night x sex interaction (*F*(3.119,44.421) = 4.328, *p =* 0.004, Greenhouse-Geisser), and a night x genotype interaction (*F*(3.119,44.421) = 2.956, *p =* 0.007, Greenhouse-Geisser). In NL-G-F/apoE mice during the light periods, there was an effect of sex (*F*(1,88) = 41.767, *p <* 0.001), with higher body temperatures in females than males (Table S2), day (*F*(3.645,320.733) = 12.012, *p <* 0.001, Greenhouse-Geisser), a day x sex interaction (*F*(3.645,320.733) = 3.193, *p =* 0.017, Greenhouse-Geisser), a day x age interaction (*F*(3.645,320.733) = 2.595, *p =* 0.041, Greenhouse-Geisser), with a higher body temperature in older than younger mice in day 6 (Table S3), a day x genotype interaction (*F*(7.289,320.733) = 6.246, *p <* 0.001, Greenhouse-Geisser).

In NL-G-F/E2 mice during the dark periods, there was an effect of sex (*F*(1,18) = 11.212, *p =* 0.004), with higher body temperatures in females than males (males: 35.61 ± 0.11^0^C; females: 36.15 ± 0.13^0^C). In NL-G-F/E2 mice during the light periods, there was an effect of sex (*F*(1,17) = 23.726, *p <* 0.001), with higher body temperatures in females than males (males: 34.92 ± 0.11^0^C; females: 35.68 ± 0.11^0^C), and a day x age interaction (*F*(3.576,60.786) = 4.090, *p =* 0.007, Greenhouse-Geisser), with a higher body temperature in older than younger mice on days 5-8 and a lower body temperature on day 4 (Table S4).

In NL-G-F/E3 mice during the dark periods, there was a trend towards a night x age interaction (*F*(2.125,50.988) = 3.082, *p =* 0.052, Greenhouse-Geisser). In NL-G-F/E3 mice during the light periods, there was an effect of day (*F*(2.367,56.819) = 7.983, *p <* 0.001, Greenhouse-Geisser) and a day x age interaction (*F*(2.367,56.819) = 2.554, *p =* 0.016, Greenhouse-Geisser), with a lower body temperature in older than younger mice on day 1 (Table S5).

In NL-G-F/E4 mice during the dark periods, there was an effect of sex (*F*(1,44) = 24.959, *p <* 0.001), with higher body temperatures in females than males (males: 35.95 ± 0.08^0^C; females: 36.78 ± 0.08^0^C), an effect of age (*F*(1,24) = 4.537, *p =* 0.039), with higher activity levels in older than younger mice (younger: 36.19 ± 0.11^0^C; older: 36.76 ± 0.10^0^C), an effect of night (*F*(3.091,135.984) = 4.328, *p =* 0.006, Greenhouse-Geisser), and a trend towards a night x sex interaction (*F*(3.091,135.984) = 2.326, *p =* 0.076, Greenhouse-Geisser). In NL-G-F/E4 mice during the light periods, there was an effect of sex (*F*(1,44) = 21.359, *p <* 0.001), day (*F*(4.069,179.019) = 17.742, *p <* 0.001, Greenhouse-Geisser), with higher body temperatures in females than males (males: 35.43 ± 0.07^0^C; females: 35.98 ± 0.06^0^C), a day x age interaction (*F*(4.069,179.019) = 4.045, *p =* 0.003, Greenhouse-Geisser), with higher body temperatures in older than younger mice on days 2-8 (Table S6), a day x sex interaction (*F*(4.069,179.019) = 3.469, *p =* 0.001, Greenhouse-Geisser), with higher body temperatures in females than males on all days (Table S7), and a trend towards an effect of age (*F*(1,44) = 2.869, *p =* 0.097).

In E4 mice during the dark periods, there was an effect of sex (*F*(1,26) = 37.688, *p <* 0.001), with higher body temperatures in females than males (males: 35.68 ± 0.09^0^C; females: 36.32 ± 0.09^0^C), and age (*F*(1,26) = 15.823, *p <* 0.001), with lower activity levels in older than younger mice (younger: 36.19 ± 0.10^0^C; older: 35.69 ± 0.10^0^C). In E4 mice during the light periods, there was an effect of sex (*F*(1,26) = 79.826, *p <* 0.001), age (*F*(1,26) = 7.811, *p <* 0.010), with lower activity levels in older than younger mice (younger: 35.47 ± 0.13^0^C; older: 35.08 ± 0.12^0^C), with higher body temperatures in females than males (males: 34.94 ± 0.06^0^C; females: 35.78 ± 0.09^0^C), and a trend towards an effect of treatment (*F*(1,26) = 3.241, *p =* 0.083).

When the body temperatures in the dark periods in the NL-G-F/E4 and E4 mice were compared, there was an effect of sex (*F*(1,72) = 60.969, *p <* 0.001), NL-G-F (*F*(1,72) = 16.419, *p =* 0.023), night (*F*(4.267,307.199) = 3.293, *p =* 0.010, Greenhouse-Geisser), a night x sex interaction (*F*(4.267,307.199) = 2.538, *p =* 0.037, Greenhouse-Geisser), with higher body temperatures in females than males on all days (Table S8), a night x age interaction (*F*(4.267,307.199) = 2.858, *p =* 0.021, Greenhouse-Geisser), with a trend towards a higher body temperatures in older than younger mice on days 3, 5, and 8 (Table S9). When the body temperatures in the light periods in the NL-G-F/E4 and E4 mice were compared, there was an effect of sex (*F*(1,72) = 87.148, *p <* 0.001), day (*F*(4.440,319.696) = 12.545, *p <* 0.001, Greenhouse-Geisser), a day x sex interaction (*F*(4.440,319.696) = 4.771, *p <* 0.001, Greenhouse-Geisser), and a day x age interaction (*F*(4.440,319.696) = 3.622, *p =* 0.005, Greenhouse-Geisser), with a trend towards a higher body temperature in older than younger mice on day 6 (Table S11).

*Week 2*

In NL-G-F/apoE mice during the dark periods, there was an effect of sex (*F*(1,86) = 45.381, *p <* 0.001), night (*F*(4.574,393.384) = 3.578, *p =* 0.005, Greenhouse-Geisser), a night x sex interaction (*F*(4.574,393.384) = 2.774, *p =* 0.021, Greenhouse-Geisser), with higher body temperatures in females than males on all days (Table S13), a night x treatment interaction (*F*(4.574,393.384) = 2.434, *p =* 0.039, Greenhouse-Geisser) but there was no effect of treatment in any night (Table S14), a night x treatment x genotype interaction (*F*(9.184,393.384) = 2.353, *p =* 0.013, Greenhouse-Geisser), and a trend towards an effect of age (*F*(1,86) = 3.899, *p =* 0.052), and a trend towards a night x age interaction (*F*(4.574,393.384) = 2.115, *p =* 0.069, Greenhouse-Geisser). In NL-G-F/apoE mice during the light periods, there was an effect of day (*F*(4.241,364.722) = 3.827, *p =* 0.004, Greenhouse-Geisser), a day x sex interaction (*F*(4.241,364.722) = 4.808, *p <* 0.001, Greenhouse-Geisser) (Table S15), and a day x age interaction (*F*(4.241,364.722) = 3.827, *p =* 0.004, Greenhouse-Geisser), with higher body temperatures in older than younger mice on days 3 and 4 and a trend towards higher body temperatures in older than younger mice on day 5 (Table S16).

In NL-G-F/E2 mice during the dark periods, there was an effect of sex (*F*(1,18) = 21.877, *p <* 0.001), with higher body temperatures in females than males (males: 35.57 ± 0.14^0^C; females: 36.15 ± 0.11^0^C). In NL-G-F/E2 mice during the light periods, there was an effect of sex (*F*(1,17) = 34.826, *p <* 0.001), with higher body temperatures in females than males (males: 34.77 ± 0.12^0^C; females: 35.77 ± 0.10^0^C), and a trend towards an effect of age (*F*(1,17) = 3.374, *p =* 0.084).

In NL-G-F/E3 mice during the dark periods, there was an effect of night (*F*(7,147) = 5.782, *p <* 0.001), a night x age interaction (*F*(7,147) = 2.889, *p =* 0.007), with lower body temperatures in older than younger mice on days 5-6 and a trend towards lower body temperatures in older than younger mice on days 2-4 (Table S18), and a night x sex interaction (*F*(7,147) = 4.516, *p <* 0.001) (Table S19). In NL-G-F/E3 mice during the light periods, there was an effect of sex (*F*(1,22) = 6.560, *p =* 0.018), with higher body temperatures in females than males (males: 35.27 ± 0.11^0^C; females: 35.72 ± 0.09^0^C), and day x treatment interaction (*F*(3.702,81.441) = 3.742, *p =* 0.009, Greenhouse-Geisser), with lower body temperatures in KUNV- than PBS-treated mice on days 4 and 6 and a trend towards lower body temperatures in KUNV- than PBS-treated mice on day 5 (Table S21).

In NL-G-F/E4 mice during the dark periods, there was an effect of sex (*F*(1,44) = 25.589, *p <* 0.001), with higher body temperatures in females than males (males: 35.97 ± 0.07^0^C; females: 36.84 ± 0.09^0^C), and an effect of age (*F*(1,44) = 5.718, *p =* 0.021), with higher activity levels in older than younger mice (younger: 36.21 ± 0.10^0^C; older: 36.83 ± 0.11^0^C). In NL-G-F/E4 mice during the light periods, there was an effect of sex (*F*(1,44) = 35.289, *p <* 0.001), with higher body temperatures in females than males (males: 35.23 ± 0.06^0^C; females: 35.99 ± 0.06^0^C), age (*F*(1,44) = 4.411, *p =* 0.041), day *F*(3.427,150.796) = 2.601, *p =* 0.047, Greenhouse-Geisser), a day x sex interaction *F*(3.427,150.796) = 3.294, *p =* 0.017, Greenhouse-Geisser), with higher body temperatures in females than males on all days (Table S22), and a day x age interaction *F*(3.427,150.796) = 3.114, *p =* 0.023, Greenhouse-Geisser), with higher body temperatures in older than younger mice on all days (Table S23).

In E4 mice during the dark periods, there was an effect of sex (*F*(1,25) = 19.889, *p <* 0.001), with higher body temperatures in females than males (males: 35.73 ± 0.11^0^C; females: 36.43 ± 0.13^0^C), and age (*F*(1,25) = 4.587, *p =* 0.042), with higher activity levels in older than younger mice (younger: 36.23 ± 0.14^0^C; older: 35.75 ± 0.13^0^C). In E4 mice during the light periods, there was an effect of sex (*F*(1,24) = 51.310, *p <* 0.001), with higher body temperatures in females than males (males: 34.83 ± 0.10^0^C; females: 35.90 ± 0.14^0^C), day (*F*(3.163,75.915) = 3.087, *p =* 0.030, Greenhouse-Geisser), and a day x age interaction *F*(3.163,75.915) = 4.448, *p =* 0.005, Greenhouse-Geisser).

When body temperatures during the dark periods in NL-G-F/E4 and E4 mice were compared, there was an effect of night (*F*(4.323,311.222) = 4.151, *p =* 0.002, Greenhouse-Geisser), and a night x sex interaction (*F*(4.323,311.222) = 3.130, *p =* 0.013, Greenhouse-Geisser), with higher body temperatures in older than younger mice on all days (Table S24). When body temperatures during the light periods in NL-G-F/E4 and E4 mice were compared, there was an effect of sex (*F*(1,71) = 105.400, *p <* 0.001), a day x sex interaction (*F*(3.411,244.342) = 4.094, *p =* 0.005, Greenhouse-Geisser), with higher body temperatures in females than males on all days (Table S25), and a day x NL-G-F interaction (*F*(3.411,244.342) = 3.026, *p =* 0.024, Greenhouse-Geisser).

*Week 3 (Fig. S7)*

In NL-G-F/E2 mice during the dark periods, there was a trend towards an effect of sex (*F*(1,7) = 5.313, *p* = 0.055). In NL-G-F/E2 mice during the light periods, there was an effect of sex (*F*(1,7) = 13.556, *p* = 0.008), with higher body temperatures in females (35.29 ± 0.10^0^C) than males (33.94 ± 0.11^0^C), and a trend towards a day x age interaction (*F*(7,49) = 2.206, *p* = 0.050).

In E4 mice during the dark periods, there was an effect of sex (*F*(1,24) = 20.113, *p* < 0.001), with higher body temperatures in females (35.81 ± 0.10^0^C) than males (34.66 ± 0.11^0^C), and an effect of age (*F*(1,24) = 4.284, *p* = 0.049), with higher body temperatures in younger (35.15 ± 0.11^0^C) than older (34.66 ± 0.13^0^C) mice. In E4 mice during the light periods, there was an effect of sex (*F*(1,24) = 42.373, *p* < 0.001), with higher body temperatures in females (35.23 ± 0.09^0^C) than males (34.13 ± 0.09^0^C), a trend towards a day x sex interaction (*F*(2.082,49.961) = 3.098, *p =* 0.052, Greenhouse-Geisser), and a trend towards a day x age interaction (*F*(2.082,49.961) = 2.409, *p =* 0.098, Greenhouse-Geisser).

*Week 4 (Fig. S8)*

In NL-G-F/apoE mice during the dark periods, there was an effect of night (*F*(4.356,130.670) = 5.732, *p <* 0.001, Greenhouse-Geisser. In NL-G-F/apoE mice during the light periods, there was an effect of day, sex (*F*(1,29) = 2.803, *p* = 0.077), with higher body temperatures in females (34.60 ± 0.06^0^C) than males (33.95 ± 0.09^0^C), and a day x sex interaction, with a higher body temperature in females than males on each day (day 1: males: 35.15 ± 0.09^0^C; females: 35.55 ± 0.1^0^ C; *p* = 0.013, 2-tailed *t test*; day 2: males: 35.12 ± 0.08^0^C; females: 35.51 ± 0.12^0^C; *p* = 0.049, 2-tailed *t test*; day 3: males: 35.08 ± 0.07^0^C; females: 35.96 ± 0.12^0^C; *p* < 0.001, 2-tailed *t test*; day 4: males: 35.21 ± 0.07^0^C; females: 36.00 ± 0.10^0^C; *p* = 0.001, 2-tailed *t test*; day 5: males: 35.50 ± 0.11^0^C; females: 35.99 ± 0.08^0^C; *p* < 0.001, 2-tailed *t test*; day 6: males: 35.42 ± 0.10^0^C; females: 35.86 ± 0.06^0^C; *p* < 0.001, 2-tailed *t test*; day 7: males: 35.46 ± 0.10^0^C; females: 35.73 ± 0.05^0^C; *p* = 0.018, 2-tailed *t test*; day 8: males: 35.32 ± 0.10^0^C; females: 35.68 ± 0.05^0^C; *p* = 0.002, 2-tailed *t test*, a trend towards an effect of genotype (*F*(2,29) = 3.397, *p* = 0.076).

In NL-G-F/E2 mice during the dark periods, there was a a trend towards an effect of night (*F*(2.499,17.490) = 2.907, *p =* 0.072, Greenhouse-Geisser), and a trend towards a day x sex interaction (*F*(3.683,44.200) = 3.088, *p =* 0.001, Greenhouse-Geisser). In NL-G-F/E2 mice during the light periods, there was an effect of day, an effect of sex, a day x sex interaction, and a day x age interaction.

In NL-G-F/E3 mice during the dark periods, there were no significant effects. In NL-G-F/E3 mice during the light periods, there were no significant effects.

In NL-G-F/E4 mice during the dark periods, there a night x age interaction (*F*(3.683,44.200) = 5.630, *p =* 0.001, Greenhouse-Geisser), with higher body temperatures in older than younger mice on day 1 (older: 36.67 ± 0.14^0^C; younger: 36.12 ± 0.15^0^C; *p* = 0.038, 2-tailed *t test*) and days 4-8 (day 4: (older: 36.84 ± 0.11^0^C; younger: 36.20 ± 0.16^0^C; *p* = 0.002, 2-tailed *t test*); day 5: older: 36.80 ± 0.09^0^C; younger: 36.07 ± 0.14^0^C; *p* < 0.001, 2-tailed *t test*; day 6: older: 36.77 ± 0.09^0^C; younger: 36.00 ± 0.13^0^C; *p* < 0.001, 2-tailed *t test*; day 7: older: 36.48 ± 0.10^0^C; younger: 35.97 ± 0.10^0^C; *p* < 0.001, 2-tailed *t test*; day 8: older: 36.45 ± 0.11^0^C; younger: 35.94 ± 0.10^0^C; *p* = 0.001, 2-tailed *t test*), and a trend towards an effect of night (*F*(3.683,44.200) = 2.209, *p =* 0.088, Greenhouse-Geisser). In NL-G-F/E4 mice during the light periods, there was an effect of day (*F*(2.951,35.416) = 11.324, *p <* 0.001, Greenhouse-Geisser) and a day x sex interaction (*F*(2.951,35.416) = 9.258, *p <* 0.001, Greenhouse-Geisser), with higher body temperatures in females than males on day 1 (males: 35.15 ± 0.09^0^C; females: 35.55 ± 0.11^0^C; *p* = 0.013, 2-tailed *t test*), day 3: males: 35.27 ± 0.18^0^C; females: 35.31 ± 0.12^0^C; *p* < 0.001, 2-tailed *t test*; day 4: (males: 35.21 ± 0.07^0^C; females: 36.00 ± 0.10^0^C; *p* = 0.009, 2-tailed *t test*; and day 5: males: 35.50 ± 0.11^0^C; females: 35.99 ± 0.08^0^C; *p* = 0.001, 2-tailed *t test*; day 6: males: 35.42 ± 0.10^0^C; females: 35.86 ± 0.06^0^C; *p* < 0.001, 2-tailed *t test*).

In E4 mice during the dark period, there was an effect of sex (*F*(1,23) = 10.602, *p* = 0.003), with higher body temperatures in females (35.52 ± 0.14^0^C) than males (34.75 ± 0.10^0^C), a night x age interaction (*F*(2.032,46.736) = 4.916, *p =* 0.011, Greenhouse-Geisser), with lower body temperatures in older than younger mice on days 4-8 (day 4: younger: 36.10 ± 0.12^0^C; older: 35.48 ± 0.10^0^C; *p* < 0.001, 2-tailed *t test*; day 5: younger: 36.04 ± 0.11^0^C; older: 35.48 ± 0.12^0^C; *p* = 0.002, 2-tailed *t test*; day 6: younger: 36.14 ± 0.10^0^C; older: 35.50 ± 0.13^0^C; *p* < 0.001, 2-tailed *t test*; day 7: younger: 36.00 ± 0.10^0^C; older: 35.41 ± 0.11^0^C; *p* < 0.001, 2-tailed *t test*; and day 8: younger: 36.11 ± 0.10^0^C; older: 35.43 ± 0.15^0^C; *p* < 0.001, 2-tailed *t test*), and a trend towards an effect of age (*F*(1,23) = 3.983, *p* = 0.058). In E4 mice during the light periods, there was an effect of day (*F*(2.539,58.391) = 2.949, *p =* 0.048, Greenhouse-Geisser), sex (*F*(1,23) = 25.554, *p* < 0.001), with higher body temperatures in females (35.18 ± 0.09^0^C) than males (34.20 ± 0.10^0^C), age (*F*(1,23) = 6.344, *p* = 0.019), with higher body temperatures in younger (34.55 ± 0.10^0^C) than older (34.20 ± 0.12^0^C) mice, a day x treatment interaction (*F*(2.539,58.391) = 4.961, *p =* 0.006, Greenhouse-Geisser), with higher body temperatures in KUNV- than PBS-treated mice on days 1 (PBS: 34.82 ± 0.15^0^C; KUNV: 35.40 ± 0.14^0^C; *p* = 0.009, 2-tailed *t test*), 2 (PBS: 34.75 ± 0.16^0^C; KUNV: 35.36 ± 0.15^0^C; *p* = 0.009, 2-tailed *t test*), and 8 (PBS: 34.81 ± 0.18^0^C; KUNV: 35.25 ± 0.08^0^C; *p* = 0.045, 2-tailed *t test*) and a trend towards a higher body temperature in KUNV- than PBS-treated mice on day 7 (PBS: 35.08 ± 0.16^0^C; KUNV: 35.38 ± 0.08^0^C; *p* = 0.097, 2-tailed *t test*), and a trend towards an effect of treatment (*F*(1,23) = 3.532, *p* = 0.073).

Comparing body temperatures in NL-G-F/E4 and E4 mice during the dark periods, there was an effect of night (*F*(2.834,104.850) = 2.858, *p =* 0.043, Greenhouse-Geisser), sex (*F*(1,37) = 13.509, *p* < 0.001), with higher body temperatures in females (35.52 ± 0.08^0^C) than males (34.75 ± 0.07^0^C), NL-G-F (*F*(1,37) = 5.648, *p* = 0.023), a night x age interaction (*F*(2.834,104.850) = 7.828, *p <* 0.001, Greenhouse-Geisser), with no effect of age in any night, and a night x NL-G-F interaction (*F*(2.834,104.850) = 3.389, *p =* 0.023, Greenhouse-Geisser), with lower body temperatures in NL-G-F/E4 than E4 mice in all nights (day 1: E4: 36.29 ± 0.13^0^C; NL-G-F/E4: 35.80 ± 0.10^0^C; *p* = 0.004, 2-tailed *t test*; day 2: E4: 36.18 ± 0.10^0^C; NL-G-F/E4: 35.83 ± 0.11^0^C; *p* = 0.041, 2-tailed *t test*; day 3: E4: 36.20 ± 0.11^0^C; NL-G-F/E4: 35.79 ± 0.11^0^C; *p* = 0.023, 2-tailed *t test*; day 4: E4: 36.61 ± 0.11^0^C; NL-G-F/E4: 35.83 ± 0.10^0^C; *p* < 0.001, 2-tailed *t test*; day 5: E4: 36.53 ± 0.10^0^C; NL-G-F/E4: 35.80 ± 0.10^0^C; *p* < 0.001, 2-tailed *t test*; day 6: E4: 36.49 ± 0.10^0^C; NL-G-F/E4: 35.85 ± 0.10^0^C; *p* < 0.001, 2-tailed *t test*; day 7: E4: 36.27 ± 0.08^0^C; NL-G-F/E4: 35.74 ± 0.09^0^C; *p* < 0.001, 2-tailed *t test*; day 8: E4: 36.24 ± 0.08^0^C; NL-G-F/E4: 35.82 ± 0.11^0^C; *p* = 0.003, 2-tailed *t test*). Comparing body temperatures in NL-G-F/E4 and E4 mice during the light periods, there was an effect of sex (*F*(1,37) = 19.683, *p* < 0.001), with higher body temperatures in females (34.91 ± 0.07^0^C) than males (34.20 ± 0.09^0^C), day (*F*(3.114,115.8205) = 7.695, *p <* 0.001, Greenhouse-Geisser), NL-G-F (*F*(1,37) = 9.746, *p* = 0.003), a day x sex interaction (*F*(3.114,115.8205) = 5.869, *p <* 0.001, Greenhouse-Geisser), with higher body temperatures in females than males on all days (day 1: males: 35.00 ± 0.09^0^C; females: 35.62 ± 0.12^0^C; *p* < 0.001, 2-tailed *t test*; day 2: males: 34.99 ± 0.10^0^C; females: 35.47 ± 0.13^0^C; *p* = 0.005, 2-tailed *t test*; day 3: males: 34.88 ± 0.08^0^C; females: 35.79 ± 0.11^0^C; *p* < 0.001, 2-tailed *t test*; day 4: males: 34.98 ± 0.08^0^C; females: 36.08 ± 0.10^0^C; *p* < 0.001, 2-tailed *t test*; day 5: males: 35.18 ± 0.10^0^C; females: 35.99 ± 0.09^0^C; *p* < 0.001, 2-tailed *t test*; day 6: males: 35.39 ± 0.09^0^C; females: 35.97 ± 0.07^0^C; *p* < 0.001, 2-tailed *t test*; day 7: males: 35.38 ± 0.09^0^C; females: 35.80 ± 0.06^0^C; *p* < 0.001, 2-tailed *t test*; and day 8: males: 35.21 ± 0.10^0^C; females: 35.72 ± 0.06^0^C; *p* < 0.001, 2-tailed *t test*), a trend towards an effect of age (*F*(1,37) = 2.915, *p* = 0.096) and a trend towards a day x treatment interaction (*F*(3.114,115.8205) = 2.511, *p =* 0.060, Greenhouse-Geisser).

*Insoluble and soluble cortical Aβ40, Aβ42, the Aβ42/40 ratio, insoluble and soluble p-tau and the insoluble/soluble p-tau ratio*

The cortical tissues of the young mice (Analysis 1) were processed for analysis of markers of AD pathology. In NL-G-F/apoE mice, there was an effect of sex (*F*(1,56) = 4.704, *p* = 0.035), with higher levels in females (28.4 ± 6.6 pg/mg protein) than males (11.0 ± 2.3 pg/mg protein).

There was an effect of sex (*F*(1,54) = 5.796, *p* < 0.020), with higher levels in females (248.6 ± 45.9 pg/mg protein) than males (128.9 ± 27.5 pg/mg protein), for soluble cortical Aβ40 levels.

For the soluble Aβ42/40 ratio, there was only a trend towards an effect of sex (*F*(1,53) = 3.659, *p* = 0.061), with a trend towards a higher ratio in males (0.94 ± 0.19) than females (0.61 ± 0.05) (Fig. 11F).

*Immune measures in cortex*

*Analysis 1*

In NL-G-F/E3 mice, there was an effect of sex (*F*(1,11) = 10.452, *p* = 0.008), with higher TCR-α transcript levels in males (10.20 ± 3.16) than females (2.25 ± 0.63).

In apoE mice, there was an effect of sex (*F*(1,42) = 13.706, *p* < 0.001) for TCR-α transcript levels in the cortex, with higher TCR-α transcript levels in males (2.38 ± 0.36) than females (0.98 ± 0.19).

In apoE mice, there was an effect of sex (*F*(1,34) = 4.788, *p* = 0.036) for cortical TNF-α transcript levels, with higher TNF-α transcript levels in males (1.60 ± 0.18) than females (1.16 ± 0.16).

For TCR-α cortical transcript levels, there was an effect of sex (*F*(1,46) = 4.478, *p* = 0.040), with higher TCR-α transcript levels in males (2.40 ± 0.32) than females (1.35 ± 0.30). For TNF-a cortical transcript levels, there were no significant effects (Fig. 14E).

*Analysis 2*

In NL-G-F/E3 mice, there was an effect of sex (*F*(1,20) = 8.963, *p* = 0.007), with higher TCR-α transcript levels in males (8.00 ± 2.61) than females (1.63 ± 0.40).

In E4 mice, there was an effect of sex (*F*(1,32) = 8.196, *p* = 0.007), with higher TCR-α transcript levels in males (2.61 ± 0.41) than females (1.25 ± 0.23).

**Fig. S1.** Activity levels of KUNV- and PBS-treated E3 (**A**, **C**) and E4 (**B**, **D**) male (**A**, **B**) and female (**C**, **D**) mice in the open field. **A/C**. In E3 mice, there was an effect of sex (*F*(1,24) = 6.636, *p* = 0.017), with higher activity levels in females (**C**) than males (**A**). **B/D**. In E4 mice, there was an effect of sex (*F*(1,42) = 24.538, *p* < 0.001), with higher activity levels in females (**D**) than males (**B**).

**Fig. S2.** Activity levels (**A**-**F**) and time spent in the center (**G**-**L**) of KUNV- and PBS-treated NL-G-F/E2 (**A**, **D, G, J**), NL-G-F/E3 (**B**, **E**, **H**, **K**) and NL-G-F/E4 (**C**, **F**, **I**, **L**) mice in the open field containing objects. **D/E/F.** When activity in the open field in the presence of the two objects of NL-G-F/E2, NL-G-F/E3, and NL-G-F/E4 mice in Analysis 2 was analyzed, there was an effect of genotype (*F*(2,125) = 6.631, ***p* < 0.002), with NL-G-F/E3 mice (**F**) moving more than NL-G-F/E2 (*p* = 0.002, Tukey’s) (**E**) and NL-G-F/E4 (2,178) (*p* =0.02, Tukey’s) mice. **G/H/I.** When time spent exploring the objects on the object recognition test day in NL-G-F/E2, NL-G-F/E3, and NL-G-F/E4 mice was analyzed in Analysis 1, there was an effect of genotype (*F*(2,73) = 9.496, ***p* < 0.001), with NL-G-F/E2 mice (**G**) spending less time exploring objects than NL-G-F/E3 (**H**) and NL-G-F/E4 (**I**) mice. **J/K/L.** In NL-G-F/E2, NL-G-F/E3, and NL-G-F/E4 mice in Analysis 2, there was an effect of genotype (*F*(2,72) = 5.171, ***p* = 0.008), with NL-G-F/E2 mice (**J**) spending less time in the center containing objects than NL-G-F/E3 (*p* = 0.029, Tukey’s) (**K**) and NL-G-F/E4 mice) (*p* = 0.007, Tukey’s) (**L**).

**Fig. S3.** Time spent exploring object (**A**-**F**) and discrimination index (**G**-**L**) of KUNV- and PBS-treated NL-G-F/E2 (**A**, **D, G, J**), NL-G-F/E3 (**B**, **E**, **H**, **K**) and NL-G-F/E4 (**C**, **F**, **I**, **L**) mice in the open field containing objects. **A/B/C.** When time spent exploring the objects on the object recognition test day in NL-G-F/E2, NL-G-F/E3, and NL-G-F/E4 mice in Analysis was analyzed, there was an effect of genotype (*F*(2,73) = 9.496, ****p* < 0.001), with NL-G-F/E2 mice (**A**) spending less time exploring objects than NL-G-F/E3 (**B**) and NL-G-F/E4 (**C**) mice. **D/E/F.** When time spent exploring the objects on the object recognition test day in NL-G-F/E2, NL-G-F/E3, and NL-G-F/E4 mice in Analysis 2 was analyzed, there was an effect of genotype (*F*(2,126) = 10.954, ****p* < 0.001), with NL-G-F/E2 mice (**D**) spending less time exploring the objects than NL-G-F/E3 (*p* = 0.008, Tukey’s) (**E**) and NL-G-F/E4 mice (*p* < 0.001, Tukey’s) (**F**).

**Fig. S4.** Time spent exploring object (**A**-**D**) and discrimination index (**E**-**H**) of KUNV- and PBS-treated E3 (**A**, **C, E, G**) and E4 (**B**, **D**, **F**, **H**) mice in the open field containing objects.

**Fig. S5.** Duration in the novel arm (**A**-**F**) and percent entries into the novel arm (**G**-**L**) of KUNV- and PBS-treated NL-G-F/E2 (**A**, **D, G, J**), NL-G-F/E3 (**B**, **E**, **H**, **K**) and NL-G-F/E4 (**C**, **F**, **I**, **L**) mice in spatial Y maze test.

**Fig. S6.** Circadian body temperatures of KUNV- and PBS-treated NL-G/E4 (A) and E4 (B) mice in the fourth week following treatment in Analysis 1.

**Fig. S7.** Circadian body temperatures of KUNV- and PBS-treated NL-G/EE (A) and E4 (B) mice in the third week following treatment in Analysis 2.

**Fig. S8.** Circadian body temperatures of KUNV- and PBS-treated NL-G/E2 (A), NL-G-F/E3 (B), NL-G-F/E4 (C), and E4 (D) mice in the fourth week following treatment in Analysis 2.

**Fig. S9.** IFN-γ (**A-E**), TCR-α (**F-J**), and TNF-α (**K-O**) transcript levels in the cortex of NL-G-F/E2 (**A, F, K**), NL-G-F/E3 (**B, G, L**), NL-G-F/E4 (**C, H, M**), E3 (**D, I, N**) and E4 (**E, J, O**) mice in analysis 2. **C.** IFN-γ transcript levels were higher in NL-G-F/E4 mice with viral loads than those without it (*t* = 2.583, **p* = 0.0288, 2-tailed *t* test). **E.** IFN-γ transcript levels were higher in E4 mice with viral loads than those without it (*t* = 3.046, **p* = 0.0369, 2-tailed *t* test). **I.** TCR-α transcript levels were lower in E3 mice with viral loads than without viral loads (*t* = 2.861, **p* = 0.0134, 2-tailed *t* test).

|  | Day 1 | Day 2 | Day 3 | Day 4 | Day 5 | Day 6 | Day 7 | Day 8 |
| --- | --- | --- | --- | --- | --- | --- | --- | --- |
| NL-G-F/E4 | 35.51 ± 0.83 | 35.41 ± 0.10 | 35.36 ± 0.08 | 35.32 ± 0.11 | 35.45 ± 0.096 | 35.54 ± 0.11 | 35.57 ± 0.09 | 35.39 ± 0.11 |
| E4 | 35.41 ± 0.62 | 35.48 ± 0.19 | 35.32 ± 0.19 | 35.55 ± 0.17 | 35.58 ± 0.16 | 35.36 ± 0.17 | 35.35 ± 0.16 | 35.32 ± 0.15 |
| Significance | NS | NS | NS | NS | NS | NS | NS | NS |

**Table S1.** Comparison of body temperatures during the light periods in NL-G-F/E4 and E4 mice during week 2 of Analysis 1. ^1^

^1^ All values are in degree C. NS: not significant.

**Table S2.** Comparison of body temperatures during the dark and light periods in NL-G-F/apoE mice during week 1 of Analysis 2.

|  | Day 1 | Day 2 | Day 3 | Day 4 | Day 5 | Day 6 | Day 7 | Day 8 |
| --- | --- | --- | --- | --- | --- | --- | --- | --- |
| NL-G-F/E4 | 35.51 ± 0.83 | 35.41 ± 0.10 | 35.36 ± 0.08 | 35.32 ± 0.11 | 35.45 ± 0.096 | 35.54 ± 0.11 | 35.57 ± 0.09 | 35.39 ± 0.11 |
| E4 | 35.41 ± 0.62 | 35.48 ± 0.19 | 35.32 ± 0.19 | 35.55 ± 0.17 | 35.58 ± 0.16 | 35.36 ± 0.17 | 35.35 ± 0.16 | 35.32 ± 0.15 |
| Significance | NS | NS | NS | NS | NS | NS | NS | NS |

| **Genotype** | Day 1 | Day 2 | Day 3 | Day 4 | Day 5 | Day 6 | Day 7 | Day 8 |
| --- | --- | --- | --- | --- | --- | --- | --- | --- |
| NL-G-F/E4 | 35.51 ± 0.83 | 35.41 ± 0.10 | 35.36 ± 0.08 | 35.32 ± 0.11 | 35.45 ± 0.096 | 35.54 ± 0.11 | 35.57 ± 0.09 | 35.39 ± 0.11 |
| E4 | 35.41 ± 0.62 | 35.48 ± 0.19 | 35.32 ± 0.19 | 35.55 ± 0.17 | 35.58 ± 0.16 | 35.36 ± 0.17 | 35.35 ± 0.16 | 35.32 ± 0.15 |
| Significance | NS | NS | NS | NS | NS | NS | NS | NS |

| **Dark** | Night 2 | Night 3 | Night 4 | Night 5 | Night 6 | Night 7 | Night 8 |
| --- | --- | --- | --- | --- | --- | --- | --- |
| Males | 35.92 ± 0.07 | 35.97 ± 0.06 | 35.86 ± 0.09 | 35.92 ± 0.08 | 35.89 ± 0.09 | 35.88 ± 0.1 | 35.72 ± 0.11 |
| Females | 36.52 ± 0.06 | 36.43 ± 0.067 | 36.59 ± 0.07 | 36.56 ± 0.08 | 36.50 ± 0.09 | 36.4 ± 0.1 | 36.56 ± 0.07 |
| Significance | ***p* < 0.001** | ***p* < 0.001** | ***p* < 0.001** | ***p* < 0.001** | ***p* < 0.001** | ***p* < 0.001** | ***p* < 0.001** |

| **Light** | Day 1 | Day 2 | Day 3 | Day 4 | Day 5 | Day 6 | Day 7 | Day 8 |
| --- | --- | --- | --- | --- | --- | --- | --- | --- |
| Males | 35.76 ± 0.09 | 35.28 ± 0.06 | 35.21 ± 0.33 | 35.12 ± 0.40 | 35.20 ± 0.08 | 35.12 ± 0.08 | 35.31 ± 0.09 | 35.08 ± 0.10 |
| Females | 36.21 ± 0.04 | 35.86 ± 0.05 | 36.67 ± 0.41 | 35.71 ± 0.06 | 35.70 ± 0.06 | 35.70 ± 0.07 | 35.79 ± 0.06 | 35.81 ± 0.05 |
| Significance | ***p* < 0.001** | ***p* < 0.001** | ***p* < 0.001** | ***p* < 0.001** | ***p* < 0.001** | ***p* < 0.001** | ***p* < 0.001** | ***p* < 0.001** |

^1^ All values are in degree C. The significance *p* values are based on 2-tailed *t* tests.

**Table S3.** Comparison of body temperatures by age during the light periods in NL-G-F/apoE mice during week 1 of Analysis 2.

| **Age** | Day 1 | Day 2 | Day 3 | Day 4 | Day 5 | Day 6 | Day 7 | Day 8 |
| --- | --- | --- | --- | --- | --- | --- | --- | --- |
| Younger | 36.09 ± 0.07 | 35.60 ± 0.06 | 35.43 ± 0.06 | 35.40 ± 0.07 | 35.43 ± 0.07 | 35.35 ± 0.07 | 35.56 ± 0.06 | 35.52 ± 0.08 |
| Older | 36.02 ± 0.07 | 35.71 ± 0.07 | 35.57 ± 0.07 | 35.59 ± 0.07 | 35.61 ± 0.08 | 35.62 ± 0.09 | 35.68 ± 0.08 | 35.58 ± 0.09 |
| Significance | NS | NS | NS | NS | NS | ***p* = 0.02** | NS | NS |

^1^ All values are in degree C. The significance *p* value is based on 2-tailed *t* tests. NS: not significant.

**Table S4.** Body temperatures by age during the light periods in NL-G-F/E2 mice during week 1 of Analysis 2.

| **Age** | Day 1 | Day 2 | Day 3 | Day 4 | Day 5 | Day 6 | Day 7 | Day 8 |
| --- | --- | --- | --- | --- | --- | --- | --- | --- |
| Younger | 36.55 ± 0.11 | 35.45 ± 0.13 | 35.49 ± 0.13 | 35.49 ± 0.12 | 35.44 ± 0.09 | 35.42 ± 0.13 | 35.45 ± 0.11 | 35.47 ± 0.17 |
| Older | 35.38 ± 0.15 | 35.26 ± 0.14 | 35.23 ± 0.16 | 35.03 ± 0.15 | 34.92 ± 0.20 | 34.86 ± 0.19 | 34.95 ± 0.19 | 34.78 ± 0.20 |
| Significance | NS | NS | NS | ***p* = 0.033** | ***p* = 0.033** | ***p* = 0.027** | ***p*** **= 0.045** | ***p* = 0.021** |

^1^ All values are in degree C. The significance *p* values are based on 2-tailed *t* tests. NS: not significant.

**Table S5.** Body temperatures by age during the light periods in NL-G-F/E3 mice during week 1 of Analysis 2.

| **Age** | Day 1 | Day 2 | Day 3 | Day 4 | Day 5 | Day 6 | Day 7 | Day 8 |
| --- | --- | --- | --- | --- | --- | --- | --- | --- |
| Younger | 36.36 ± 0.06 | 35.65 ± 0.09 | 35.32 ± 0.36 | 35.29 ± 0.13 | 35.28 ± 0.14 | 35.18 ± 0.15 | 35.46 ± 0.11 | 35.73 ± 0.12 |
| Older | 36.03 ± 0.06 | 35.51 ± 0.12 | 35.27 ± 0.07 | 35.40 ± 0.13 | 35.52 ± 0.14 | 35.50 ± 0.14 | 35.63 ± 0.88 | 35.58 ± 0.11 |
| Significance | ***p* < 0.001** | NS | NS | NS | NS | NS | NS | NS |

^1^ All values are in degree C. The significance *p* value is based on 2-tailed *t* tests. NS: not significant.

**Table S6.** Body temperatures by age during the light periods in NL-G-F/E4 mice during week 1 of Analysis 2.

| **Age** | Day 1 | Day 2 | Day 3 | Day 4 | Day 5 | Day 6 | Day 7 | Day 8 |
| --- | --- | --- | --- | --- | --- | --- | --- | --- |
| Younger | 36.18 ± 0.87 | 35.64 ± 0.11 | 35.49 ± 0.88 | 35.44 ± 0.11 | 35.56 ± 0.09 | 35.44 ± 0.09 | 35.68 ± 0.09 | 35.38 ± 0.14 |
| Older | 36.26 ± 0.49 | 35.96 ± 0.07 | 35.82 ± 0.07 | 35.87 ± 0.07 | 35.91 ± 0.06 | 35.97 ± 0.08 | 35.99 ± 0.06 | 35.89 ± 0.08 |
| Significance | NS | ***p* = 0.011** | ***p* = 0.004** | ***p* = 0.002** | ***p* = 0.002** | ***p* < 0.001** | ***p* = 0.006** | ***p* < 0.001** |

^1^ All values are in degree C. The significance *p* values are based on 2-tailed *t* tests. NS: not significant.

**Table S7.** Body temperatures by sex during the light periods in NL-G-F/E4 mice during week 1 of Analysis 2.

| **Sex** | Day 1 | Day 2 | Day 3 | Day 4 | Day 5 | Day 6 | Day 7 | Day 8 |
| --- | --- | --- | --- | --- | --- | --- | --- | --- |
| Males | 36.02 ± 0.08 | 35.38 ± 0.07 | 35.34 ± 0.07 | 35.20 ± 0.08 | 35.40 ± 0.08 | 35.34 ± 0.07 | 35.57 ± 0.08 | 35.20 ± 0.15 |
| Females | 36.31 ± 0.05 | 36.03 ± 0.06 | 35.84 ± 0.06 | 35.92 ± 0.06 | 35.92 ± 0.05 | 35.94 ± 0.07 | 36.00 ± 0.06 | 35.90 ± 0.07 |
| Significance | ***p* = 0.003** | ***p* < 0.001** | ***p* < 0.001** | ***p* < 0.001** | ***p* < 0.001** | ***p* < 0.001** | ***p <* 0.001** | ***p* < 0.001** |

^1^ All values are in degree C. The significance *p* values are based on 2-tailed *t* tests. NS: not significant.

**Table S8.** Body temperatures comparing NL-G-F/E4 and E4 mice by sex during the dark periods during week 1 of Analysis 2.

| **Sex** | Night 2 | Night 3 | Night 4 | Night 5 | Night 6 | Night 7 | Night 8 |
| --- | --- | --- | --- | --- | --- | --- | --- |
| Males | 35.77 ± 0.07 | 35.80 ± 0.06 | 35.76 ± 0.10 | 35.83 ± 0.07 | 35.85 ± 0.06 | 35.91 ± 0.67 | 35.71 ± 0.10 |
| Females | 36.60 ± 0.07 | 36.57 ± 0.07 | 36.73 ± 0.07 | 36.73 ± 0.07 | 36.72 ± 0.08 | 36.69 ± 0.08 | 36.64 ± 0.08 |
| Significance | ***p <* 0.001** | ***p* < 0.001** | ***p* < 0.001** | ***p* < 0.001** | ***p* < 0.001** | ***p* < 0.001** | ***p <* 0.001** |

^1^ All values are in degree C. The significance *p* values are based on 2-tailed *t* tests. NS: not significant.

**Table S9.** Body temperatures comparing NL-G-F/E4 and E4 mice by age during the dark periods during week 1 of Analysis 2.

| **Age** | Night 2 | Night 3 | Night 4 | Night 5 | Night 6 | Night 7 | Night 8 |
| --- | --- | --- | --- | --- | --- | --- | --- |
| Younger | 36.20 ± 0.07 | 36.13 ± 0.07 | 36.25 ± 0.08 | 36.22 ± 0.08 | 36.21 ± 0.46 | 36.27 ± 0.08 | 36.07 ± 0.11 |
| Older | 36.30 ± 0.11 | 36.36 ± 0.11 | 36.8 ± 0.13 | 36.46 ± 0.11 | 36.47 ± 0.11 | 36.43 ± 0.11 | 36.38 ± 0.11 |
| Significance | ***NS*** | *p* = 0.065 | ***NS*** | *p* = 0.071 | *p* = 0.058 | ***NS*** | *p =* 0.054 |

^1^ All values are in degree C. The significance *p* values are based on 2-tailed *t* tests. NS: not significant.

**Table S10.** Body temperatures comparing NL-G-F/E4 and E4 mice by NL-G-F background during the dark periods during week 1 of Analysis 2.

| **Genotype** | Night 2 | Night 3 | Night 4 | Night 5 | Night 6 | Night 7 | Night 8 |
| --- | --- | --- | --- | --- | --- | --- | --- |
| NL-G-F/E4 | 36.48 ± 0.08 | 36.48 ± 0.08 | 36.59 ± 0.08 | 36.60 ± 0.08 | 36.57 ± 0.09 | 36.58 ± 0.09 | 36.43 ± 0.11 |
| E4 | 35.89 ± 0.09 | 35.89 ± 0.08 | 35.88 ± 0.12 | 35.95 ± 0.09 | 36.00 ± 0.09 | 36.01 ± 0.09 | 35.94 ± 0.09 |
| Significance | ***p* < 0.001** | ***p* < 0.001** | ***p* < 0.001** | ***p* < 0.001** | ***p* < 0.001** | ***p* < 0.001** | ***p =* 0.003** |

^1^ All values are in degree C. The significance *p* values are based on 2-tailed *t* tests.

**Table S11.** Body temperatures comparing NL-G-F/E4 and E4 mice by age during the light periods during week 1 of Analysis 2.

| **Age** | Day 1 | Day 2 | Day 3 | Day 4 | Day 5 | Day 6 | Day 7 | Day 8 |
| --- | --- | --- | --- | --- | --- | --- | --- | --- |
| Younger | 35.88 ± 0.10 | 35.60 ± 0.09 | 35.43 ± 0.07 | 35.44 ± 0.08 | 35.50 ± 0.07 | 35.42 ± 0.08 | 35.57 ± 0.09 | 35.43 ± 0.10 |
| Older | 35.93 ± 0.08 | 35.63 ± 0.09 | 35.57 ± 0.08 | 35.55 ± 0.09 | 35.58 ± 0.09 | 35.65 ± 0.10 | 35.70 ± 0.09 | 35.64 ± 0.08 |
| Significance | NS | NS | NS | NS | NS | ***p* = 0.086** | NS | NS |

^1^ All values are in degree C. The trend towards significance *p* value is based on a 2-tailed *t* test. NS: not significant.

**Table S12.** Body temperatures comparing NL-G-F/E4 and E4 mice by NL-G-F background during the light periods during week 1 of Analysis 2.

| **Genotype** | Day 1 | Day 2 | Day 3 | Day 4 | Day 5 | Day 6 | Day 7 | Day 8 |
| --- | --- | --- | --- | --- | --- | --- | --- | --- |
| NL-G-F/E4 | 36.23 ± 0.05 | 35.83 ± 0.06 | 35.68 ± 0.06 | 35.70 ± 0.07 | 35.76 ± 0.06 | 35.75 ± 0.07 | 35.86 ± 0.06 | 35.68 ± 0.08 |
| E4 | 35.41 ± 0.10 | 35.27 ± 0.11 | 35.22 ± 0.09 | 35.19 ± 0.10 | 35.18 ± 0.10 | 35.22 ± 0.10 | 35.28 ± 0.10 | 35.33 ± 0.10 |
| Significance | ***p* < 0.001** | ***p* < 0.001** | ***p* < 0.001** | ***p* < 0.001** | ***p* < 0.001** | ***p* < 0.001** | ***p* < 0.001** | ***p* = 0.008** |

^1^ All values are in degree C. The significance *p* values are based on 2-tailed *t* tests.

**Table S13.** Body temperatures comparing NL-G-F/apoE mice by sex during the dark periods during week 2 of Analysis 2.

| **Sex** | Night 1 | Night 2 | Night 3 | Night 4 | Night 5 | Night 6 | Night 7 | Night 8 |
| --- | --- | --- | --- | --- | --- | --- | --- | --- |
| Males | 35.90 ± 0.10 | 35.94 ± 0.08 | 35.92 ± 0.09 | 35.89 ± 0.08 | 35.88 ± 0.08 | 35.86 ± 0.08 | 35.82 ± 0.08 | 35.81 ± 0.08 |
| Females | 36.58 ± 0.06 | 36.64 ± 0.06 | 36.61 ± 0.06 | 36.56 ± 0.07 | 36.57 ± 0.07 | 36.60 ± 0.07 | 36.64 ± 0.07 | 36.63 ± 0.07 |
| Significance | ***p* < 0.001** | ***p* < 0.001** | ***p* < 0.001** | ***p* < 0.001** | ***p* < 0.001** | ***p* < 0.001** | ***p* < 0.001** | ***p <* 0.001** |

^1^ All values are in degree C. The significance *p* values are based on 2-tailed *t* tests.

**Table S14.** Body temperatures comparing NL-G-F/apoE mice by treatment during the dark periods during week 2 of Analysis 2.

| **Treatment** | Day 1 | Day 2 | Day 3 | Day 4 | Day 5 | Day 6 | Day 7 | Day 8 |
| --- | --- | --- | --- | --- | --- | --- | --- | --- |
| PBS | 36.34 ± 0.10 | 36.47 ± 0.08 | 36.46 ± 0.08 | 36.40 ± 0.09 | 36.42 ± 0.09 | 36.42 ± 0.09 | 36.39 ± 0.09 | 36.42 ± 0.09 |
| KUNV | 36.33 ± 0.08 | 36.33 ± 0.08 | 36.29 ± 0.09 | 36.27 ± 0.09 | 36.27 ± 0.09 | 36.28 ± 0.09 | 36.33 ± 0.09 | 36.29 ± 0.09 |
| Significance | NS | NS | NS | NS | NS | NS | NS | NS |

^1^ All values are in degree C. The significance *p* values are based on 2-tailed *t* tests. NS: not significant.

**Table S15.** Body temperatures comparing NL-G-F/apoE mice by treatment during the light periods during week 2 of Analysis 2.

| **Sex** | Day 1 | Day 2 | Day 3 | Day 4 | Day 5 | Day 6 | Day 7 | Day 8 |
| --- | --- | --- | --- | --- | --- | --- | --- | --- |
| Males | 35.90 ± 0.10 | 35.94 ± 0.08 | 35.92 ± 0.09 | 35.89 ± 0.08 | 35.88 ± 0.08 | 35.86 ± 0.08 | 35.82 ± 0.08 | 35.81 ± 0.08 |
| Females | 36.58 ± 0.06 | 36.64 ± 0.06 | 36.61 ± 0.06 | 36.56 ± 0.07 | 36.57 ± 0.07 | 36.60 ± 0.07 | 36.64 ± 0.07 | 36.63 ± 0.07 |
| Significance | ***p* < 0.001** | ***p* < 0.001** | ***p* < 0.001** | ***p* < 0.001** | ***p* < 0.001** | ***p* < 0.001** | ***p* < 0.001** | ***p <* 0.001** |

^1^ All values are in degree C. The significance *p* values are based on 2-tailed *t* tests.

**Table S16.** Body temperatures comparing NL-G-F/apoE mice by age during the light periods during week 2 of Analysis 2.

| **Age** | Day 1 | Day 2 | Day 3 | Day 4 | Day 5 | Day 6 | Day 7 | Day 8 |
| --- | --- | --- | --- | --- | --- | --- | --- | --- |
| Younger | 35.56 ± 0.06 | 35.48 ± 0.06 | 35.41 ± 0.06 | 35.40 ± 0.06 | 35.50 ± 0.07 | 35.52 ± 0.07 | 35.60 ± 0.07 | 35.57 ± 0.07 |
| Older | 35.62 ± 0.09 | 35.65 ± 0.09 | 35.69 ± 0.09 | 35.66 ± 0.10 | 35.71 ± 0.10 | 36.68 ± 0.11 | 35.71 ± 0.09 | 35.61 ± 0.10 |
| Significance | NS | NS | ***p* = 0.013** | ***p* = 0.028** | *p* = 0.086 | NS | NS | NS |

^1^ All values are in degree C. The significance *p* values are based on 2-tailed *t* tests. NS: not significant.

**Table S17.** Body temperatures in NL-G-F/E2 mice by treatment during the light periods during week 2 of Analysis 2.

| **Treatment** | Day 1 | Day 2 | Day 3 | Day 4 | Day 5 | Day 6 | Day 7 | Day 8 |
| --- | --- | --- | --- | --- | --- | --- | --- | --- |
| PBS | 34.88 ± 0.20 | 35.12 ± 0.16 | 35.17 ± 0.18 | 35.08 ± 0.17 | 35.07 ± 0.16 | 34.90 ± 0.16 | 35.05 ± 0.14 | 34.94 ± 0.16 |
| KUNV | 35.36 ± 0.20 | 35.29 ± 0.20 | 35.19 ± 0.17 | 35.29 ± 0.19 | 35.38 ± 0.19 | 35.31 ± 0.23 | 35.34 ± 0.20 | 35.28 ± 0.22 |
| Significance | NS | NS | ***p* = 0.013** | ***p* = 0.028** | *p* = 0.086 | NS | NS | NS |

^1^ All values are in degree C. The significance *p* values are based on 2-tailed *t* tests. NS: not significant.

**Table S18.** Body temperatures in NL-G-F/E3 mice by age during the dark periods during week 1 of Analysis 2.

| **Age** | Night 1 | Night 2 | Night 3 | Night 4 | Night 5 | Night 6 | Night 7 | Night 8 |
| --- | --- | --- | --- | --- | --- | --- | --- | --- |
| Younger | 36.38 ± 0.09 | 36.31 ± 0.07 | 36.25 ± 0.12 | 36.22 ± 0.13 | 36.30 ± 0.13 | 36.26 ± 0.08 | 36.29 ± 0.08 | 36.47 ± 0.10 |
| Older | 36.36 ± 0.12 | 36.57 ± 0.11 | 36.54 ± 0.13 | 36.47 ± 0.15 | 36.49 ± 0.13 | 36.48 ± 0.14 | 36.53 ± 0.13 | 36.52 ± 0.14 |
| Significance | NS | *p* = 0.085 | *p* = 0.070 | *p* = 0.064 | ***p* = 0.026** | ***p* = 0 .011** | NS | NS |

**Table S19.** Body temperatures in NL-G-F/E3 mice by age during the dark periods during week 1 of Analysis 2.

| **Sex** | Night 1 | Night 2 | Night 3 | Night 4 | Night 5 | Night 6 | Night 7 | Night 8 |
| --- | --- | --- | --- | --- | --- | --- | --- | --- |
| Males | 36.45 ± 0.14 | 36.35 ± 0.13 | 36.23 ± 0.18 | 36.30 ± 0.17 | 36.30 ± 0.17 | 36.19 ± 0.16 | 36.20 ± 0.17 | 36.08 ± 0.17 |
| Females | 36.34 ± 0.09 | 36.44 ± 0.08 | 36.42 ± 0.10 | 36.32 ± 0.11 | 36.40 ± 0.11 | 36.39 ± 0.09 | 36.49 ± 0.08 | 36.48 ± 0.09 |
| Significance | NS | *NS* | *NS* | *NS* | ***NS*** | ***NS*** | P = 0.092 | P = 0.035 |

**Table S20.** Body temperatures in NL-G-F/E3 mice by treatment during the dark periods during week 1 of Analysis 2.

| **Treatment** | Night 1 | Night 2 | Night 3 | Night 4 | Night 5 | Night 6 | Night 7 | Night 8 |
| --- | --- | --- | --- | --- | --- | --- | --- | --- |
| PBS | 36.52 ± 0.11 | 36.56 ± 0.11 | 36.58 ± 0.12 | 36.53 ± 0.13 | 36.63 ± 0.13 | 36.57 ± 0.11 | 36.47 ± 0.14 | 36.47 ± 0.14 |
| KUNV | 36.29 ± 0.09 | 36.33 ± 0.08 | 36.25 ± 0.12 | 36.18 ± 0.12 | 36.21 ± 0.12 | 36.19 ± 0.09 | 36.38 ± 0.09 | 36.32 ± 0.10 |
| Significance | NS | *p* = 0.085 | *p* = 0.070 | *p* = 0.064 | ***p* = 0.026** | ***p* = 0 .011** | NS | NS |

^1^ All values are in degree C. The significance *p* values are based on 2-tailed *t* tests. NS: not significant.

**Table S21.** Body temperatures in NL-G-F/E3 mice by treatment during the light periods during week 2 of Analysis 2.

| **Treatment** | Day 1 | Day 2 | Day 3 | Day 4 | Day 5 | Day 6 | Day 7 | Day 8 |
| --- | --- | --- | --- | --- | --- | --- | --- | --- |
| PBS | 35.70 ± 0.13 | 35.62 ± 0.14 | 35.74 ± 0.19 | 35.77 ± 0.18 | 35.81 ± 0.15 | 34.85 ± 0.15 | 35.78 ± 0.13 | 34.64 ± 0.17 |
| KUNV | 35.56 ± 0.11 | 35.55 ± 0.10 | 35.52 ± 0.10 | 35.37 ± 0.09 | 35.43 ± 0.12 | 35.44 ± 0.12 | 35.67 ± 0.10 | 35.60 ± 0.11 |
| Significance | NS | NS | ***NS*** | ***p* = 0.039** | *p* = 0.052 | ***p* = 0 .042** | NS | NS |

^1^ All values are in degree C. The significance *p* values are based on 2-tailed *t* tests. NS: not significant.

**Table S22.** Body temperatures in NL-G-F/E4 mice by sex during the light periods during week 2 of Analysis 2.

| **Sex** | Day 1 | Day 2 | Day 3 | Day 4 | Day 5 | Day 6 | Day 7 | Day 8 |
| --- | --- | --- | --- | --- | --- | --- | --- | --- |
| Males | 35.39 ± 0.07 | 35.25 ± 0.09 | 35.20 ± 0.06 | 35.11 ± 0.07 | 35.18 ± 0.07 | 35.22 ± 0.09 | 35.28 ± 0.09 | 35.23 ± 0.07 |
| Females | 35.92 ± 0.07 | 35.93 ± 0.06 | 35.91 ± 0.07 | 36.05 ± 0.09 | 36.05 ± 0.09 | 36.06 ± 0.08 | 36.05 ± 0.06 | 36.02 ± 0.07 |
| Significance | ***p* < 0.001** | ***p* < 0.001** | ***p* < 0.001** | ***p* < 0.001** | ***p* < 0.001** | ***p* < 0.001** | ***p* < 0.001** | ***p* < 0.001** |

^1^ All values are in degree C. The significance *p* values are based on 2-tailed *t* tests.

**Table S23.** Body temperatures in NL-G-F/E4 mice by age during the light periods during week 2 of Analysis 2.

| **Age** | Day 1 | Day 2 | Day 3 | Day 4 | Day 5 | Day 6 | Day 7 | Day 8 |
| --- | --- | --- | --- | --- | --- | --- | --- | --- |
| Younger | 35.51 ± 0.08 | 35.41 ± 0.10 | 35.36 ± 0.08 | 35.33 ± 0.11 | 35.45 ± 0.10 | 35.54 ± 0.11 | 35.57 ± 0.09 | 35.59 ± 0.11 |
| Older | 35.93 ± 0.07 | 35.93 ± 0.07 | 35.93 ± 0.08 | 35.94 ± 0.09 | 36.01 ± 0.12 | 35.98 ± 0.11 | 35.99 ± 0.09 | 35.91 ± 0.09 |
| Significance | ***p* < 0.001** | ***p* < 0.001** | ***p* < 0.001** | ***p* < 0.001** | ***p* < 0.001** | ***p* = 0.007** | ***p* = 0.003** | ***p* = 0.029** |

^1^ All values are in degree C. The significance *p* values are based on 2-tailed *t* tests.

**Table S24.** Body temperatures comparing in NL-G-F/E4 and E4 mice mice by age during the dark periods during week 2 of Analysis 2.

| **Age** | Day 1 | Day 2 | Day 3 | Day 4 | Day 5 | Day 6 | Day 7 | Day 8 |
| --- | --- | --- | --- | --- | --- | --- | --- | --- |
| Younger | 35.92 ± 0.06 | 35.90 ± 0.07 | 35.91 ± 0.07 | 35.86 ± 0.06 | 35.83 ± 0.08 | 35.80 ± 0.08 | 35.76 ± 0.07 | 35.76 ± 0.08 |
| Older | 36.69 ± 0.08 | 36.75 ± 0.08 | 36.71 ± 0.08 | 36.72 ± 0.09 | 36.70 ± 0.09 | 36.75 ± 0.08 | 36.74 ± 0.08 | 36.74 ± 0.08 |
| Significance | ***p* < 0.001** | ***p* < 0.001** | ***p* < 0.001** | ***p* < 0.001** | ***p* < 0.001** | ***p* < 0.001** | ***p* < 0.001** | ***p* < 0.001** |

^1^ All values are in degree C. The significance *p* values are based on 2-tailed *t* tests.

**Table S25.** Body temperatures comparing in NL-G-F/E4 and E4 mice mice by sex during the light periods during week 2 of Analysis 2.

| **Sex** | Day 1 | Day 2 | Day 3 | Day 4 | Day 5 | Day 6 | Day 7 | Day 8 |
| --- | --- | --- | --- | --- | --- | --- | --- | --- |
| Males | 35.16 ± 0.07 | 35.08 ± 0.07 | 34.97 ± 0.07 | 34.98 ± 0.06 | 35.03 ± 0.07 | 34.98 ± 0.08 | 34.98 ± 0.08 | 35.01 ± 0.08 |
| Females | 35.89 ± 0.06 | 35.92 ± 0.06 | 35.90 ± 0.07 | 35.97 ± 0.07 | 36.0 ± 0.08 | 36.01 ± 0.07 | 36.01 ± 0.06 | 35.97 ± 0.06 |
| Significance | ***p* < 0.001** | ***p* < 0.001** | ***p* < 0.001** | ***p* < 0.001** | ***p* < 0.001** | ***p* < 0.001** | ***p* < 0.001** | ***p* < 0.001** |

^1^ All values are in degree C. The significance *p* values are based on 2-tailed *t* tests.

**Table S26.** Principle components analysis: component loadings for behavioral, genotype, body temperatures, cortical soluble and insoluble Ab and p-tau levels, and cortical immune measures^1^.

| *Measure* | *Factor 1* | *Factor 2* | *Factor 3* | *Factor 4* | *Factor 5* | *Factor 6* | *Factor 7* | *Factor 8* |
| --- | --- | --- | --- | --- | --- | --- | --- | --- |
| ApoE Genotype | **0.501** |  |  |  |  |  |  | **-0.649** |
| Distance moved in the open field on day 1 |  | **0.802** |  |  |  |  |  |  |
| Time spent in the center of the open field on day 1 |  |  |  |  | **0.595** |  |  |  |
| Difference in activity levels in the open field between days 1 and 2 |  |  |  |  |  | **-0.648** |  |  |
| Arm entries in the Y maze |  | **0.751** |  |  |  |  |  |  |
| Spontaneous alternation in the Y maze |  |  |  |  | **0.754** | **-** |  |  |
| Time spent in the novel arm in the spatial Y maze |  |  |  |  |  |  | **0.709** |  |
| Soluble p-tau |  | **0.635** |  |  |  |  |  |  |
| Insoluble p-tau |  |  |  |  |  |  |  | **0.792** |
| Soluble Aβ40 |  |  |  |  |  |  | **0.648** |  |
| Insoluble Aβ40 |  |  |  |  |  | **0.695** |  |  |
| Soluble Aβ42 |  |  |  | **0.830** |  |  |  |  |
| Insoluble Aβ42 |  |  |  | **0.799** |  |  |  |  |
| IFN-γ |  |  | **0.797** |  |  |  |  |  |
| TCR-α |  |  | **0.781** |  |  |  |  |  |
| TNF-α |  |  | **0.566** |  |  |  |  |  |
| Average body temperature in the dark periods in week 1 | **0.904** |  |  |  |  |  |  |  |
| Average body temperature in the dark periods in week 2 | **0.935** |  |  |  |  |  |  |  |
| Average body temperature in the dark periods in week 4 | **0.910** |  |  |  |  |  |  |  |
| Average body temperature in the light periods in week 1 | **0.908** |  |  |  |  |  |  |  |
| Average body temperature in the light periods in week 2 | **0.898** |  |  |  |  |  |  |  |
| Average body temperature in the light periods in week 4 | **0.899** |  |  |  |  |  |  |  |

^1^The varimax rotated matrix was used to interpret the factor loadings. Loadings greater than 0.5 are indicated in bold. Treatment did not load on any factor.

**Table S27.** Principle components analysis: component loadings for behavioral, genotype, treatment, body temperatures, cortical soluble and insoluble p-tau levels and cortical immune measures^1^.

| *Measure* | *Factor 1* | *Factor 2* | *Factor 3* | *Factor 4* | *Factor 5* | *Factor 6* | *Factor 7* |
| --- | --- | --- | --- | --- | --- | --- | --- |
| ApoE Genotype |  |  | -0.495 |  |  |  |  |
| Treatment |  | **0.638** |  |  |  |  |  |
| Distance moved in the open field on day 1 |  |  | **0.845** |  |  |  |  |
| Time spent in the center of the open field on day 1 |  |  |  |  |  |  | **0.822** |
| Difference in activity levels in the open field between days 1 and 2 |  |  |  |  | **-0.751** |  | 0.422 |
| Arm entries in the Y maze |  |  | **0.794** |  |  |  |  |
| Spontaneous alternation in the Y maze |  | -0.407 |  | **-0.662** |  |  |  |
| Time spent in the novel arm in the spatial Y maze |  |  |  | **0.829** |  |  |  |
| Soluble p-tau |  |  |  |  |  | **0.770** |  |
| Insoluble p-tau |  |  |  |  |  | **0.791** |  |
| IFN-γ |  | **0.832** |  |  |  |  |  |
| TCR-α |  |  |  |  | **0.779** |  |  |
| TNF-α |  | **0.618** |  |  |  |  |  |
| Average body temperature in the dark periods in week 1 | **0.962** |  |  |  |  |  |  |
| Average body temperature in the dark periods in week 2 | **0.962** |  |  |  |  |  |  |
| Average body temperature in the dark periods in week 4 | **0.936** |  |  |  |  |  |  |
| Average body temperature in the light periods in week 1 | **0.902** |  |  |  |  |  |  |
| Average body temperature in the light periods in week 2 | **0.899** |  |  |  |  |  |  |
| Average body temperature in the light periods in week 4 | **0.944** |  |  |  |  |  |  |

^1^The varimax rotated matrix was used to interpret the factor loadings. Loadings greater than 0.5 are indicated in bold.

**Table S28.** Principle components analysis: component loadings for behavioral, genotype, treatment, body temperatures, cortical soluble and insoluble p-tau levels and cortical immune measures^1^.

| *Measure* | *Factor 1* | *Factor 2* | *Factor 3* | *Factor 4* | *Factor 5* | *Factor 6* |
| --- | --- | --- | --- | --- | --- | --- |
| ApoE Genotype |  | -**0.535** |  |  |  |  |
| NL-G-F |  | -0.451 |  |  |  |  |
| Treatment |  |  | **0.522** |  | -0.405 |  |
| Distance moved in the open field on day 1 |  | **0.774** |  |  |  |  |
| Time spent in the center of the open field on day 1 |  |  |  | **0.670** |  |  |
| Difference in activity levels in the open field between days 1 and 2 |  |  |  | **0.741** |  |  |
| Arm entries in the Y maze |  | **0.744** |  |  |  |  |
| Spontaneous alternation in the Y maze |  |  |  |  |  | **-0.638** |
| Time spent in the novel arm in the spatial Y maze |  |  |  |  |  | **0.643** |
| Soluble p-tau |  | **0.519** |  |  |  |  |
| Insoluble p-tau |  |  |  |  | **0.830** |  |
| IFN-γ |  |  | **0.782** |  |  |  |
| TCR-α |  |  | **0.747** |  |  |  |
| TNF-α |  |  | **0.576** |  |  |  |
| Average body temperature in the dark periods in week 1 | **0.922** |  |  |  |  |  |
| Average body temperature in the dark periods in week 2 | **0.949** |  |  |  |  |  |
| Average body temperature in the dark periods in week 4 | **0.924** |  |  |  |  |  |
| Average body temperature in the light periods in week 1 | **0.912** |  |  |  |  |  |
| Average body temperature in the light periods in week 2 | **0.909** |  |  |  |  |  |
| Average body temperature in the light periods in week 4 | **0.916** |  |  |  |  |  |

^1^The varimax rotated matrix was used to interpret the factor loadings. Loadings greater than 0.5 are indicated in bold.
